# Supramolecular Large Nanosheets Assembled at Air/Water Interfaces and in Solution from Amphiphilic Heptagon-Containing Nanographenes

**DOI:** 10.1021/acs.joc.3c01854

**Published:** 2023-12-13

**Authors:** Arthur H. G. David, Mari C. Mañas-Torres, Marcos D. Codesal, Irene López-Sicilia, María T. Martín-Romero, Luis Camacho, Juan M. Cuerva, Victor Blanco, Juan J. Giner-Casares, Luis Álvarez de Cienfuegos, Araceli G. Campaña

**Affiliations:** † Departamento de Química Orgánica, Facultad de Ciencias, Unidad de Excelencia Química Aplicada a Biomedicina y Medioambiente, Universidad de Granada, Avda. Fuente Nueva, s/n, 18071 Granada, Spain; ‡ Departamento de Química Física y T. Aplicada, Instituto Químico para la Energía y Medioambiente IQUEMA, Facultad de Ciencias, Universidad de Córdoba, Campus de Rabanales, Ed. Marie Curie, E-14071 Córdoba, Spain

## Abstract

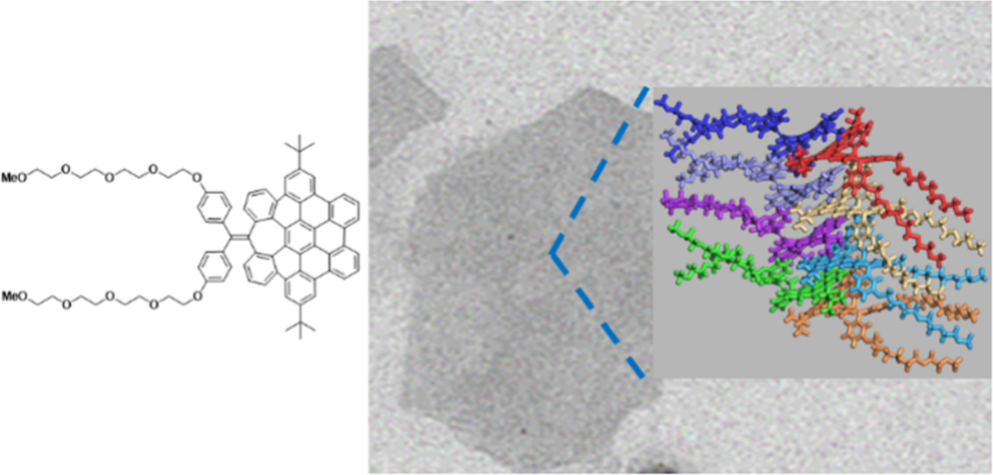

We report the synthesis of a new set of amphiphilic saddle-shaped heptagon-containing polycyclic aromatic hydrocarbons (PAHs) functionalized with tetraethylene glycol chains and their self-assembly into large two-dimensional (2D) polymers. An in-depth analysis of the self-assembly mechanism at the air/water interface has been carried out, and the proposed arrangement models are in good agreement with the molecular dynamics simulations. Quite remarkably, the number and disposition of the tetraethylene glycol chains significantly influence the disposition of the PAHs at the interface and conditionate their packing under pressure. For the three compounds studied, we observed three different behaviors in which the aromatic core is parallel, perpendicular, and tilted with respect to the water surface. We also show that these curved PAHs are able to self-assemble in solution into remarkably large sheets of up to 150 μm^2^. These results show the relationship, within a family of curved nanographenes, between the monomer configuration and their self-assembly capacity in air/water interfaces and organic–water mixtures.

## Introduction

Bottom-up approaches have allowed the development of novel synthetic methodologies with the aim of obtaining polycyclic aromatic hydrocarbons (PAHs) or nanographenes (NGs) in a well-defined manner having different sizes, shapes, lengths, widths, or edge structures.^1−3^ Starting from coronene or hexa-*peri*-hexabenzocoronene (HBC), as basic structural units, to the synthesis of extended PAHs or large graphene nanoribbons (GNR), this approach offers the possibility of obtaining these molecules in sufficient quantity for their suitable characterization, allowing a better understanding of their optoelectronic properties.^4−6^ Moreover, this approach also offers the opportunity to functionalize PAHs to afford nanostructured materials with potential applications in electronics at the nanoscale.^7,8^ In this sense, the introduction of flexible chains into the aromatic core has been an excellent strategy to obtain supramolecular polymers by a self-assembly process.^9−15^ On the other hand, distorted NGs, which combine in their structures hexagonal and nonhexagonal rings, show physical properties that differ from their planar analogues, and thus, their synthesis and study are also needed.^3,16^ Nevertheless, unlike the extensive studies carried out in the self-assembly of planar PAHs, less work has been conducted in distorted NGs, in particular, in those molecules containing seven-membered rings and presenting negative curvature.^16−18^ In this sense, distorted NG derivatives having the capacity to self-assemble into supramolecular polymers are scarce.^19^ Much of this work has focused on pentagon-containing corannulene derivatives.^20−23^ Thus, recently, Itami et al. have reported a warped NG able to self-assemble into a one-dimensional supramolecular polymer without the need of assisting substituents.^24^ Quite remarkably, in this case, the self-assembly process is challenged due to the intrinsic distortion of the molecules and their greater solubility in most organic solvents. Moreover, Rickhaus and co-workers have studied the self-assembly of saddle-shaped macrocycles bearing carbazole and pyridine units.^25^

In recent years, our group has developed a versatile strategy for the synthesis of heptagon-containing NGs.^26^ Thus, notable examples of saddle-helix hybrid NGs as distorted molecules with enhanced solubility and interesting optical properties have been presented.^27−29^ Recently, we decided to turn our attention to the supramolecular chemistry of these structures, tackling the self-association^30^ and host–guest complexation processes of distorted NGs containing only heptagonal carbocycles as nonhexagonal rings.^31,32^ In particular, we demonstrated that simple heptagon-containing HBC derivatives self-assemble in CDCl_3_ solution upon concentration increase, although with low self-association constants and forming small-sized aggregates.^30^

**Figure 1:**
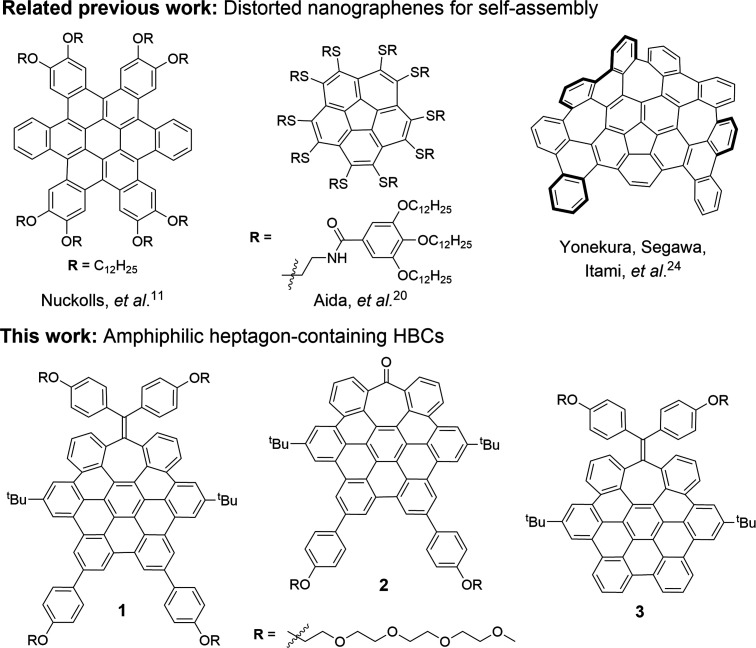
Selected previously reported distorted NGs that participate in self-assembly (top) and new amphiphilic heptagon-containing saddle-shaped HBCs **1**–**3** studied in this work (bottom).

Herein, we present the first study of the self-assembly of amphiphilic heptagon-containing NGs bearing flexible tetraethylene glycol chains (compounds **1**–**3**, Figure 1). We show that these saddle-shaped HBCs can self-assemble both at the air/water interface and in THF/H_2_O mixtures. At the interface, they form two-dimensional (2D) supramolecular polymers. Extra-large nanosheets were also observed in solution, in contrast to the usual formation of fibers by purely hexagonal aromatics. Moreover, the obtained nanosheets are persistent and show great robustness. The molecular arrangement of monomers is proposed based on experimental results supported by molecular dynamics (MD) simulations.

## Results and Discussion

### Design

The possibility of obtaining 2D polymers by a self-assembly process starting from simple monomers in solution is still a challenge.^33−35^ Nevertheless, the expected properties of these materials make them a priority target.^36−40^ Considering the high solubility of distorted NGs in most organic solvents, we decided to introduce hydrophilic ethylene glycol chains to increase the amphiphilic character of the molecules and promote the self-assembly in organic solvents and/or in mixtures of organic solvents and water.^15,41,42^

We designed three heptagon-containing saddle-shaped HBCs bearing tetraethylene glycol chains in the most planar region (compound **2**), in the more distorted seven-membered ring side (compound **3**), or on both sides of the NG structure (compound **1**), with the aim of studying the possible influence of the position of the hydrophilic chains on the self-assembly process (Figure 1).

### Synthesis and Characterization

**Scheme 1:**
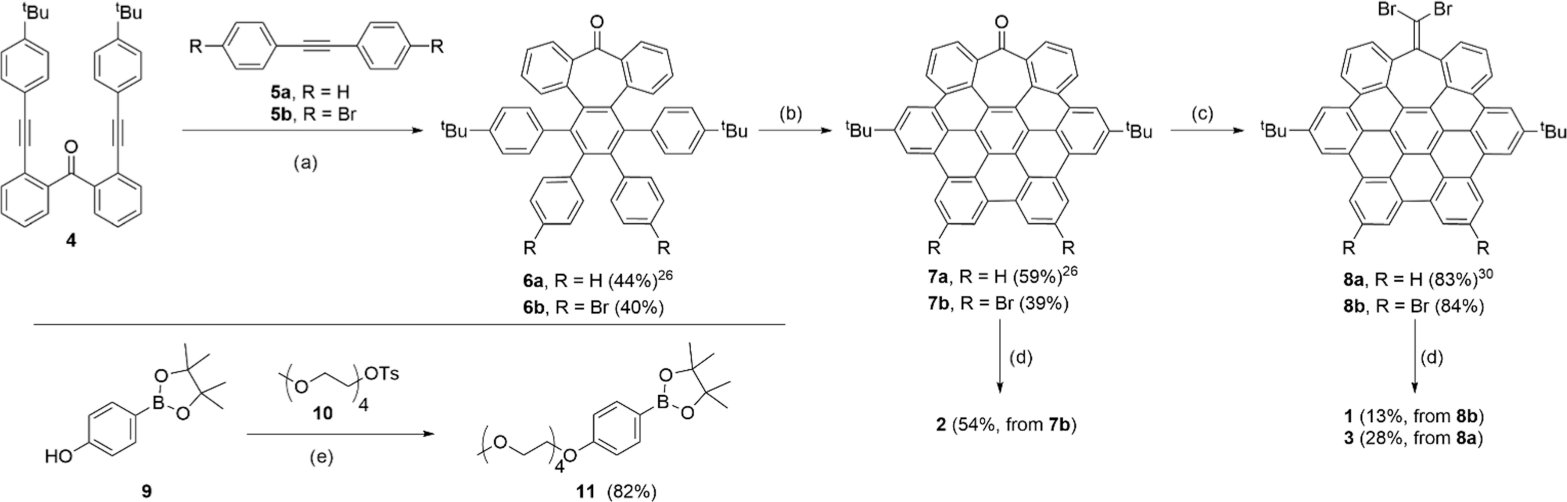
Synthesis of Amphiphilic Heptagon-Containing Nanographenes **1–3** Reagents and conditions: (a) for **6a** (from ref (26)): Co_2_(CO)_8_, dioxane, 100 °C, 16 h. For **6b**: Co_2_(CO)_8_, toluene, 110 °C, 18 h; (b) 2,3-dichloro-5,6-dicyano-1,4-benzoquinone (DDQ), CF_3_SO_3_H, CH_2_Cl_2_, 0 °C, 10 min; (c) PPh_3_, CBr_4_, toluene, 120 °C, 20 h; (d) **11**, Pd(PPh_3_)_4_, K_2_CO_3_, EtOH/H_2_O/toluene, reflux, 20 h; (e) K_2_CO_3_, DMF, 80 °C, 18 h.

The synthesis of these compounds followed a protocol previously optimized by us for the synthesis of distorted HBCs,^26^ based on the Co-catalyzed cyclotrimerization between a benzophenone dialkyne derivative (**4**) and a suitable diphenylacetylene (**5**), followed by a Scholl-type dehydrogenation reaction (Scheme 1). Compound **5b** allowed us the introduction of bromine atoms in the more planar region of the aromatic core, while the reaction of the ketone moiety with CBr_4_ and triphenylphosphine functionalized the distorted side. Suzuki coupling of resulting distorted NGs **7b** and **8a,b** with **11** afforded the target compounds. Derivatives **1**–**3** were unambiguously characterized by NMR and IR spectroscopies and HRMS, with excellent agreement between experimental and theoretical isotopic distributions (see the Supporting Information).

Besides, we also evaluated the optical properties in solution. Both UV–vis and fluorescence spectra of compounds **1**–**3** in THF solution were recorded, showing a profile similar to that of previously described heptagon-containing HBCs without the presence of long polar chains.^26,43^ UV–vis spectra of **1**–**3** show an absorption band between 300 and 450 nm (λ_max_ = 358, 361, and 350 nm, ε = 1.4 × 10^5^, 1.2 × 10^5^, and 1.2 × 10^5^ for **1**–**3**, respectively). Upon irradiation at the absorption maxima, **1**–**3** show luminescence, with a structured emission band in the 400–600 nm region, with maxima at 485, 486, and 453 nm, respectively (see the Supporting Information). The quantum yields were determined as 4.6%, 2.0%, and 5.6% for compounds **1**, **2**, and **3**, respectively (see the Supporting Information).

### Self-Assembly at the Air/Water Interface

Having synthesized and characterized compounds **1**–**3**, we evaluated their self-assembly capacity. Considering the good solubility of the synthesized compounds in a great variety of organic solvents (such as hexane, cyclohexane, toluene, dioxane, or THF) or mixtures of them tested, both at room temperature and after heating, we decided to start the study by forcing aggregation under more restricted conditions, i.e., in an air/water interface in which the anisotropy of the interface in combination with the surface pressure exerted induces molecular aggregation due to the amphiphilic nature of these molecules as well as the size and spatial distribution of their hydrophilic and hydrophobic regions. On that basis, the air/water interface allows control to a certain extent on such molecular arrangements by modifying the surface area. In such a way, the self-assembly at the air/water interface and in the bulk solution cannot be considered equivalent, as discussed below. As far as we know, this is the first report where distorted nanographenes with well-defined aggregation are formed at the air/water interface, with only some related helicene-based structures reported in the literature.^44−47^

**Figure 2:**
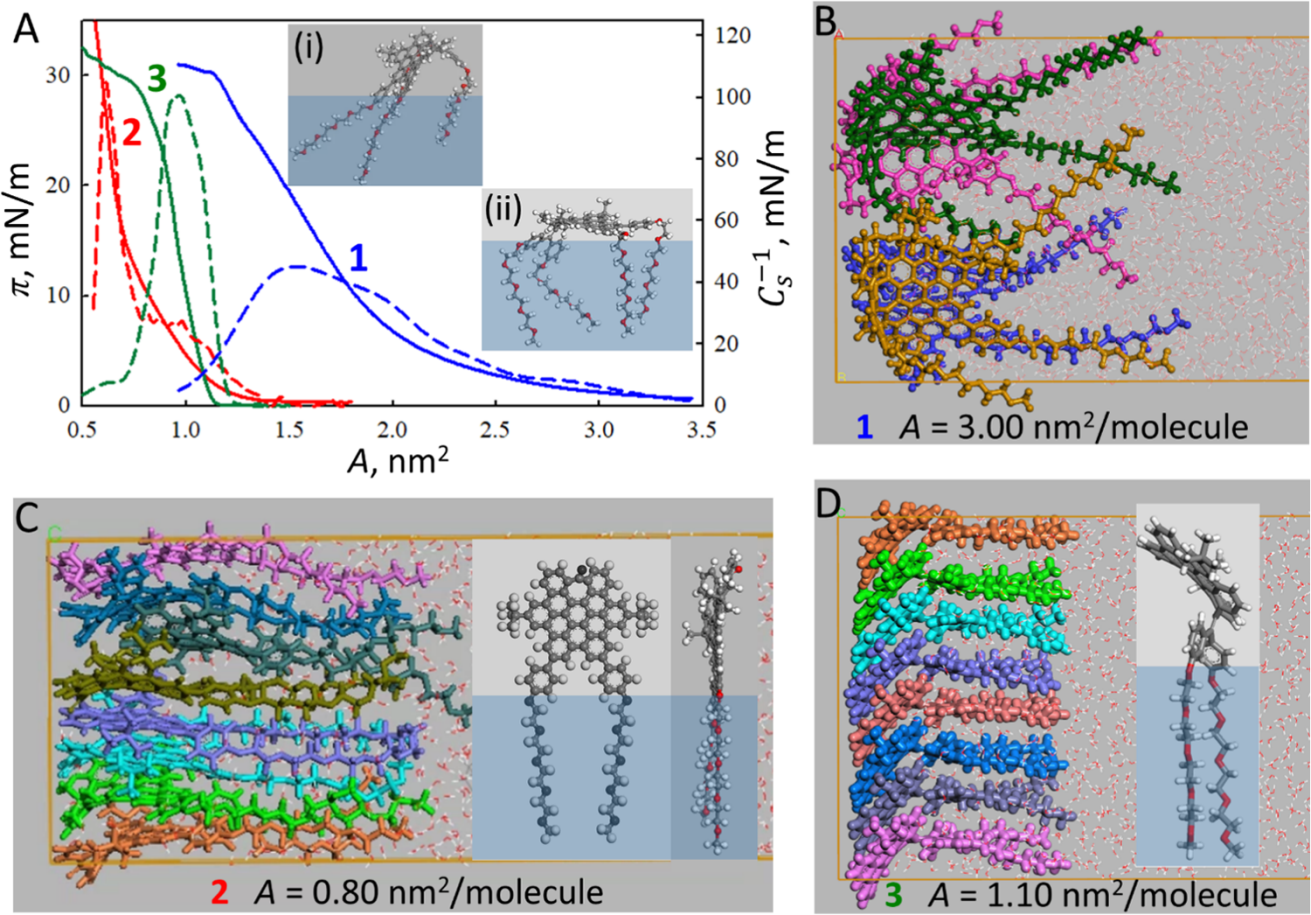
(A) π–*A* isotherms (blue, red, and green solid lines for **1**, **2**, and **3**, respectively, left axis); *C*_s_^–1^–*A* isotherms (blue, red, and green dashed lines for **1**, **2**, and **3**, respectively, right axis). Insets: molecular organization of compound **1** at high (i) and low (ii) surface pressures. (B–D) Models inferred from simulations of monolayers of **1**, **2**, and **3**, respectively.

**Figure 3:**
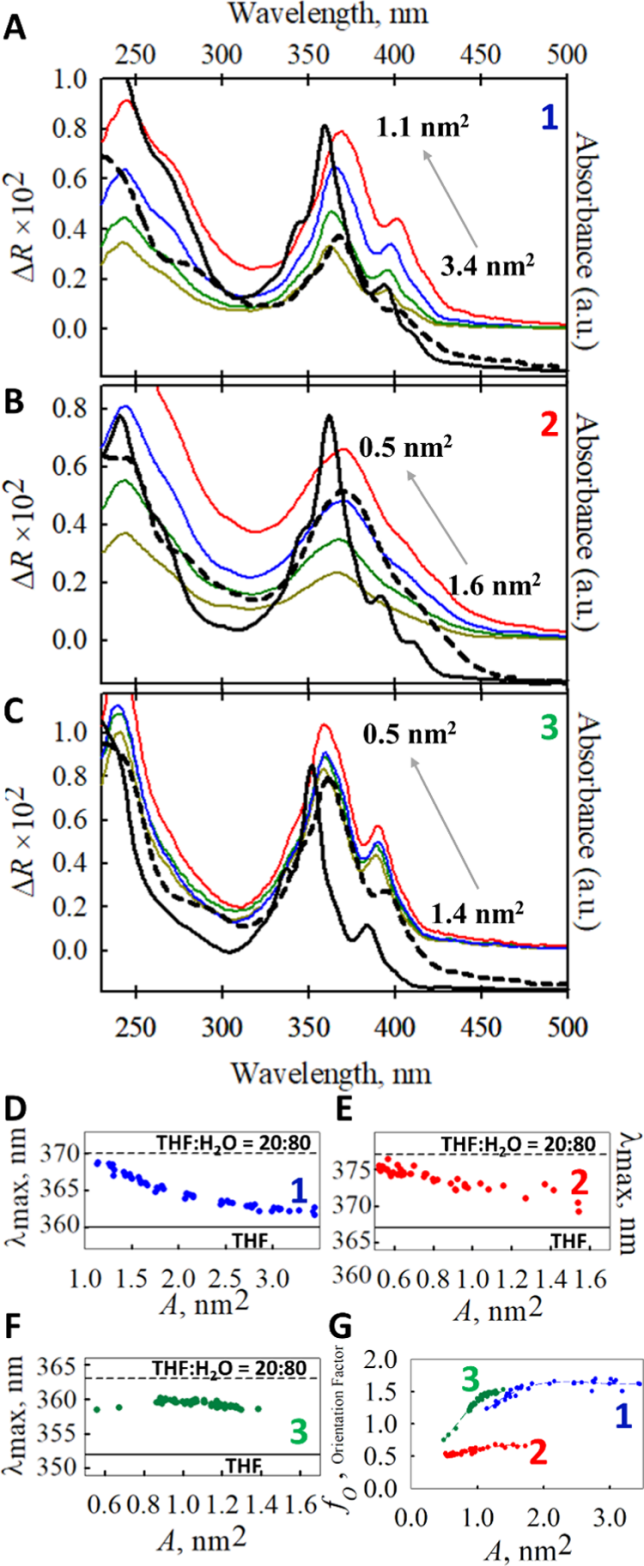
(A–C) UV–vis reflection spectra under compression of the films assembled from **1**, **2**, and **3**, respectively (left axis). For comparison, the absorption spectra of such compounds in solution (THF and THF/H_2_O 20:80, solid and dashed black lines, respectively, right axis) are shown. Panels (D–F) show the evolution of λ_max_ during the compression process of the monolayers of **1, 2**, and **3**, respectively. For comparison, the λ_max_ values of the UV–vis spectra in THF and THF/H_2_O 20:80 are noted. (G) Plots of the variation of the orientation factor versus surface area for the three nanographene derivatives.

Supramolecular arrangements of compounds **1**–**3** leading to nanosheets were studied by combining surface experimental techniques at the air/water interface, i.e., surface pressure (π)–area (*A*) and reflection spectroscopy (Δ*R*) with computer simulations. Initially, we evaluated the surface pressure (π) vs surface area (*A*) isotherms for compounds **1**–**3**, which are shown in Figure 2A (blue, red, and green solid lines for **1**, **2**, and **3**, respectively). All compounds form stable monolayers at the air–water interface, with no hysteresis found after successive compression–expansion cycles (data not shown). Also, the *in situ* UV–vis reflection spectra simultaneously measured at the air/water interface confirmed no loss of molecules into the bulk water subphase (Figure 3), as discussed below. The π*–A* isotherms allowed us to study two significant properties of the self-assembly of compounds **1**–**3** that provided quantitative insights into the molecular arrangement. The first one is the surface area at which the surface pressure begins to increase, i.e., the minimum area at which molecules show resistance to be compressed, being *A* ≈ 3.5 nm^2^, *A* ≈ 1.5 nm^2^, and *A* ≈ 1.2 nm^2^ for **1**, **2**, and **3**, respectively. The second is the π_c_ and *A* at the monolayer collapse, where the film breaks down, π_c_ ∼ 30 mN/m and *A* ∼ 1.13 nm^2^, for **1**, at π_c_ ∼ 40 mN/m and *A* ∼ 0.5 nm^2^, for **2**, and at π_c_ ∼ 30 mN/m and *A* ∼ 0.7 nm^2^, for **3**. These results show clear differences among the supramolecular arrangements of the three compounds, from a looser packing of compound **1** to a tighter one for compound **3**.

Regarding the amphiphilic properties of such molecules, compound **1** possesses four hydrophilic chains distributed around the hydrophobic region of the molecule. This special geometry could be understood using an ideal model with the four chains immersed in the aqueous subphase and the hydrophobic region located parallel to the interface, like a stool-shaped conformation (Figure 2A, inset at high surface area). As the surface pressure increases, the main axis of the hydrophobic region of the molecule tilts while maintaining the hydrophilic chains immersed in the aqueous subphase (Figure 2A, inset at low surface area, extracted from the MD simulation). This model is inferred from the simulations performed on the monolayers of **1** by molecular dynamics (Figure 2B).

Compound **2** incorporates a different hydrophobic region compared to compounds **1** and **3**, as it lacks the 1,1-bis(4-alkoxyphenyl)ethylene group, and consequently, its hydrophobic region is smaller and flatter than those of **1** and **3**. Although the planarity of the NG region shows some distortion, the hydrophilic and hydrophobic regions of compound **2** can be perpendicular and simultaneously oriented with respect to the interface (Figure 2C, left), and therefore, a lower surface area per molecule is occupied, not only at low surface pressure but also at the collapse. Additionally, the model of the monolayer of compound **2** inferred from molecular dynamics (Figure 2C, with the view from different perspectives) confirms such a suggestion. Compound **3** has two hydrophilic chains in the most distorted part of the 7-membered ring, so its hydrophilic and hydrophobic regions cannot be simultaneously oriented to the interface in a perpendicular manner but tilted (Figure 2D, left). However, as inferred from molecular dynamics simulations (Figure 2D, right), this does not prevent the stacking of the NG regions.

Furthermore, the resistance of a monolayer to the molecular packing at the air–water interface can be inferred from the compressibility modulus (*C*_s_^–1^), defined by eq 1.
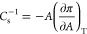
1

According to the literature,^48^ the values of the compressibility modulus for thin films range from 12.5 to 50 mN m^–1^ for a liquid-expanded, and from 100 to 250 mN m^–1^ for a liquid-condensed phase, respectively. The plots of *C*_s_^–1^*vs A* for compounds **1**–**3** are shown in Figure 2A (blue, red, and green dashed lines for **1**, **2**, and **3**, respectively). The maximum values of the compressibility modulus take place at: *C*_s_^–1^ ≈ 50 mN m^–1^ (*A* ∼ 1.5 nm^2^) for **1**, *C*_s_^–1^ ≈ 105 mN m^–1^ (*A* ∼ 0.61 nm^2^) for **2**, and *C*_s_^–1^ ≈ 100 mN m^–1^ (*A* ∼ 0.96 nm^2^) for **3**. These results indicate that **1** formed less rigid monolayers (in the liquid-expanded phase) than those formed by **2** and **3** (liquid-condensed films). This fact is related to the spatial orientation imposed at **1** by the air–water interface (Figure 2A, inset), and thus, the aggregation of NG core is not facilitated. On the other hand, the monolayers formed by **2** and **3** have similar rigidity that can be related to the aggregation of their NG core.

Simultaneously to the π*–A* isotherm measurements, UV–visible reflection spectra have been recorded at the air–water interface for the films assembled from **1**, **2**, and **3**. The UV–vis reflection signal does not consider any contribution from the bulk solution but is generated by the increase in reflection of the incoming radiation due to the absorption of molecules present at the air–liquid interface, as indicated by eq 2.^49^
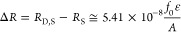
2where Δ*R* is the increase of reflection under normal incidence, *R*_S_ and *R*_D,S_ are the intensities of reflection of incoming radiation in the absence and presence of a Langmuir monolayer, respectively; ε is the molar absorption coefficient in solution and in aggregation absence (THF solution) with units M^–1^ cm^–1^; *A* is the area occupied per chromophore molecule in nm^–2^; and *f*_O_ is the orientation factor, a dimensionless parameter which compares the average orientation of the dipole transition at the air–liquid interface with respect to the random orientation in the bulk solution.^50^ For monolayers, Δ*R* values are about 10^–3^, so they are usually expressed in % (Δ*R ×* 100).

Figure 3A–C shows the reflection spectra of compounds **1**–**3** at different surface areas along with the spectra of such compounds in THF and THF/H_2_O 20:80 solutions (where aggregates are formed, as discussed below in the Self-Assembly Studies in Solution Section) as reference. Under compression–expansion cycles, the reflection spectra are coincident and evidence no loss of chromophores toward the aqueous subphase.

Figure 3D–F shows the variation of the wavelength of the maximum of the reflection spectrum (λ_max_) that corresponds to the most intense band in the visible region (band centered at ∼360 nm) as a function of surface area. Again, the maximum of the absorption spectra in THF, in the absence of aggregation (solid black line), and in THF/H_2_O 20:80, where aggregates are formed (dashed black lines), are shown for comparison purposes. Accordingly, the λ_max_ value provides us information about the aggregation of such different compounds at the air–water interface. Thus, for compounds **1** and **2**, λ_max_ increases as the surface area decreases, observing a red shift from no-or low-aggregation, approx. λ_max_ in THF solution, to an aggregation similar to that observed in THF/H_2_O 20:80 solutions (see below) (Figure 3D,E).

However, compound **3** exhibits a quite different behavior with respect to those in **1** and **2**. The λ_max_ remained almost constant during the compression process. Additionally, the λ_max_ value is intermediate to that observed in THF and THF/H_2_O 20:80 solutions. This behavior indicates the molecules of **3** at the air–water interface aggregate through a different route than in solution. Furthermore, this aggregation cannot be controlled by modifying the surface area.

Moreover, the orientation factor can be estimated at λ_max_ using eq 3
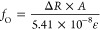
3

For uniaxial films: *f*_O_ = 3*P*(θ)/2, where *P*(θ) = ⟨sin(θ)^2^⟩, is the order parameter, being θ the angle between the normal to the air–liquid interface and the direction of the transition dipole of the absorption band, and brackets indicate average values.^50^ Thus, for the uniaxial distribution of rodlike molecules, like nematic liquid crystals, *f*_O_ can take values between 1.5 and 0. For biaxial degenerated films: *f*_O_ = 3[1+*P*(θ)]/4,^50^ where θ is the angle between the normal to the air–liquid interface and the plane formed by the two degenerated absorption modes. Thus, for biaxial distributions such as porphyrins, *f*_O_ can take values between 1.5 (flat orientation with respect to the air–water interface) and 0.75 (perpendicular orientation with respect to the interface).

The absorption band observed in the reflection and absorption spectra at λ > 300 nm could be assigned to absorption modes of the NG core region. The NG region of molecules **1**–**3** could be considered as a quasi-plane, not-degenerate biaxial system, so quantitative predictions on the orientation factor from the previous model (degenerate biaxial flat molecules) are not possible. Note also that the theoretical expressions of *f*_O_ are valid for simple absorption components, while the experimental absorption bands of compounds **1**–**3** are generally due to several overlapping components. Nevertheless, the biaxial degenerated film model can be applied qualitatively to our system.

For compound **1** (Figure 3G, blue dots and line) at *A* > 1.75 nm^2^, the orientation factor was experimentally estimated as *f*_O_ ≈ 1.6. The results suggest a quasiflat orientation of the NG region with respect to the interface. This observation is consistent with the values of surface area per molecule observed in the isotherm. The decrease of *f*_O_ values under compression indicates a tilt of the absorption components, as discussed above. For compound **2**, *f*_O_ oscillates between 0.7 and 0.5 (Figure 3G, red dots and line), indicating an almost perpendicular orientation of the NG groups with respect to the interface, regardless of the surface area. For molecule **3**, *f*_O_ decreases from 1.5 to 0.75 (Figure 3G, green dots and line) as the surface area decreases from 1.4 nm^2^ to 0.5 nm^2^. Therefore, a progressive inclination of the chromophore molecules is expected. The different *f*_O_ values for compounds **1**–**3** agree, at least qualitatively, with the orientation models at the air–water interface deduced from the simulations by molecular dynamics (Figure 3B–D).

**Figure 4:**
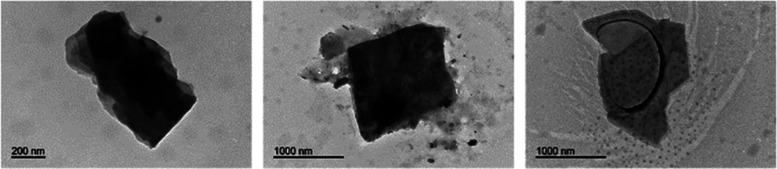
TEM pictures of transferred films of **3** from the air–water interface onto a TEM grid.

Finally, films of **3** were transferred by the Langmuir–Schaefer method to solid supports and analyzed by transmission electron microscopy (TEM). The presence of nanosheets can be observed from the images obtained, showing that the well-ordered self-assembled arrangement is preserved during the transfer process (Figure 4). This suggests that the assembly into nanosheets might not be induced only in the restrained conditions of the water/air interface but also in solution, which makes the study of self-assembly in the latter worthwhile. Emission spectra of the films of **3** were similar to those obtained in bulk solution (see below) (Figure S1). Low crystallinity was observed, as shown by the blank diffractogram of the substrates, as only one 2θ peak was observed at 22°, corresponding to a *d*-spacing of 4.05 nm (Figure S2).

### Self-Assembly Studies in Solution

Knowing that these compounds have the ability to self-assemble at the air–water interface to form well-ordered structures, we next studied their self-assembly under unrestrained conditions in solution, promoting their self-assembly only by changing the solubility of the molecules. Compounds **1**–**3** were completely soluble in THF at room temperature. However, upon mixing those solutions with water at increasing ratios, we observed that at concentrations in the 10^–4^ M range, cloudy suspensions appeared. These suspensions were stable over time, and no precipitate was formed (see the Supporting Information, Figure S3).

This aggregation process was studied by ^1^H NMR (see the Supporting Information, Figures S4–S6) and UV–vis and fluorescence spectroscopies (see the Supporting Information, Figures S8–S10) analyzing the spectra recorded in solvent mixtures of THF with increasing water ratio. The ^1^H NMR spectra of compounds **1**–**3** (2.5 mM) in THF-*d*_8_/D_2_O mixtures (from 100:0 to 40:60) showed an upfield shift of the aromatic signals upon an increase in the water ratio. The shift toward lower frequencies observed is more pronounced at higher D_2_O ratios and is combined with a clear broadening of the signals observed in the spectra recorded in THF-*d*_8_/D_2_O 60:40 and 40:60 (Figures S4–S6). This behavior is in line with the aggregation of the compounds driven by the establishment of π–π interactions between the heptagon-containing HBC units and the hydrophobic effect. In addition, these results are in agreement with our previous study on the self-assembly of a series of compounds based on the same heptagon-containing HBC core, however, without the tetraethylene glycol chains.^30^ In that work, we observed a shift toward lower frequencies of the aromatic signals of the NGs upon increase of the concentration of the compounds in CDCl_3_, showing their self-association through the establishment of π–π interactions. In this way, both the increase of the concentration and the solvent polarity are factors that should promote the aggregation process as they both favor the interaction between the aromatic cores, which is supported by the same effect observed in the ^1^H NMR spectra.

The UV–vis and fluorescence spectra of compounds **1**–**3** (ca. 1 × 10^–4^ M) recorded in THF/H_2_O mixtures (100:0 to 20:80) afforded similar conclusions to those derived from the NMR experiments. In the absorption spectra, we can observe a clear absorbance flattening and broadening at the higher water ratios tested, which can be explained by the self-assembly of the compounds to form aggregates. In the same way, fluorescence spectra show a quenching of the emission, more intense as the water proportion increased, attributed to the aggregation process through an aggregation-caused quenching (ACQ) mechanism (Figures S7–S9).

**Figure 5:**
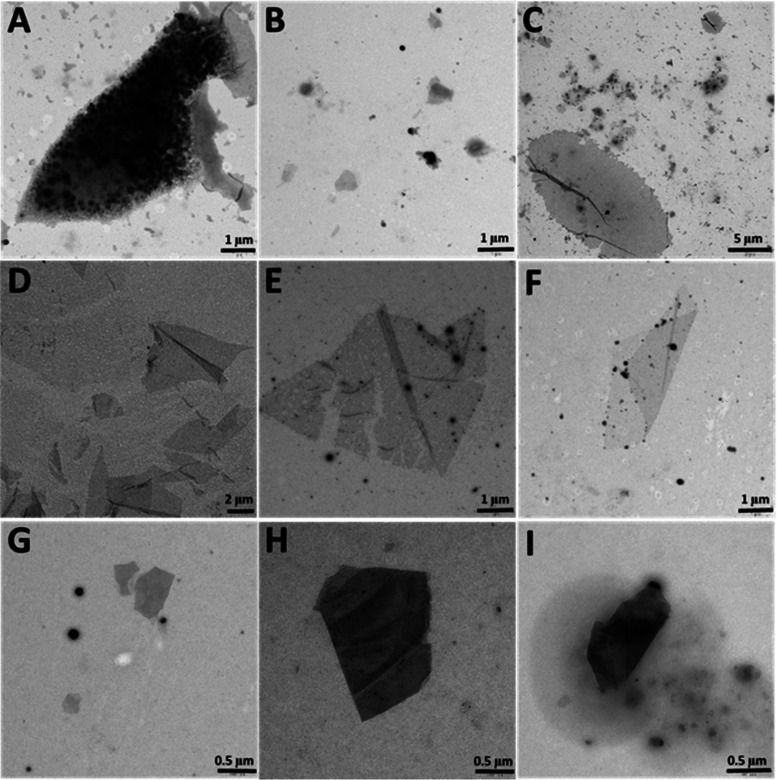
TEM images of nanosheets formed by **1** (A–C), **2** (D–F), and **3** (G–I) in mixtures of THF/H_2_O (20:80). Images were taken after 1 week of incubation, except picture (A) that was taken after 24 h.

In order to study the morphology of the self-assembled structures, suspensions of **1** in THF/H_2_O mixtures in 30:70, 20:80, and 10:90 ratios were evaluated by TEM. TEM images of the air-dried samples after incubation for 24 h showed the presence of different aggregates in which nanospheres comprising diameters between 50 and 300 nm were clearly identified (see the Supporting Information, Figure S10A,B,J). Interestingly, the mixture THF/H_2_O at a 20:80 ratio also showed the appearance of nanosheets close to aggregates of nanospheres, similar to those observed from the transfer of the films from the air/water interface (Figure 5A). The presence of these sheets close to nanospheres suggests that nanospheres can evolve to more stable nanosheets, as it has been previously described.^51−53^ Thus, we incubated the suspension for a period of 1 week and then analyzed it by TEM. In this case, the proportion of nanosheets vs nanospheres was higher, and in some of the explored areas, only nanosheets were observed (Figure 5B,D). Interestingly, some of these sheets were extremely large, more than 15 μm, comprising an area exceeding 150 μm^2^ (Figure 5C).

To further study the nature of the assembled species over time, we have analyzed by TEM aliquots from a sample of compound **1** in THF/water 20:80 taken at different times (1d, 4d, 6d, and 11d, Figure S11). The results showed that nanospheres are formed initially (Figure S11A). After 4–6 days, nanospheres were still predominant, but some small sheets were formed (Figure S11B,C). However, after 11 days of incubation (Figure S11D), large nanosheets were present as the major self-assembled structure, and the amount of nanospheres is clearly lower. This experiment points to the self-assembly leading initially toward the formation of nanospheres, which evolve with time to assembled nanosheets. The lack of nanosheets (or their reduced size) at short incubation times discards the simultaneous formation of both kinds of assemblies from the beginning.

Compounds **2** (Figure 5D–F) and **3** (Figure 5G–I) were also able to form large nanosheets after 1 week of incubation. Although the number of sheets was higher in the case of **2**, reaching also great dimensions (up to 80 μm^2^), unfortunately, some of them appeared fragmented, folded, and with pores (Figure 5D–F). However, these defects might also have been formed in the process of deposition and drying required for TEM recording. In the case of **3**, TEM images show that some of the obtained nanosheets seem to be stacked in more than one layer (Figure 5G–I). High-Resolution TEM analysis of these nanosheets showed the absence of a diffraction pattern similar to those transferred from the interface.

**Figure 6:**
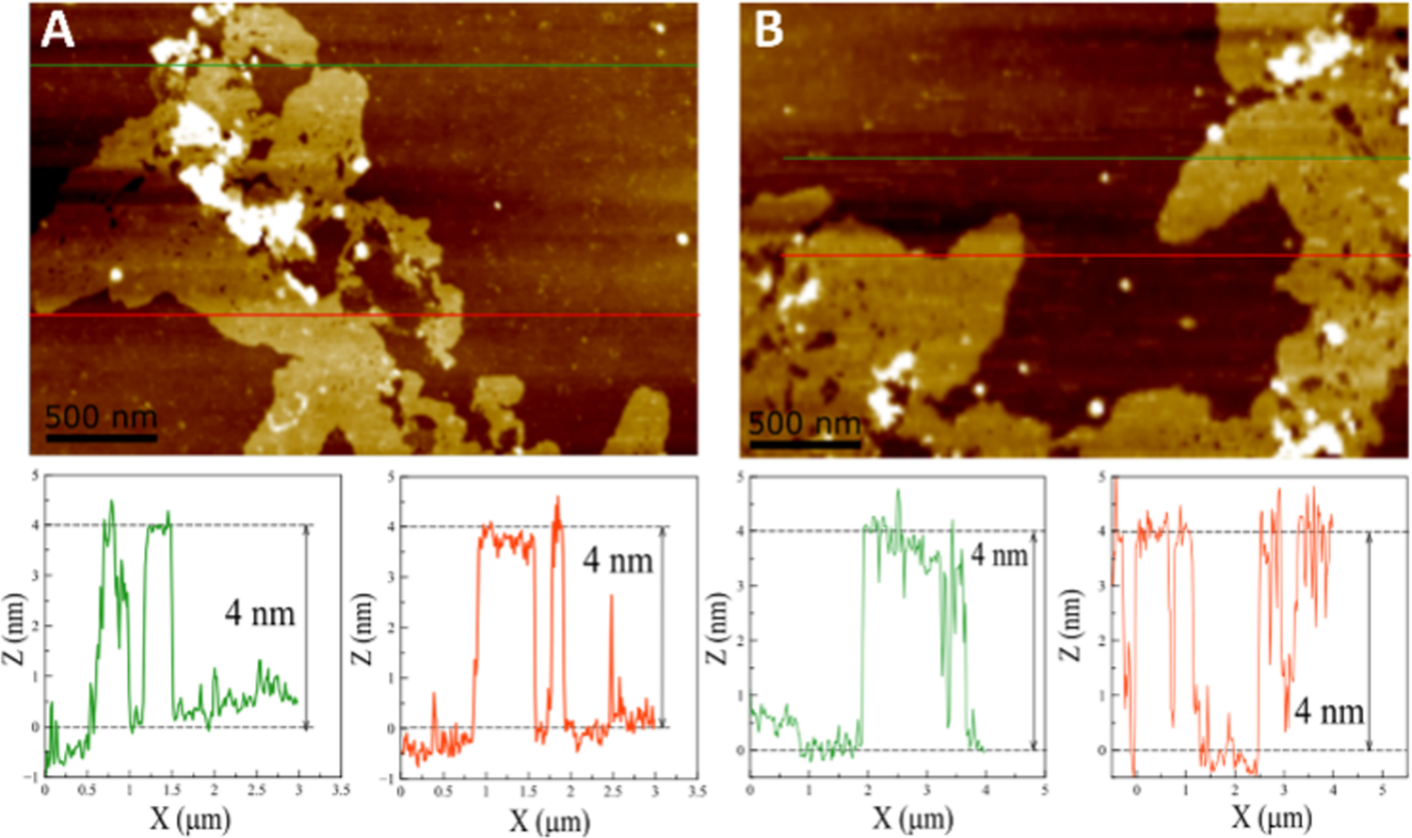
(A, B) AFM images of representative 2D nanosheets formed from **3** in THF/H_2_O (20:80) (top). The thickness of the assemblies obtained (bottom).

**Figure 7:**
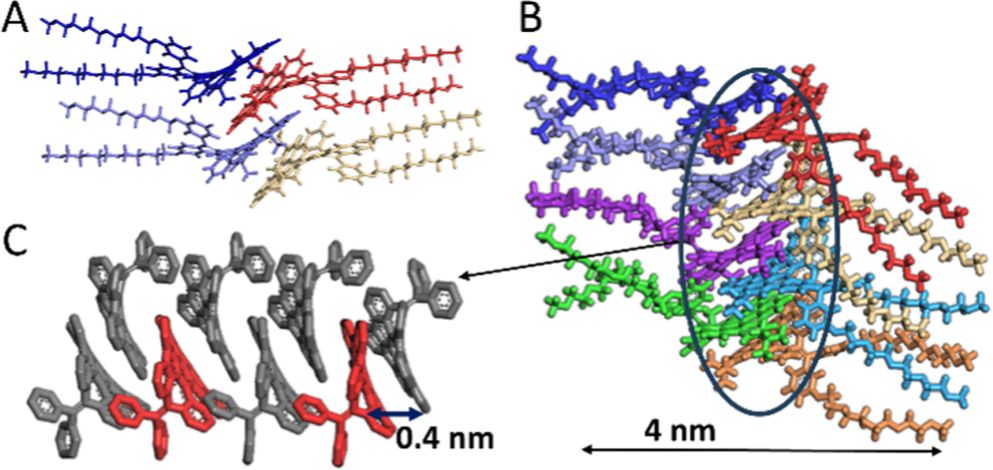
Proposed model of 2D self-assembly for compound **3**. (A) initial structures with distorted planes of nanographenes and the ethylene glycol chains in all-trans conformation; (B, C) structures extracted during the simulation process. Solvent molecules are not included for better visualization of the type of aggregation formed. (C) shows only the NG cores of the formed aggregate with two molecules depicted in red for clarity.

Atomic Force Microscopy (AFM) measurements of the obtained nanosheets were also performed. The observation of clear nanosheets was, however, possible only for compound **3** (Figure 6). In the samples analyzed by AFM, the nanosheets showed a constant thickness of 4 ± 0.2 nm, corresponding to a possible monolayer, as discussed below. Würthner and others^41,42,54^ have shown that the self-assembly of amphiphilic π-conjugated systems in mixtures of organic–water solvents is mediated by the favored π–π interactions reinforced by solvophobic interactions in water.^55,56^ Therefore, by increasing the proportion of water, the packing of these molecules is favored by π–π interactions between the NGs, the hydrophobic effect, and the disposition of the ethylene glycol chains outward, increasing the solvation in water (see the proposed model, Figure 7). Remarkably, reports of 2D polymers formed by a self-assembly process in solution to afford large sheets of similar sizes as the ones obtained here are very rare.^34,35^

Simulations by molecular dynamics were carried out to try to elucidate the molecular packaging of these compounds. In each case, the initial structures were built by locating the distorted planes of the nanographenes at a distance of ca. 0.6 nm. The ethylene glycol chains were built in the all-trans conformation (Figures 7A and S12A,C, for **3**, **1**, and **2**, respectively). For **2** and **3**, the planes are rotated 180°, giving rise to alternating arrangements. These structures were immersed in a periodic box where the solvent (THF/H_2_O 20:80) was incorporated. The structure was subsequently optimized, with several MD cycles being carried out. Representative images extracted during the simulation process are shown in Figure 7B,C (compound **3)** and S12 (compounds **1**, **2**). For compound **3**, this image shows how both NGs and phenylethylene groups participate in molecular aggregation, with the separation between molecules being about 0.4 nm. In this case, the aggregation pattern of compound **3** differs from the one proposed in the interface (see Figure 2D), which is expected. The proposed 1D aggregate in solution is almost 4 nm wide, including the tetraethylene glycol chains (Figure 7B), which matches the thickness of the nanosheets measured by AFM. In this case, the aromatic cores are placed in the interior of the supramolecular structure, leaving the polar ethylene glycol chains outward on both sides, in agreement with models previously proposed in the literature for the self-assembly in water or THF/water mixtures of derivatives with an aromatic core functionalized with polar groups, where such hydrophilic moieties were oriented toward the upper and lower surfaces of the nanosheet.^34,35,37^ The structure of the observed 2D nanosheets is supported by the interaction between the new lateral hydrophobic surfaces of those 1D assemblies, as reported by Rybtchinski and co-workers.^37^ They showed how 1D assemblies of amphiphilic perylendiimide formed 2D monolayers in water or THF/water by the interaction between the new hydrophobic surfaces bearing alkyl groups formed upon 1D self-assembly. The same interaction can apply in our system to hold the 2D structure, as a hydrophobic surface bearing t-butyl groups is present in the 1D assemblies resulting from the interaction of the NG cores.

## Conclusions

A set of three amphiphilic saddle-shaped NGs for directed self-assembly were synthesized. The intermolecular interaction driving the self-assembly process was ascribed to the interactions between the curved NG cores and the disposition of the hydrophilic tetraethylene glycol chains in the organic–water mixtures. For the first time, the self-assembly of functionalized distorted heptagon-containing NGs has been studied in the air/water interface. Remarkably, the saddle-shaped core promoted the formation of 2D nanosheets for all NGs both after incubation in the THF/water mixture and in the air/water interphase. Moreover, the saddle-shaped core also determines the disposition of the ethylene glycol chains in the process of self-assembly, giving rise to three different modes of aggregation alluding to the number and location of the chains in the NGs structure. Although this effect cannot be studied in detail in solution, a better understanding is obtained at the air/water interface. Experimental results supported by molecular dynamics simulations have given clear evidence, at the molecular level, of the different aggregation modes for the three studied compounds. These differences have an impact on the rigidity of the monolayers formed.

Furthermore, the obtained supramolecular assemblies showed great robustness independent of the available surface area at the water surface, with no loss of chromophores after compression–expansion cycles.

Using a combination of in situ spectroscopy at the air/water interface and computer simulations, we could convincingly describe the detailed arrangement of **1**–**3** in the obtained nanosheets. To the best of our knowledge, this is the first example of the self-assembly of curved nanographene into sheets rather than into the commonly found fibers. This approach offers valuable insights for the design of carbon nanostructures that efficiently self-assemble into novel supramolecular structures without the need for a template. The use of distorted molecular NGs in supramolecular assemblies is still in its infancy, although it might open new avenues for potential energy applications such as photocatalysis.^57−59^

## Data Availability

The data underlying this study are available in the published article and its Supporting Information.

## Supporting Information Available

Experimental and synthetic procedures, characterization details of new compounds (NMR, HRMS, IR, UV–vis, and fluorescence spectra), and additional supporting figures and computational methods (PDF)

## References

[ref1] Dötz F.; Brand J. D.; Ito S.; Gherghel L.; Müllen K. Synthesis of Large Polycyclic Aromatic Hydrocarbons: Variation of Size and Periphery. J. Am. Chem. Soc. 2000, 122, 7707–7717. 10.1021/ja000832x.

[ref2] Narita A.; Feng X.; Müllen K. Bottom-up Synthesis of Chemically Precise Graphene Nanoribbons. Chem. Rec. 2015, 15, 295–309. 10.1002/tcr.201402082. 25414146

[ref3] Segawa Y.; Ito H.; Itami K. Structurally Uniform and Atomically Precise Carbon Nanostructures. Nat. Rev. Mater. 2016, 1, 15002 10.1038/natrevmats.2015.2.

[ref4] Watson M. D.; Fechtenkötter A.; Müllen K. Big Is Beautiful - “Aromaticity” Revisited from the Viewpoint of Macromolecular and Supramolecular Benzene Chemistry. Chem. Rev. 2001, 101, 1267–1300. 10.1021/cr990322p. 11710221

[ref5] Chen L.; Hernandez Y.; Feng X.; Müllen K. From Nanographene and Graphene Nanoribbons to Graphene Sheets: Chemical Synthesis. Angew. Chem., Int. Ed. 2012, 51, 7640–7654. 10.1002/anie.201201084. 22777811

[ref6] Narita A.; Wang X. Y.; Feng X.; Müllen K. New Advances in Nanographene Chemistry. Chem. Soc. Rev. 2015, 44, 6616–6643. 10.1039/C5CS00183H. 26186682

[ref7] Wu J.; Pisula W.; Müllen K. Graphenes as Potential Material for Electronics. Chem. Rev. 2007, 107, 718–747. 10.1021/cr068010r. 17291049

[ref8] Moulin E.; Busseron E.; Giuseppone N. Supramolecular Materials for Opto-Electronics; Koch N., Ed.; RSC Smart Materials Series; Royal Society of Chemistry, 2015; pp 1–52.

[ref9] Debije M. G.; Piris J.; De Haas M. P.; Warman J. M.; Tomović Ž.; Simpson C. D.; Watson M. D.; Müllen K. The Optical and Charge Transport Properties of Discotic Materials with Large Aromatic Hydrocarbon Cores. J. Am. Chem. Soc. 2004, 126, 4641–4645. 10.1021/ja0395994. 15070380

[ref10] Kumar S. Self-Organization of Disc-like Molecules: Chemical Aspects. Chem. Soc. Rev. 2006, 35, 83–109. 10.1039/B506619K. 16365644

[ref11] Xiao S.; Tang J.; Beetz T.; Guo X.; Tremblay N.; Siegrist T.; Zhu Y.; Steigerwald M.; Nuckolls C. Transferring Self-Assembled, Nanoscale Cables into Electrical Devices. J. Am. Chem. Soc. 2006, 128, 10700–10701. 10.1021/ja0642360. 16910663

[ref12] Sergeyev S.; Pisula W.; Geerts Y. H. Discotic Liquid Crystals: A New Generation of Organic Semiconductors. Chem. Soc. Rev. 2007, 36, 1902–1929. 10.1039/b417320c. 17982517

[ref13] Laschat S.; Baro A.; Steinke N.; Giesselmann F.; Hägele C.; Scalia G.; Judele R.; Kapatsina E.; Sauer S.; Schreivogel A.; Tosoni M. Discotic Liquid Crystals: From Tailor-Made Synthesis to Plastic Electronics. Angew. Chem., Int. Ed. 2007, 46, 4832–4887. 10.1002/anie.200604203. 17568461

[ref14] Feng X.; Pisula W.; Kudernac T.; Wu D.; Zhi L.; De Feyter S.; Müllen K. Controlled Self-Assembly of *C*_*3*_-Symmetric Hexa-*peri*-hexabenzocoronenes with Alternating Hydrophilic and Hydrophobic Substituents in Solution, in the Bulk, and on a Surface. J. Am. Chem. Soc. 2009, 131, 4439–4448. 10.1021/ja808979t. 19271704

[ref15] Jin W.; Yamamoto Y.; Fukushima T.; Ishii N.; Kim J.; Kato K.; Takata M.; Aida T. Systematic Studies on Structural Parameters for Nanotubular Assembly of Hexa-*peri*-hexabenzocoronenes. J. Am. Chem. Soc. 2008, 130, 9434–9440. 10.1021/ja801179e. 18576635

[ref16] Márquez I. R.; Castro-Fernández S.; Millán A.; Campaña A. G. Synthesis of Distorted Nanographenes Containing Seven- and Eight-Membered Carbocycles. Chem. Commun. 2018, 54, 6705–6718. 10.1039/C8CC02325E. 29799051

[ref17] Miao Q. Heptagons in Aromatics: From Monocyclic to Polycyclic. Chem. Rec. 2015, 15, 1156–1159. 10.1002/tcr.201510009. 26443195

[ref18] Chaolumen; Stepek I. A.; Yamada K. E.; Ito H.; Itami K. Construction of Heptagon-Containing Molecular Nanocarbons. Angew. Chem., Int. Ed. 2021, 60, 23508–23532. 10.1002/anie.202100260. 33547701

[ref19] Xiao S.; Myers M.; Miao Q.; Sanaur S.; Pang K.; Steigerwald M. L.; Nuckolls C. Molecular Wires from Contorted Aromatic Compounds. Angew. Chem., Int. Ed. 2005, 44, 7390–7394. 10.1002/anie.200502142. 16173105

[ref20] Miyajima D.; Tashiro K.; Araoka F.; Takezoe H.; Kim J.; Kato K.; Takata M.; Aida T. Liquid Crystalline Corannulene Responsive to Electric Field. J. Am. Chem. Soc. 2009, 131, 44–45. 10.1021/ja808396b. 19128171

[ref21] Shoji Y.; Kajitani T.; Ishiwari F.; Ding Q.; Sato H.; Anetai H.; Akutagawa T.; Sakurai H.; Fukushima T. Hexathioalkyl Sumanenes: An Electron-Donating Buckybowl as a Building Block for Supramolecular Materials. Chem. Sci. 2017, 8, 8405–8410. 10.1039/C7SC03860G. 29619187 PMC5863616

[ref22] Nagano T.; Nakamura K.; Tokimaru Y.; Ito S.; Miyajima D.; Aida T.; Nozaki K. Functionalization of Azapentabenzocorannulenes by Fivefold C–H Borylation and Cross-Coupling Arylation: Application to Columnar Liquid-Crystalline Materials. Chem. - Eur. J. 2018, 24, 14075–14078. 10.1002/chem.201803676. 30043435

[ref23] Kang J.; Miyajima D.; Mori T.; Inoue Y.; Itoh Y.; Aida T. A Rational Strategy for the Realization of Chain-Growth Supramolecular Polymerization. Science 2015, 347, 646–651. 10.1126/science.aaa4249. 25657246

[ref24] Kato K.; Takaba K.; Maki-Yonekura S.; Mitoma N.; Nakanishi Y.; Nishihara T.; Hatakeyama T.; Kawada T.; Hijikata Y.; Pirillo J.; Scott L. T.; Yonekura K.; Segawa Y.; Itami K. Double-Helix Supramolecular Nanofibers Assembled from Negatively Curved Nanographenes. J. Am. Chem. Soc. 2021, 143, 5465–5469. 10.1021/jacs.1c00863. 33759524

[ref25] Woods J. F.; Gallego L.; Pfister P.; Maaloum M.; Vargas Jentzsch A.; Rickhaus M. Shape-Assisted Self-Assembly. Nat. Commun. 2022, 13, 3681 10.1038/s41467-022-31482-2. 35760814 PMC9237116

[ref26] Márquez I. R.; Fuentes N.; Cruz C. M.; Puente-Muñoz V.; Sotorrios L.; Marcos M. L.; Choquesillo-Lazarte D.; Biel B.; Crovetto L.; Gómez-Bengoa E.; González M. T.; Martin R.; Cuerva J. M.; Campaña A. G. Versatile Synthesis and Enlargement of Functionalized Distorted Heptagon-Containing Nanographenes. Chem. Sci. 2017, 8, 1068–1074. 10.1039/C6SC02895K. 28451246 PMC5357993

[ref27] Cruz C. M.; Márquez I. R.; Mariz I. F. A.; Blanco V.; Sánchez-Sánchez C.; Sobrado J. M.; Martín-Gago J. A.; Cuerva J. M.; Maçôas E.; Campaña A. G. Enantiopure Distorted Ribbon-Shaped Nanographene Combining Two-Photon Absorption-Based Upconversion and Circularly Polarized Luminescence. Chem. Sci. 2018, 9, 3917–3924. 10.1039/C8SC00427G. 29780523 PMC5934837

[ref28] Cruz C. M.; Castro-Fernández S.; Maçôas E.; Cuerva J. M.; Campaña A. G. Undecabenzo[7]Superhelicene: A Helical Nanographene Ribbon as a Circularly Polarized Luminescence Emitter. Angew. Chem., Int. Ed. 2018, 57, 14782–14786. 10.1002/anie.201808178. 30144368

[ref29] Cruz C. M.; Márquez I. R.; Castro-Fernández S.; Cuerva J. M.; Maçôas E.; Campaña A. G. A Triskelion-Shaped Saddle–Helix Hybrid Nanographene. Angew. Chem., Int. Ed. 2019, 58, 8068–8072. 10.1002/anie.201902529. 30968999

[ref30] David A. H. G.; Míguez-Lago S.; Cruz C. M.; Cuerva J. M.; Blanco V.; Campaña A. G. Heptagon-Containing Saddle-Shaped Nanographenes: Self-Association and Complexation Studies with Polycyclic Aromatic Hydrocarbons and Fullerenes. Org. Mater. 2021, 03, 51–59. 10.1055/s-0041-1722848.

[ref31] Jiménez V. G.; David A. H. G.; Cuerva J. M.; Blanco V.; Campaña A. G. A Macrocycle Based on a Heptagon-Containing Hexa-*peri*-hexabenzocoronene. Angew. Chem., Int. Ed. 2020, 59, 15124–15128. 10.1002/anie.202003785. 32428338

[ref32] Mora-Fuentes J. P.; Codesal M. D.; Reale M.; Cruz C. M.; Jiménez V. G.; Sciortino A.; Cannas M.; Messina F.; Blanco V.; Campaña A. G. A Heptagon-Containing Nanographene Embedded into [10]Cycloparaphenylene. Angew. Chem., Int. Ed. 2023, 62, e202301356 10.1002/anie.202301356. 36944060

[ref33] Sakamoto J.; Van Heijst J.; Lukin O.; Schlüter A. D. Two-Dimensional Polymers: Just a Dream of Synthetic Chemists?. Angew. Chem., Int. Ed. 2009, 48, 1030–1069. 10.1002/anie.200801863. 19130514

[ref34] Vybornyi M.; Rudnev A. V.; Langenegger S. M.; Wandlowski T.; Calzaferri G.; Häner R. Formation of Two-Dimensional Supramolecular Polymers by Amphiphilic Pyrene Oligomers. Angew. Chem., Int. Ed. 2013, 52, 11488–11493. 10.1002/anie.201307029. 24108690

[ref35] Vybornyi M.; Rudnev A.; Häner R. Assembly of Extra-Large Nanosheets by Supramolecular Polymerization of Amphiphilic Pyrene Oligomers in Aqueous Solution. Chem. Mater. 2015, 27, 1426–1431. 10.1021/acs.chemmater.5b00047.

[ref36] Zhang X.; Xie Y. Recent Advances in Free-Standing Two-Dimensional Crystals with Atomic Thickness: Design, Assembly and Transfer Strategies. Chem. Soc. Rev. 2013, 42, 8187–8199. 10.1039/c3cs60138b. 23887238

[ref37] Shahar C.; Baram J.; Tidhar Y.; Weissman H.; Cohen S. R.; Pinkas I.; Rybtchinski B. Self-Assembly of Light-Harvesting Crystalline Nanosheets in Aqueous Media. ACS Nano 2013, 7, 3547–3556. 10.1021/nn400484y. 23521176

[ref38] Weingarten A. S.; Kazantsev R. V.; Palmer L. C.; McClendon M.; Koltonow A. R.; Samuel A. P. S.; Kiebala D. J.; Wasielewski M. R.; Stupp S. I. Self-Assembling Hydrogel Scaffolds for Photocatalytic Hydrogen Production. Nat. Chem. 2014, 6, 964–970. 10.1038/nchem.2075. 25343600 PMC4326083

[ref39] Bonaccorso F.; Colombo L.; Yu G.; Yu G.; Stoller M.; Stoller M.; Tozzini V.; Tozzini V.; Ferrari A. C.; Ferrari A. C.; Ruoff R. S.; Ruoff R. S.; Pellegrini V. Graphene, Related Two-Dimensional Crystals, and Hybrid Systems for Energy Conversion and Storage. Science 2015, 347, 1246501 10.1126/science.1246501. 25554791

[ref40] Li Z.; Lin Z. Two-Dimensional Polymers: Synthesis and Applications. ACS Appl. Mater. Interfaces 2021, 13, 45130–45138. 10.1021/acsami.1c12392. 34524804

[ref41] Chen Z.; Fimmel B.; Würthner F. Solvent and Substituent Effects on Aggregation Constants of Perylene Bisimide π-Stacks - A Linear Free Energy Relationship Analysis. Org. Biomol. Chem. 2012, 10, 5845–5855. 10.1039/c2ob07131b. 22391667

[ref42] Görl D.; Würthner F. Entropically Driven Self-Assembly of Bolaamphiphilic Perylene Dyes in Water. Angew. Chem., Int. Ed. 2016, 55, 12094–12098. 10.1002/anie.201606917. 27558471

[ref43] Reale M.; Sciortino A.; Cannas M.; Maçoas E.; David A. H. G.; Cruz C. M.; Campaña A. G.; Messina F. Atomically Precise Distorted Nanographenes: The Effect of Different Edge Functionalization on the Photophysical Properties down to the Femtosecond Scale. Materials 2023, 16, 835 10.3390/ma16020835. 36676571 PMC9867459

[ref44] Nuckolls C.; Katz T. J.; Verbiest T.; Van Elshocht S.; Kuball H. G.; Kiesewalter S.; Lovinger A. J.; Persoons A. Circular Dichroism and UV–Visible Absorption Spectra of the Langmuir–Blodgett Films of an Aggregating Helicene. J. Am. Chem. Soc. 1998, 120, 8656–8660. 10.1021/ja981757h.

[ref45] Verbiest T.; Van Elshocht S.; Kauranen M.; Hellemans L.; Snauwaert J.; Nuckolls C.; Katz T. J.; Persoons A. Strong Enhancement of Nonlinear Optical Properties Through Supramolecular Chirality. Science 1998, 282, 913–915. 10.1126/science.282.5390.913. 9794754

[ref46] Yang C.; Zhu X.; Liu M. Helicenes at Air/Water Interface: Spreading Film and Metal Ion Induced a Helical Ring Nanostructure. Langmuir 2021, 37, 10241–10247. 10.1021/acs.langmuir.1c01810. 34379419

[ref47] Schrettl S.; Stefaniu C.; Schwieger C.; Pasche G.; Oveisi E.; Fontana Y.; Fontcuberta i Morral A.; Reguera J.; Petraglia R.; Corminboeuf C.; Brezesinski G.; Frauenrath H. Functional Carbon Nanosheets Prepared from Hexayne Amphiphile Monolayers at Room Temperature. Nat. Chem. 2014, 6, 468–476. 10.1038/nchem.1939. 24848231

[ref48] Krieg E.; Bastings M. M. C.; Besenius P.; Rybtchinski B. Supramolecular Polymers in Aqueous Media. Chem. Rev. 2016, 116, 2414–2477. 10.1021/acs.chemrev.5b00369. 26727633

[ref49] Gaines G. L. J. Insoluble Monolayers at Liquid-Gas Interfaces; Wiley-Interscience: New York, 1966.

[ref50] Grüniger H.; Möbius D.; Meyer H. Enhanced light reflection by dye monolayers at the air–water interface. J. Chem. Phys. 1983, 79, 3701–3710. 10.1063/1.446290.

[ref51] De Greef T. F. A.; Smulders M. M. J.; Wolffs M.; Schenning A. P. H. J.; Sijbesma R. P.; Meijer E. W. Supramolecular Polymerization. Chem. Rev. 2009, 109, 5687–5754. 10.1021/cr900181u. 19769364

[ref52] Fukui T.; Kawai S.; Fujinuma S.; Matsushita Y.; Yasuda T.; Sakurai T.; Seki S.; Takeuchi M.; Sugiyasu K. Control over Differentiation of a Metastable Supramolecular Assembly in One and Two Dimensions. Nat. Chem. 2017, 9, 493–499. 10.1038/nchem.2684. 28430199

[ref53] Sasaki N.; Yuan J.; Fukui T.; Takeuchi M.; Sugiyasu K. Control over the Aspect Ratio of Supramolecular Nanosheets by Molecular Design. Chem. - Eur. J. 2020, 26, 7840–7846. 10.1002/chem.202000055. 32150308

[ref54] Mabesoone M. F. J.; Palmans A. R. A.; Meijer E. W. Solute-Solvent Interactions in Modern Physical Organic Chemistry: Supramolecular Polymers as a Muse. J. Am. Chem. Soc. 2020, 142, 19781–19798. 10.1021/jacs.0c09293. 33174741 PMC7705892

[ref55] Weissman H.; Rybtchinski B. Noncovalent Self-Assembly in Aqueous Medium: Mechanistic Insights from Time-Resolved Cryogenic Electron Microscopy. Curr. Opin. Colloid Interface Sci. 2012, 17, 330–342. 10.1016/j.cocis.2012.10.001.

[ref56] Rubia-Payá C.; de Miguel G.; Martín-Romero M. T.; Giner-Casares J. J.; Camacho L. UV–Vis Reflection–Absorption Spectroscopy at air–liquid interfaces. Adv. Colloid Interface Sci. 2015, 225, 134–145. 10.1016/j.cis.2015.08.012. 26385430

[ref57] Tayi A. S.; Kaeser A.; Matsumoto M.; Aida T.; Stupp S. I. Supramolecular Ferroelectrics. Nat. Chem. 2015, 7, 281–294. 10.1038/nchem.2206. 25803466

[ref58] Dumele O.; Chen J.; Passarelli J. V.; Stupp S. I. Supramolecular Energy Materials. Adv. Mater. 2020, 32, 1907247 10.1002/adma.201907247. 32162428

[ref59] Li Y.; Zhang X.; Liu D. Recent Developments of Perylene Diimide (PDI) Supramolecular Photocatalysts: A Review. J. Photochem. Photobiol. C 2021, 48, 100436 10.1016/j.jphotochemrev.2021.100436.

