# Supplementary material for: Supramolecular Large Nanosheets Assembled at Air/Water Interfaces and in Solution from Amphiphilic Heptagon-Containing Nanographenes
Source: J Org Chem. 2023 Dec 13;89(1):163–73. doi: 10.1021/acs.joc.3c01854 (PMC10777395; doi:10.1021/acs.joc.3c01854)
Supplement: Supplementary file 1 — jo3c01854_si_001.pdf [file jo3c01854_si_001.pdf]

Supramolecular Large Nanosheets Assembled at Air/Water Interfaces and in Solution from Amphiphilic Heptagon-Containing Nanographenes

Arthur H. G. David,[†] Mari C. Mañas-Torres,[†] Marcos D. Codesal,[†] Irene López-Sicilia,[‡] María T. Martín-Romero,[‡] Luis Camacho,[‡] Juan M. Cuerva,[†] Victor Blanco,[†] Juan J. Giner-Casares,[*‡] Luis Álvarez de Cienfuegos[*†] and Araceli G. Campaña[*†]

[†] Departamento de Química Orgánica Facultad de Ciencias, Unidad de Excelencia Química Aplicada a Biomedicina y Medioambiente, Universidad de Granada Avda. Fuentenueva, s/n, 18071 Granada, Spain
[‡] Departamento de Química Física y T. Aplicada, Instituto Químico para la Energía y el Medioambiente IQUEMA, Facultad de Ciencias, Universidad de Córdoba, Campus de Rabanales, Ed. Marie Curie, E-14071 Córdoba, Spain

E-mail: jjginer@uco.es, lac@ugr.es, araceligc@ugr.es

- Supporting Information -

Table of Contents

1. Experimental Procedures S3
1.1. General Methods S3
1.2 Synthesis Overview S5
1.3 Synthetic procedures and characterization details S8
2. Additional Supporting Figures S14
3. NMR spectra of new compounds S25
3. ^{1}H NMR spectra of known compounds S37
5. HRMS spectra of final compounds S39
6. IR spectra of final compounds S42
7. UV-Vis and fluorescence spectra of final compounds S44
8. Computational methods S48
9. References S49

1. Experimental Procedures

1.1. General Methods

Unless otherwise noted, commercially available reagents, solvents and anhydrous solvents were used as purchased without further purification. Freshly distilled THF was distilled over Na/benzophenone. $PdCl_2(PPh_3)_2$,[S1] $Pd(PPh_3)_4$,[S2] **4**,[S3] **6a**[S3] and **8a**[S4] were prepared according to literature procedures.

TLC was performed on Merck Silica gel 60 F_{254} aluminum sheets. The TLC plates were stained with potassium permanganate (1% w/v in water) or cerium molybdate stain (Hanessian's stain), or observed under UV light when applicable. Flash column chromatography was performed with Silica gel 60 (VWR, 40-63 μm). Gel permeation chromatography was performed with Biobeads® SX−1 resin beads. Silica gel G preparative TLC plates (20×20cm, 1000 micron), were purchased from Silicycle.

^1H and $^{13}C\{^1H\}$ NMR spectra were recorded at room temperature on a Varian Direct Drive (400 MHz or 500 MHz), Bruker Avance III HD NanoBay (400 MHz) or Bruker Avance Neo (400 MHz or 500 MHz) spectrometers at a constant temperature of 298 K. Chemical shifts are given in ppm and referenced to the signal of the residual protiated solvent (^1H: δ=7.26 for $CDCl_3$ and δ=5.32 for CD_2Cl_2) or the ^{13}C signal of the solvents (^{13}C: δ=77.16 for $CDCl_3$ and δ=54.00 for CD_2Cl_2) or to the signal of the residual TMS (^1H: δ=0.00). Coupling constant (J) values are given in Hz. Abbreviations indicating multiplicity were used as follow: m = multiplet, p = quintet, q = quartet, t = triplet, d = doublet, s = singlet, br = broad. Structural assignments were made with additional information from gCOSY, gHSQC, and gHMBC experiments.

Electrospray (ESI) HRMS spectra were recorded on a Waters Xevo G2-XS QTOF and MALDI HRMS spectra were recorded on a Bruker Ultraflex III mass spectrometer.

IR spectra were recorded with a Perkin-Elmer Spectrum Two FTIR ATR spectrometer.

TEM images were obtained using a LIBRA 120 PLUS Carl Zeiss microscope. A drop of the sample solution was placed on a copper grid. The copper grid was washed 2 times with milli-Q water. The sample was stained with 1% uranyl acetate and, finally, dried.

In AFM, the samples were studied with a Park NX20 atomic force microscope (Park Systems) operating at 25ºC and atmospheric pressure. The sample was diluted by 60% with the solvent mixture (THF/H_2O 2:8). A drop of the resulting solution was deposited in a glass slide and spread over the entire surface. The excess sample was removed. The sample was completely dried.

The solutions for the studies at air/water interfaces were prepared at a concentration of 0.25 mM in chloroform (≥ 99.8%) purchased from Sigma-Aldrich (Germany) and used without further purification. Ultrapure water used as the subphase was produced by a Millipore Milli-Q unit and pre-treated by a Millipore reverse osmosis system (>18.2 MΩ·cm). The subphase temperature was 21 ºC. All experiments were performed on tables with vibration isolation using the antivibration system MOD-2 S (Accurion, Göttingen, Germany) in a large class 1000 clean room. Two different models of NIMA troughs (NIMA Technology, Coventry, England) were used in this work,

both provided with a Wilhelmy type dynamometric system using a strip of filter paper: a NIMA 611D with one moving barrier for the measurement of the reflection spectra, and a NIMA 601, equipped with two symmetrical barriers to record BAM images. To spread the amphiphilic molecule solutions on the balances, Hamilton syringes were used. After spreading the compounds, we waited a total of 15 minutes before starting compression to ensure complete evaporation of the chloroform. The layers of the compounds were compressed at a speed of 0.02 $nm^2 \cdot min^{-1} \cdot molecule^{-1}$.

The monolayers were transferred by Langmuir-Schaefer, i. e., by horizontal dipping at constant surface pressure. ($\pi = 25$ mN/m) onto solid substrates. The substrates were cleaned with an alkaline detergent and then rinsed several times with ultrapure water, ethanol and isopropanol. Therefore, multilayers by sequential monolayer transfer were assembled. The transfer ratio was closed to unity for all transferences. Samples for TEM were prepared by transference of one monolayer of **3** in a Formvar/Carbon film by Langmuir-Schaefer transfer at surface pressure of 25 mN/m. Fluorescence emission spectra were collected using an FLS980 (Edinburgh Instruments) photoluminescence spectrometer. Measurements were performed by using the R298P photomultiplier detector and the 450 W Xe1 xenon arc lamp. Fluorescence spectra were recorded under different excitation wavelength (λexc=250, 350 and 390 nm), and the integration time was set to 0.1 s per 2 nm increment in wavelength. Contact Angle (CA) Measurements were conducted by using an OpticalTensiometerTheta T200 device (Attension, Biolin Scientific) equipped with a high-speed camera (420 fps). The CA were measured in sessile drop method. The experiments were performed at room temperature and at open atmosphere. The drop of water was placed with no tilting. The substrate also displays a horizontal position in all cases. The needle is retracted by the automated system after placing the drop of water, but no tilted measurements were included. X-ray diffraction patterns of the transferred films were collected on a Bruker D8 Discover diffractometer operating at 40 kV and 40 mA and using Cu-Kα radiation (1.54059 Å).

UV-visible reflection spectra at normal incidence as the difference in reflectivity (ΔR) of the film-covered water surface and the bare surface were obtained with a Nanofilm Surface Analysis Spectrometer (RefSPEC^2, supplied by Accurion GmbH, Göttingen, Germany).

Images of the Langmuir films were obtained by Brewster angle microscopy (BAM) with a I-Elli2000 (Accurion GmbH) using a Nd:YAG diode laser with wavelength 532 nm and 50 mW, which can be recorded with a lateral resolution of 2 µm. The image processing procedure included a geometrical correction of the image, as well as a filtering operation to reduce interference fringes and noise.

1.2 Synthesis Overview

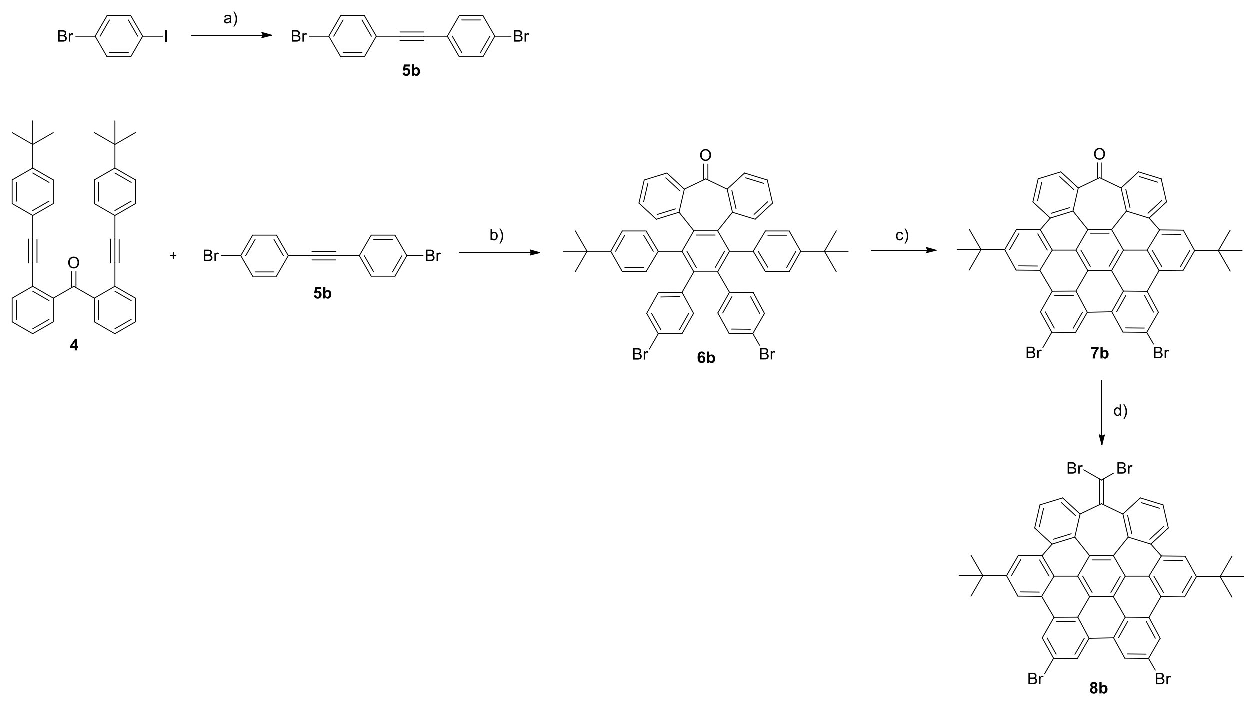

Scheme S1. Synthesis of nanographenes **7b** and **8b**: Reagents and conditions: a) trimethylsilylacetylene, $PdCl_2(PPh_3)_2$, CuI, DBU, H_2O, CH_3CN, RT, 20 h, 76%; b) $Co_2(CO)_8$, toluene, 110 ºC, 18 h, 40%; c) DDQ, CF_3SO_3H, CH_2Cl_2, 0 ºC, 10 min, 39 %; d) PPh_3, CBr_4, toluene, 120 ºC, 20 h, 84%.

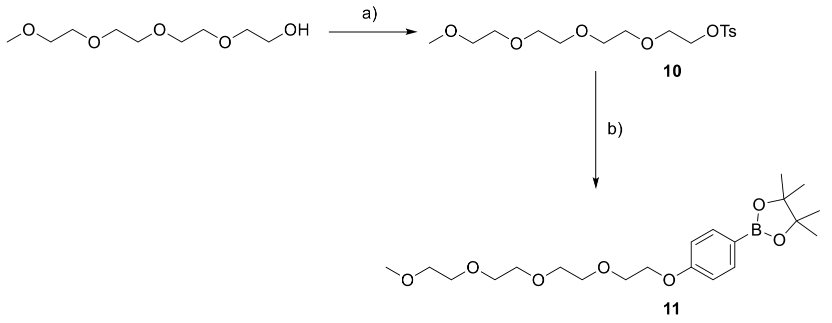

Scheme S2. Synthesis of compound **11**: Reagents and conditions: a) TsCl, Et_3N, DMAP $_{(cat)}$, CH_2Cl_2, RT, 20 h, 99%; b) 4-hydroxyphenylboronic acid pinacol ester, K_2CO_3, DMF, 80 ºC, 18 h, 82%.

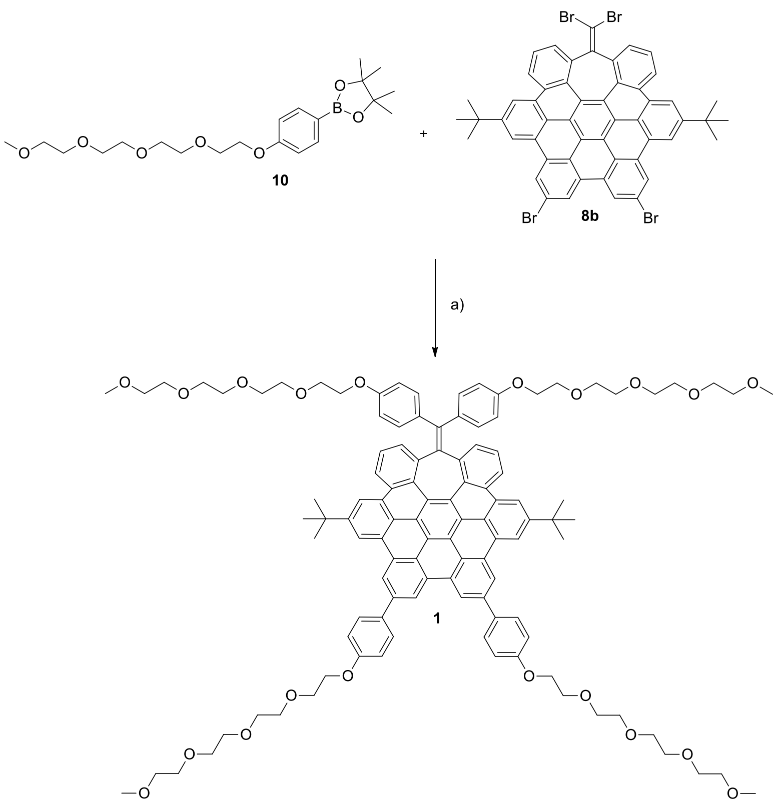

Scheme S3. Synthesis of nanographene **1**: Reagents and conditions: a) $Pd(PPh_3)_4$, K_2CO_3, EtOH, H_2O, toluene, reflux, 20 h, 13%.

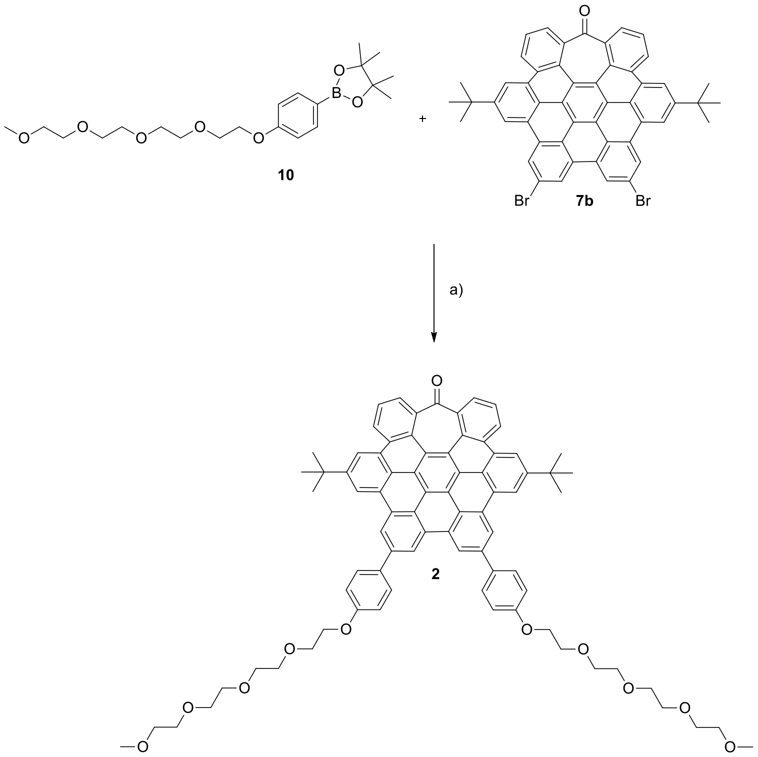

Scheme S4. Synthesis of nanographene **2**: Reagents and conditions: a) $Pd(PPh_3)_4$, K_2CO_3, EtOH, H_2O, toluene, reflux, 18 h, 54%.

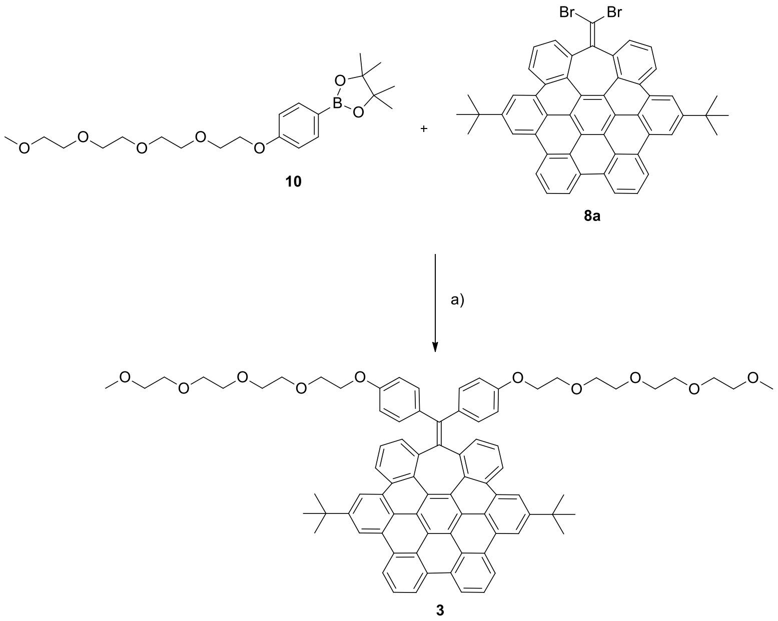

Scheme S5. Synthesis of nanographene **3**: Reagents and conditions: a) $Pd(PPh_3)_4$, K_2CO_3, EtOH, H_2O, toluene, reflux, 20 h, 28%.

1.3 Synthetic procedures and characterization details

Compound 5b:

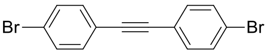

Compound **5b** was prepared according to Zhu's procedure:[S5] Under inert atmosphere, to 1-bromo-4-iodobenzene (4.00 g, 14.1 mmol), $PdCl_2(PPh_3)_2$ (989 mg, 1.41 mmol), CuI (375 mg, 1.97 mmol), were added degassed CH_3CN (40 mL), degassed DBU (12.6 mL, 84.6 mmol) and degassed H_2O (100 μL). The solution was stirred for 5 min at 70 °C heating in an oil bath. Subsequently, trimethylsilylacetylene (1.00 mL, 7.05 mmol) was added and the solution was allowed to cool to room temperature. The solution was further stirred for 20 h at room temperature and the solvent was removed under vacuum. The resulting solid was dissolved with CH_2Cl_2 (200 mL) and washed with $NH_4Cl_{(sat)}$ (2 × 200 mL). The organic layer was dried over anhydrous Na_2SO_4 and the solution was concentrated to dryness. The crude material was purified by column chromatography (SiO_2, hexane/CH_2Cl_2 80:20) to yield **5b** (1.81 g, 76%) as a white solid.

^{1}H NMR (400 MHz, $CDCl_3$): δ 7.49 (d, J = 8.2 Hz, 4H), 7.38 (d, J = 8.2 Hz, 4H). Spectral data agree with those previously reported.[S6]

Compound 6b:

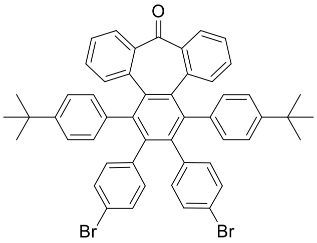

A degassed solution of **4** (346 mg, 0.699 mmol) and $Co_2(CO)_8$ (310 mg, 0.909 mmol) in anhydrous toluene (7 mL) was heated for 30 min at 110 °C in an oil bath. Subsequently, a degassed solution of **5b** (352 mg, 1.05 mmol) in anhydrous toluene (4 mL) was added portionwise for 30 min. The solution was stirred for 18 h at 110 °C, heating in an oil bath. This procedure was repeated three times. The three solutions were combined and the solvent was removed under reduced pressure. The crude material was purified by column chromatography (SiO_2, hexane/CH_2Cl_2 1:1). Fractions containing the product were combined and concentrated. The resulting solid was triturated with hexane, filtered and collected to afford **6b** (698 mg, 40%) as a brown solid.

^{1}H NMR (500 MHz, $CDCl_3$): δ 7.39 (d, J = 7.5 Hz, 2H), 7.19 (s, 4H), 7.07 (t, J = 7.5 Hz, 2H), 7.00 – 6.91 (m, 6H), 6.84 – 6.74 (m, 6H), 6.34 (d, J = 8.0 Hz, 2H), 6.26 (d, J = 8.2 Hz, 2H), 1.15 (s, 18H). $^{13}C\{^1H\}$ NMR (126 MHz, $CDCl_3$): δ 200.4, 148.9, 146.1, 141.6, 141.3, 139.3, 137.2, 136.2, 134.8, 133.11, 133.07, 132.2, 130.4, 130.2, 129.5, 128.5, 127.1, 124.4, 124.3, 123.7, 120.0, 34.4, 31.3. IR (neat): ν 2959, 1685, 1593, 1490, 1389, 1265, 1070, 1012, 931, 835 cm^{-1}. HRMS (ESI/TOF$^+$) m/z: $[M+Na]^+$ Calcd for $C_{51}H_{42}OBr_2Na$ 851.1500; Found 851.1519.

Compound 7b:

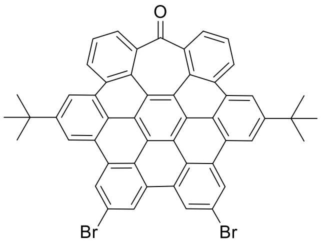

In three different flasks under Ar, to a solution of **6b** (115 mg, 0.138 mmol) and DDQ (173 mg, 0.761) in anhydrous CH_2Cl_2 (6 mL) in a water-ice bath, was added CF_3SO_3H (0.250 mL). Each solution was stirred for 10 min at 0 °C. The three solutions were combined and the solvent was evaporated under vacuum. The crude material was purified by column chromatography (SiO_2, hexane/CH_2Cl_2 50:50 to 20:80) to yield **7b** (132 mg, 39%) as a yellow solid.
^{1}H NMR (500 MHz, $CDCl_3$): δ 8.82 – 8.75 (m, 6H), 8.69 (s, 4H), 7.84 (d, *J* = 6.5 Hz, 2H), 7.74 (t, *J* = 7.7 Hz, 2H), 1.68 (s, 18H). ^{13}C{^{1}H} NMR (126 MHz, $CDCl_3$): δ 202.2, 150.3, 142.5, 132.3, 131.2, 130.5, 128.4, 128.2, 127.7, 127.1, 126.3, 125.2, 125.2, 124.41, 124.35, 123.3, 123.2, 122.9, 122.3, 121.0, 120.3, 119.1, 35.8, 32.0. IR (neat): ν 2945, 2850, 1728, 1678, 1575, 1366, 1256 cm^{-1}. HRMS (MALDI/TOF$^+$) m/z: [M]$^+$ Calcd for $C_{51}H_{32}OBr_2$: 818.0814; Found 818.0800.

Compound 8b:

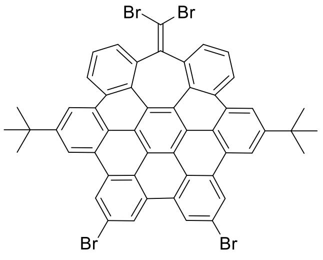

In a sealed tube under Ar, a solution of **7b** (26 mg, 0.030 mmol), PPh_3 (75 mg, 0.29 mmol) and CBr_4 (50 mg, 0.15 mmol) in anhydrous toluene (1.3 mL) was heated at 120 ºC in an oil bath for 20 h. Then, the solvent was removed and the crude material was purified by column chromatography (SiO_2, hexane/CH_2Cl_2 90:10 to 70:30) to yield **8b** (25 mg, 84%) as a yellow solid.
^{1}H NMR (500 MHz, CD_2Cl_2): δ 9.09 (d, *J* = 1.8 Hz, 2H), 9.06 (d, *J* = 1.8 Hz, 2H), 8.98 (d, *J* = 1.7 Hz, 2H), 8.95 (d, *J* = 7.5 Hz, 2H), 8.82 (d, *J* = 1.7 Hz, 2H), 7.98 (t, *J* = 7.4 Hz, 2H), 7.78 (dd, *J* = 7.3, 1.3 Hz, 2H), 1.67 (s, 18H). ^{13}C{^{1}H} NMR (126 MHz, CD_2Cl_2): δ 151.2, 147.3, 141.4, 133.1, 132.4, 131.2, 129.1, 128.8, 128.7, 128.2, 126.0, 125.5, 125.3, 124.6, 124.3, 124.0, 123.4, 123.1, 122.7, 121.6, 120.6, 119.8, 91.0, 36.2, 32.1. IR (neat): ν 2959, 2922, 2852, 1575, 1463, 1367, 1259 cm^{-1}. HRMS (MALDI/TOF$^+$) m/z: [M]$^+$ Calcd for $C_{52}H_{32}Br_4$ 971.9232; Found 971.9226.

Compound 10:

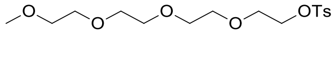

To a solution of tetraethylene glycol monomethyl ether (537 mg, 2.58 mmol) in anhydrous CH_2Cl_2 (20 mL) were added Et_3N (1.4 mL, 10 mmol), TsCl (983 mg, 5.16 mmol), and a catalytic amount of DMAP. The mixture was stirred for 20 h at room temperature. Subsequently, the solution was concentrated to dryness and the crude material was purified by column chromatography (SiO_2, hexane/EtOAc 20:80) to give **10** (921 mg, 99%) as a colorless oil. ^{1}H NMR (400 MHz, $CDCl_3$): δ 7.79 (d, *J* = 7.7 Hz, 2H), 7.33 (d, *J* = 7.9 Hz, 2H), 4.16 (m, 2H), 3.70 – 3.51 (m, 14H), 3.37 (s, 3H), 2.44 (s, 3H). Spectral data agree with those previously reported.[S7]

Compound 11:

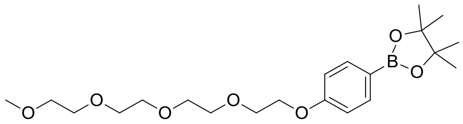

Compound **11** was prepared according to Iovine's procedure:[S8] Under an inert atmosphere, to a solution of **10** (499 mg, 1.38 mmol) and 4-hydroxyphenylboronic acid pinacol ester (302 mg, 1.38 mmol) in anhydrous DMF (8 mL) was added K_2CO_3 (190 mg, 1.38 mmol). The suspension was stirred for 18 h, heating at 80 ºC in an oil bath, and was diluted with CH_2Cl_2 (100 mL) and H_2O (80 mL). Layers were separated and the aqueous one was extracted with CH_2Cl_2 (2 × 100 mL). The combined organic layers were dried over anhydrous Na_2SO_4 and the solution was concentrated to dryness. The crude material was purified by column chromatography (SiO_2, hexane/EtOAc 20:80) to afford **11** (464 mg, 82%) as a colorless oil.

^{1}H NMR (400 MHz, $CDCl_3$): δ 7.72 (d, *J* = 8.1 Hz, 2H), 6.89 (d, *J* = 8.1 Hz, 2H), 4.14 (t, *J* = 4.9 Hz, 2H), 3.85 (t, *J* = 5.0 Hz, 2H), 3.73 – 3.61 (m, 10H), 3.53 (m, 2H), 3.36 (s, 3H), 1.32 (s, 12H). Spectral data agree with those previously reported.[S8]

Compound 1:

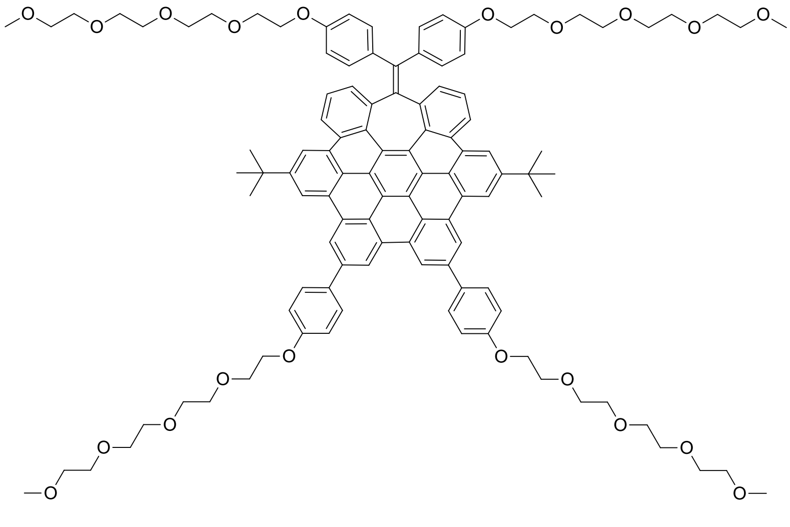

To a degassed solution of **8b** (17 mg, 0.017 mmol) and **10** (85 mg, 0.21 mmol) in toluene (5 mL) were added $Pd(PPh_3)_4$ (16 mg, 0.014 mmol), K_2CO_3 (29 mg, 0.21 mmol) and a degassed mixture of EtOH/H_2O (1:1, 1 mL). The mixture was refluxed for 20 h, heating in an oil bath, and was diluted with H_2O (50 mL). The aqueous layer was extracted with CH_2Cl_2 (3 × 30 mL). The combined organic layers were dried over anhydrous Na_2SO_4 and the solvent was evaporated under vacuum. The crude material was purified by column chromatography (SiO_2,

CH_2Cl_2/MeOH 96:4) followed by two consecutive preparative TLC (firstly, SiO_2, CH_2Cl_2/MeOH 96:4; then SiO_2, EtOAc/MeOH 92:8) to give **1** (4 mg, 13%) as a yellow solid.

^{1}H NMR (500 MHz, $CDCl_3$): δ 9.37 (s, 2H), 9.12 (s, 2H), 8.95 (s, 2H), 8.88 (s, 2H), 8.68 (d, *J* = 7.5 Hz, 2H), 8.02 (d, *J* = 8.5 Hz, 4H), 7.68 (t, *J* = 7.6 Hz, 2H), 7.57 (d, *J* = 7.0 Hz, 2H), 7.25 (d, *J* = 8.5 Hz, 4H), 6.51 (d, *J* = 8.7 Hz, 4H), 6.31 (d, *J* = 8.6 Hz, 4H), 4.31 (t, *J* = 4.8 Hz, 4H), 3.98 (t, *J* = 4.9 Hz, 4H), 3.83 – 3.54 (m, 52H), 3.47 (m, 4H), 3.39 (s, 6H), 3.31 (s, 6H), 1.69 (s, 18H). $^{13}C\{^1H\}$ NMR (126 MHz, $CDCl_3$): δ 159.0, 156.9, 149.9, 142.6, 142.1, 140.2, 139.9, 138.9, 134.9, 133.5, 131.6, 131.3, 130.7, 130.3, 130.01, 129.99, 129.4, 128.9, 127.7, 126.0, 125.5, 124.3, 123.9, 122.9, 121.1, 120.6, 120.5, 120.4, 118.4, 115.5, 113.6, 72.1, 72.0, 71.1, 70.9, 70.84, 70.82, 70.72, 70.69, 70.6, 70.0, 69.7, 67.9, 67.1, 59.2, 59.1, 35.8, 32.0. IR (neat): ν 2922, 2870, 1727, 1606, 1510, 1454, 1372, 1246, 1110, 830 cm^{-1}. HRMS (MALDI/TOF$^+$) m/z: $[M+Na]^+$ Calcd for $C_{112}H_{124}O_{20}Na$ 1811.8578, Found 1811.8590.

Compound 2:

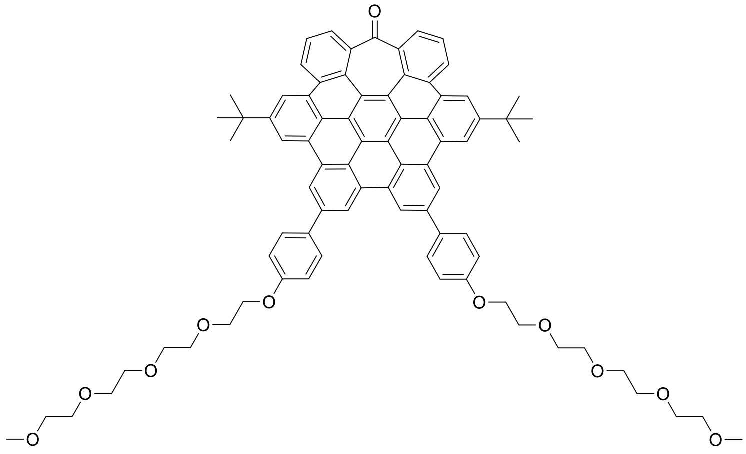

To a degassed solution of **7b** (21 mg, 0.024 mmol) and **10** (60 mg, 0.15 mmol) in toluene (5 mL) were added $Pd(PPh_3)_4$ (14 mg, 0.012 mmol), K_2CO_3 (40 mg, 0.29 mmol) and a degassed mixture of EtOH/H_2O (1:1, 1 mL). The mixture was refluxed for 18 h, heating in an oil bath, and was diluted with H_2O (30 mL). The aqueous layer was extracted with CH_2Cl_2 (3 × 30 mL). The combined organic layers were dried over anhydrous Na_2SO_4 and the solvent was removed under reduced pressure. The crude material was purified by column chromatography (SiO_2, CH_2Cl_2/MeOH 96:4) followed by gel permeation chromatography (Bio-Beads® SX─1, CH_2Cl_2) and a preparative TLC (SiO_2, EtOAc) to afford **2** (16 mg, 54%) as a yellow solid.

^{1}H NMR (500 MHz, $CDCl_3$): δ 8.79 (d, *J* = 8.1 Hz, 2H), 8.69 (s, 2H), 8.61 (s, 2H), 8.43 (s, 2H), 8.01 – 7.90 (m, 4H), 7.82 (t, *J* = 7.6 Hz, 2H), 7.13 (d, *J* = 7.9 Hz, 4H), 6.71 (d, *J* = 8.0 Hz, 4H), 4.16 (t, *J* = 4.7 Hz, 4H), 3.99 (t, *J* = 4.4 Hz, 4H), 3.90 – 3.66 (m, 20H), 3.57 (m, 4H), 3.39 (s, 6H), 1.65 (s, 18H). $^{13}C\{^1H\}$ NMR (126 MHz, $CDCl_3$): δ 203.4, 158.0, 149.6, 142.7, 138.8, 133.2, 131.3, 130.4, 129.8, 129.7, 128.3, 128.1, 127.3, 126.2, 124.3, 123.6, 123.4, 123.1, 122.9, 120.8, 120.4, 120.3, 119.0, 118.1, 114.6, 72.1, 71.1, 71.0, 70.88, 70.86, 70.7, 70.0, 67.4, 59.2, 35.7, 32.1. IR (neat): ν 2955, 2889, 1682, 1608, 1514, 1455, 1371, 1247, 1115, 828 cm^{-1}. HRMS (ESI/TOF$^+$) m/z: $[M+Na]^+$ Calcd for $C_{81}H_{78}O_{11}Na$: 1249.5442; Found 1249.5483.

Compound 3:

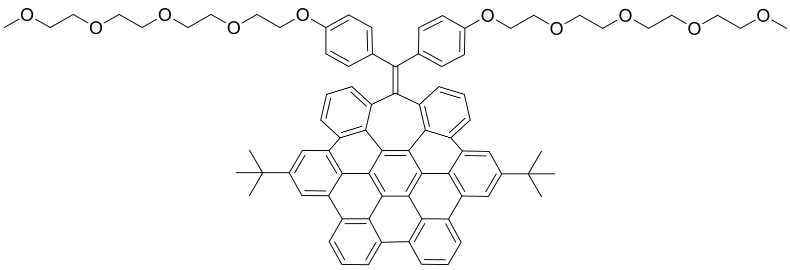

To a degassed solution of **8a** (64 mg, 0.078 mmol) and **10** (206 mg, 0.502 mmol) in toluene (15 mL) were added $Pd(PPh_3)_4$ (45 mg, 0.039 mmol), K_2CO_3 (130 mg, 0.941 mmol) and a degassed mixture of EtOH/H_2O (1:1, 3 mL). The mixture was refluxed for 20 h, heating in an oil bath, and was diluted with H_2O (50 mL). The aqueous layer was extracted with CH_2Cl_2 (3 × 50 mL). The combined organic layers were dried over anhydrous Na_2SO_4 and the solvent was evaporated under vacuum. The crude material was purified by column chromatography (SiO_2, CH_2Cl_2/MeOH 98:2) followed by two consecutive preparative TLC (firstly, SiO_2, CH_2Cl_2/MeOH 98:2; then SiO_2, EtOAc/MeOH 95:5) to yield **3** (27 mg, 28%) as a yellow solid.

^{1}H NMR (500 MHz, $CDCl_3$): δ 9.13 (d, *J* = 7.6 Hz, 2H), 8.98 (d, *J* = 7.2 Hz, 2H), 8.87 (m, 4H), 8.68 (m, 2H), 8.17 (t, *J* = 7.8 Hz, 2H), 7.69 (t, *J* = 7.3 Hz, 2H), 7.57 (dd, *J* = 7.2, 1.3 Hz, 2H), 6.50 (d, *J* = 8.7 Hz, 4H), 6.31 (d, *J* = 8.7 Hz, 4H), 3.80 (m, 4H), 3.64 (m, 4H), 3.60 – 3.55 (m, 20H), 3.47 (m, 4H), 3.31 (s, 6H), 1.67 (s, 18H). ^{13}C{^{1}H} NMR (126 MHz, $CDCl_3$): δ 156.8, 149.8, 142.6, 140.1, 138.8, 133.5, 131.3, 131.0, 130.3, 130.2, 129.9, 128.8, 127.7, 127.2, 126.0, 125.4, 125.3, 123.7, 122.9, 122.2, 121.7, 121.0, 120.7, 120.6, 118.2, 113.6, 72.0, 70.8, 70.7, 70.6, 69.7, 67.1, 59.1, 35.8, 32.0. IR (neat): ν 2952, 2869, 1605, 1508, 1454, 1369, 1244, 1107, 926, 823 cm^{-1}. HRMS (ESI/TOF$^+$) m/z: $[M+Na]^+$ Calcd for $C_{82}H_{80}O_{10}Na$: 1247.5649, Found 1247.5627.

PREPARATION OF THE SUPRAMOLECULAR AGGREGATES

Firstly, we tried to promote the self-assembly in pure organic solvents, but after testing some of the most common organic solvents (hexane, cyclohexane, toluene, dioxane, THF), the monomers, in all the cases, remained in solution. Then, we decided to introduce water in different proportions to force the aggregation. At this respect, we selected different organic solvents able to mix with water, such as methanol, ethanol and THF. Of all the solvents and solvent mixtures, only the combination THF/H_2O in the proportions reported in the manuscript (3:7, 2:8 and 1:9) produced stable whitish suspensions. Therefore, we decided to explore these solutions. Additionally, different suspensions in mixtures of organic solvents/water were also heated to promote solution and explored aggregation after cooling. These experiments did not improve the results observed at room temperature. Finally, we incubated the suspensions for different time and, in this case, we could observe a higher number of nanosheets versus nanospheres in TEM samples.

Final protocol leading to formation of nanosheets:

In a vial, the corresponding nanographene was dissolved in THF (200 µL), then, H_2O (800 µL) was added slowly and gently (addition time: 10 s) on the walls of the vial using a micropipette without stirring (final concentration

ca. 2×10^{-4} M). After the addition of H_2O, two phases were observed, the mixture was not shaken or stirred, and mixed slowly upon time. The resulting mixture was incubated for one day to one week at room temperature.

2. Additional Supporting Figures

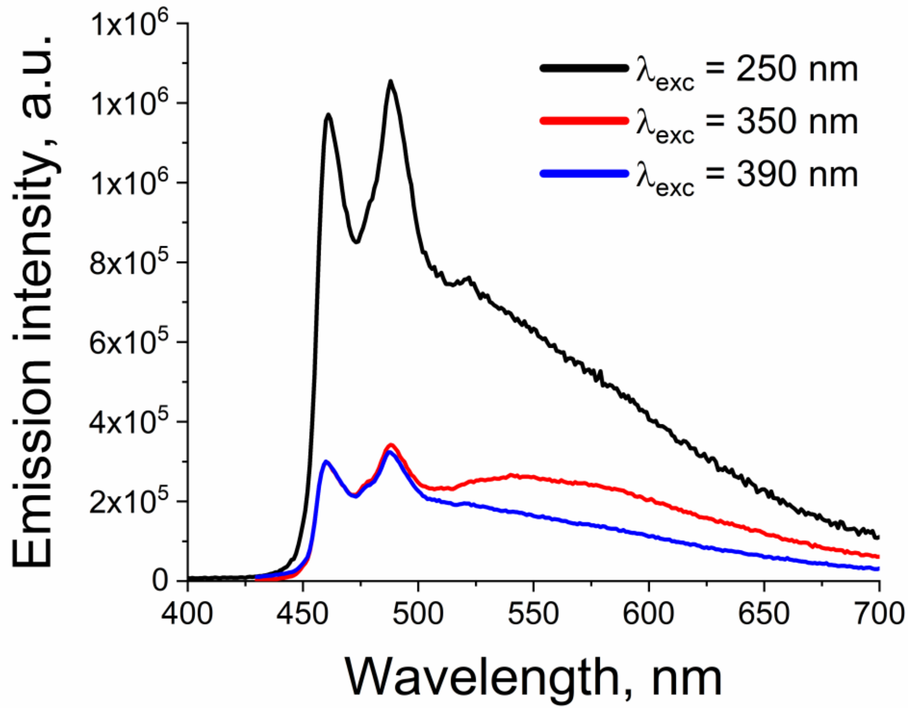

Figure S1. Fluorescence measurements for 8 films of **3** transferred from the air/water interface to quartz substrates. Wavelength of excitation is included in the inset.

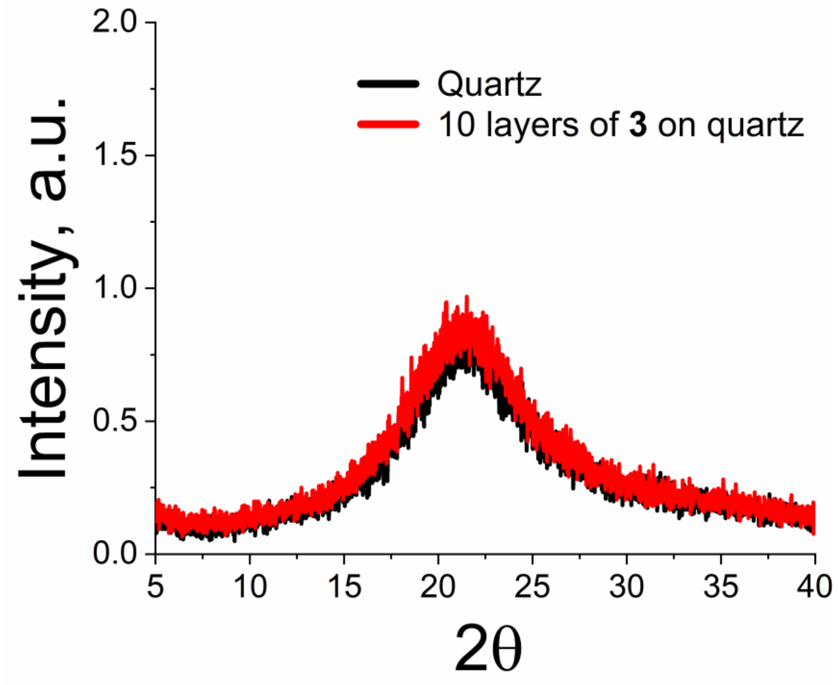

Figure S2. X-ray diffractogram for bare quartz substrate (black line) and 10 layers of **3** transferred from the air/water interface on the quartz substrate (red line).

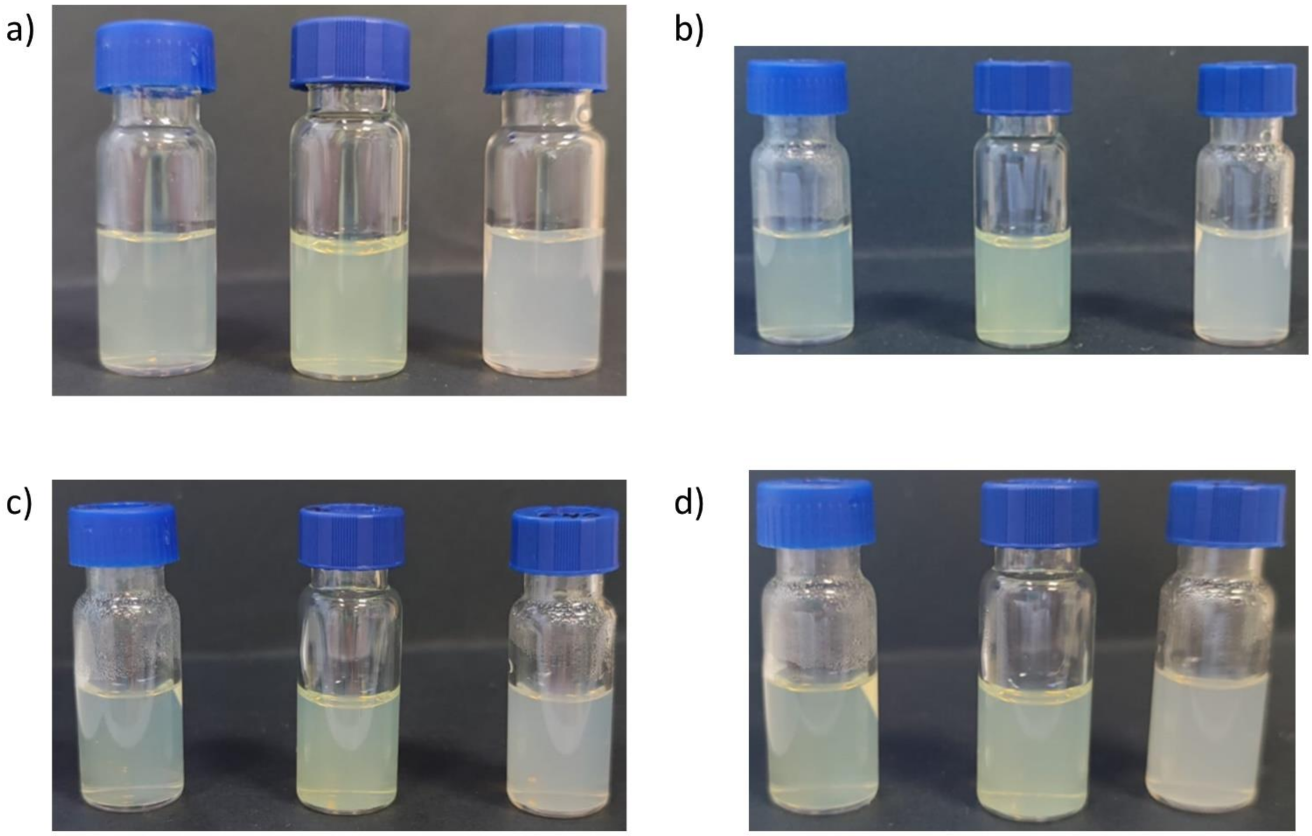

Figure S3. Photographs of the cloudy suspensions obtained from the incubation of compounds **1** (middle), **2** (left) and **3** (right) in THF/H_2O (20:80) for: a) 0 h; b) 12 h; c) 24 h; d) 3 d.

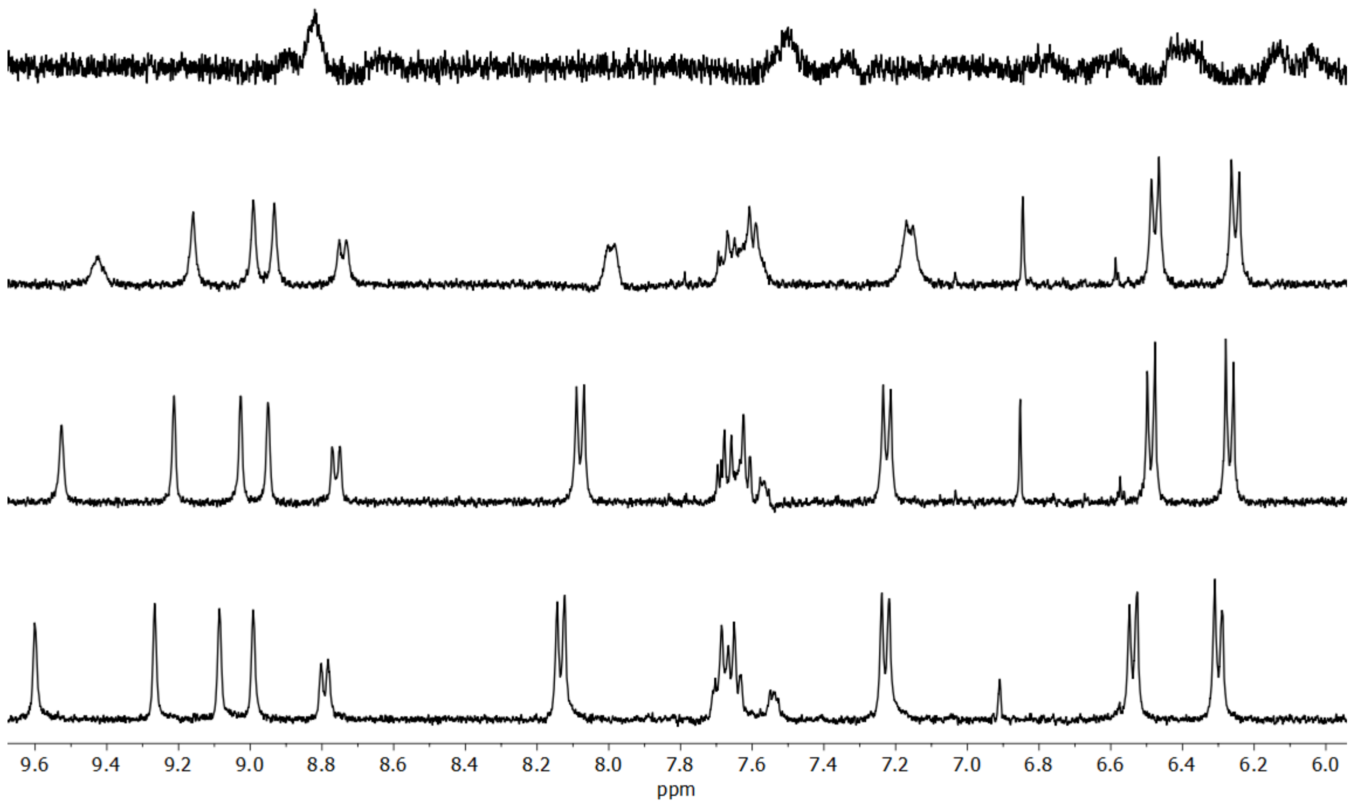

Figure S4. Partial ^{1}H NMR (400 MHz, THF-d_8/D_2O) spectrum of **1** (2.5 mM) at different THF/H_2O ratios (100:0, 80:20, 60:40, 40:60 from bottom to top).

Since the shift of the residual protiated solvent signal in THF-d_8 is dependent on the ratio of the solvent mixture, its chemical shift was corrected running a blank experiment for each mixture using DMF as reference. The chemical shift signal of H atom attached to the carbonyl group does not change significantly between THF-d_8 (δ = 7.91 ppm) and D_2O (δ = 7.92 ppm).[S9] Therefore, we recorded the ^{1}H NMR spectra of DMF at the same concentration in THF-d_8/D_2O 100:0, 80:20, 60:40 and 40:60 mixtures and referenced them with the formamide ^{1}H signal. The chemical shift for the residual protiated solvent signals in THF-d_8 were measured for each solvent mixture and corrected accordingly in the ^{1}H NMR spectra of the different compounds.

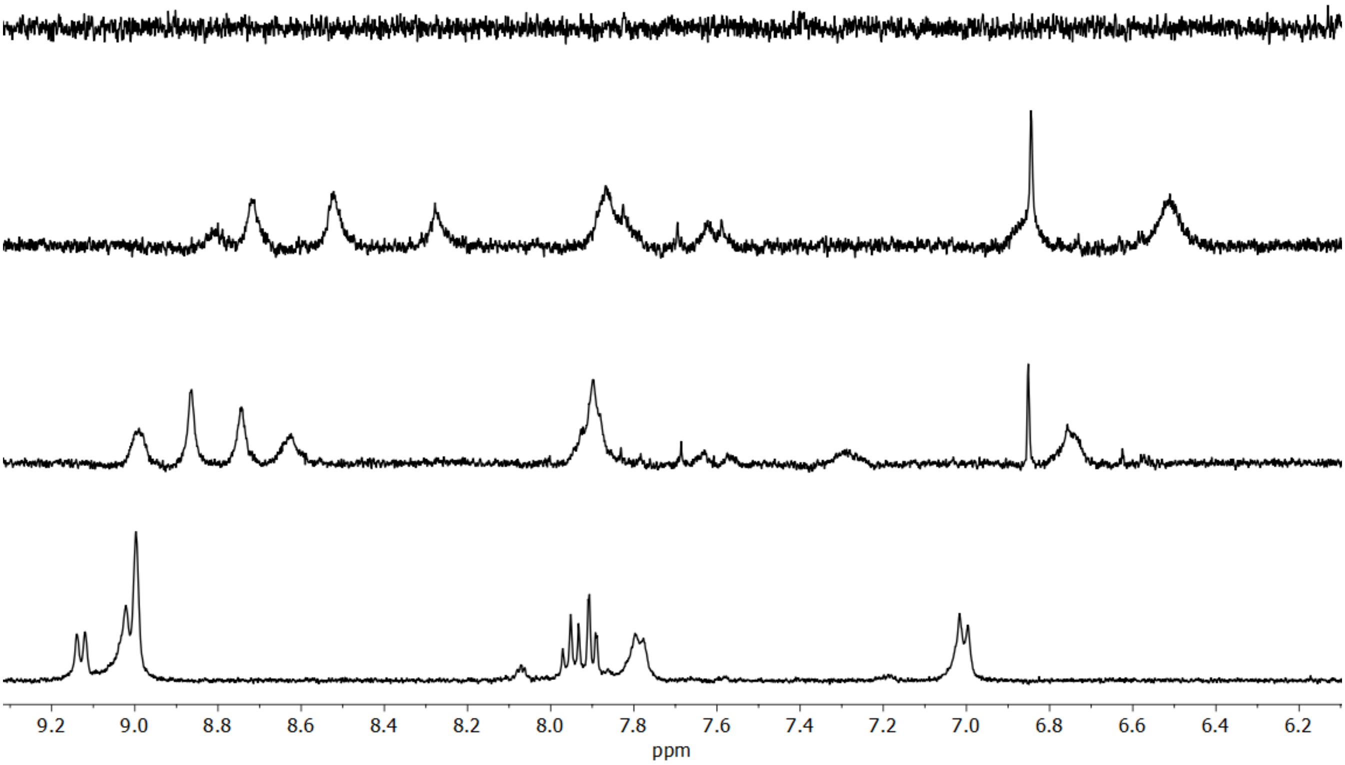

Figure S5. Partial ^{1}H NMR (400 MHz, THF-d_8/D_2O) spectrum of **2** (2.5 mM) at different THF/H_2O ratios (100:0, 80:20, 60:40, 40:60 from bottom to top).

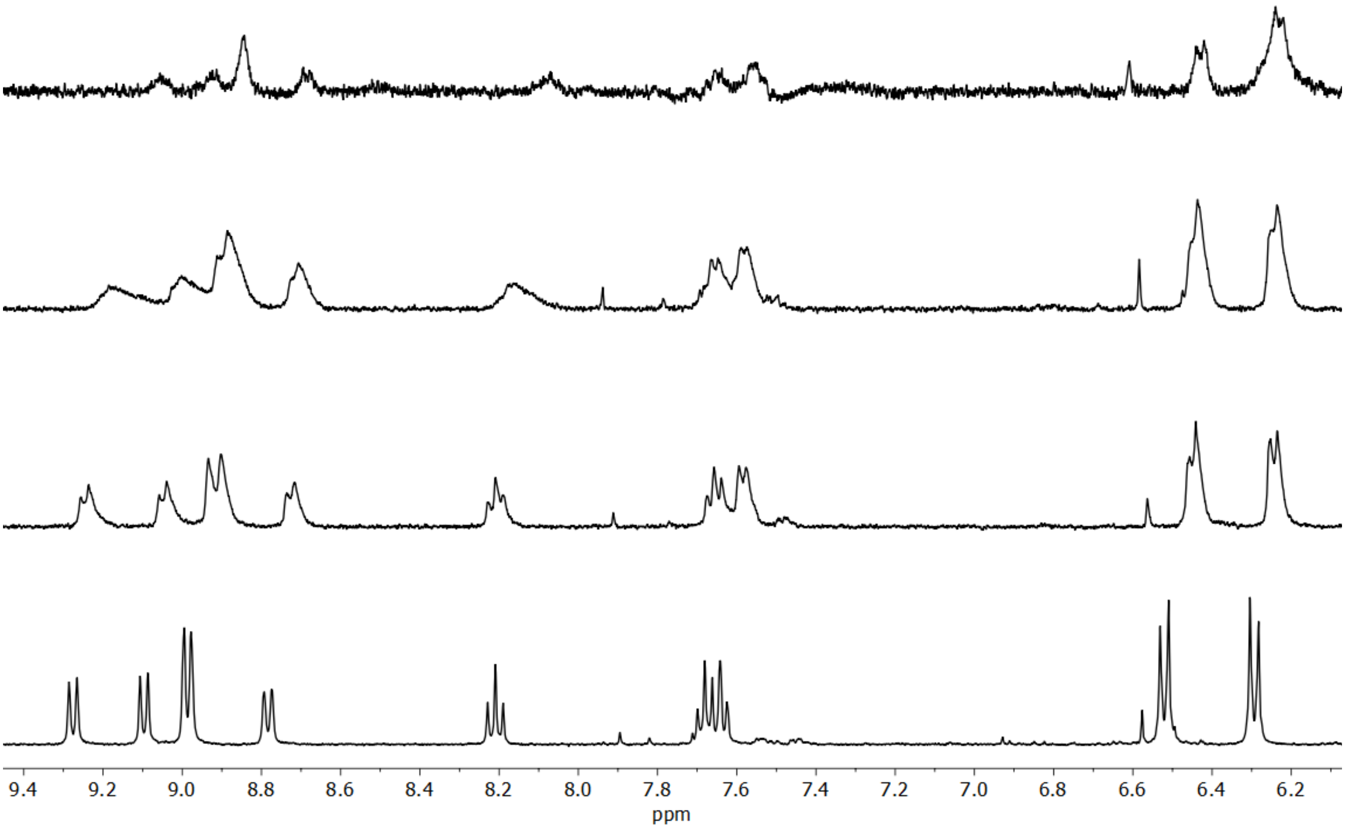

Figure S6. Partial ^{1}H NMR (400 MHz, THF-d_8/D_2O) spectrum of **3** (2.5 mM) at different THF/H_2O ratios (100:0, 80:20, 60:40, 40:60 from bottom to top).

The effect of aggregation on the optical properties was evaluated on nanographenes **1-3** using different mixtures of THF/H_2O. UV-vis spectra of **1-3** (*ca.* 1 × 10^{-4} M, 0.2 cm cuvette) exhibit a decrease of the intensity and a broadening of the 300-450 nm band upon increasing the H_2O ratio. Quenching of the luminescence band between 400-600 nm also occurs upon increasing the H_2O ratio.

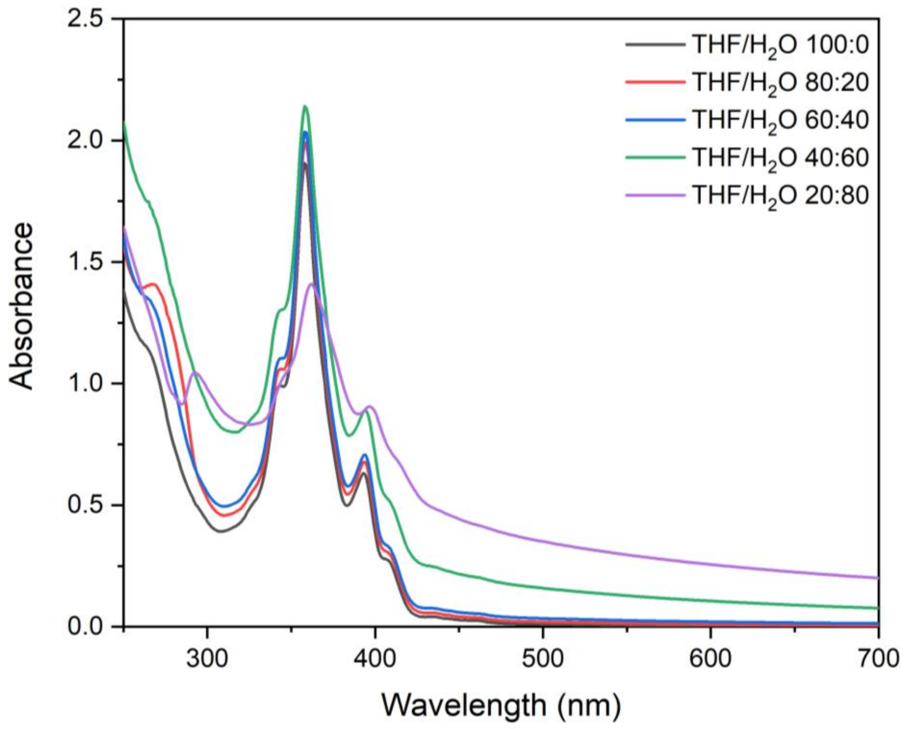

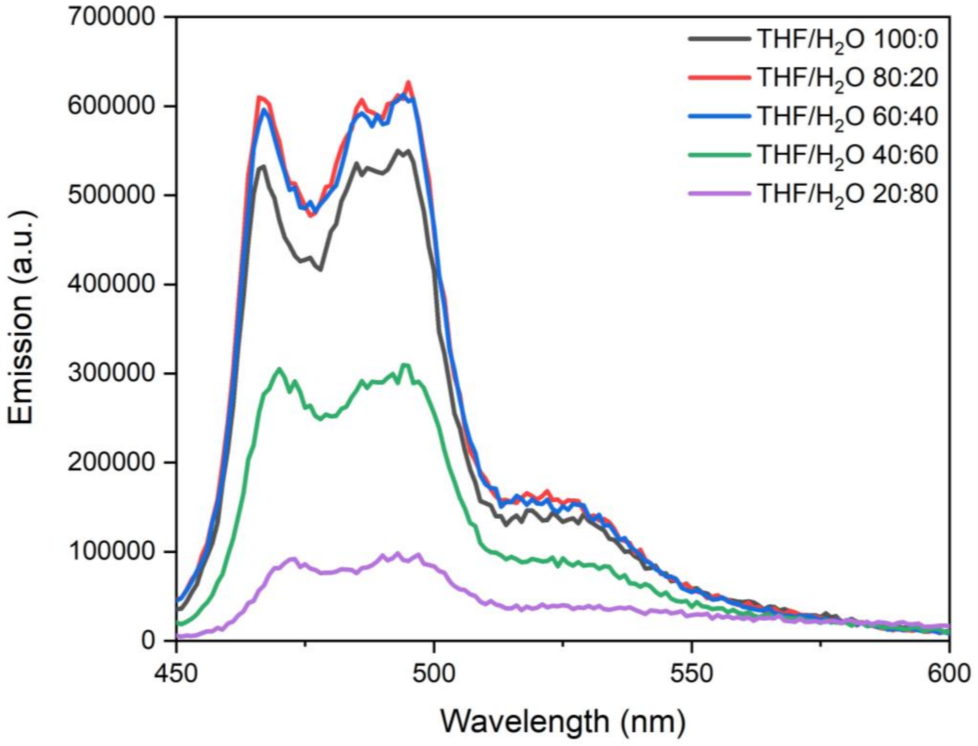

Figure S7. UV-Vis (top) and emission (bottom) spectra of **1** (*ca.* 1 × 10^{-4} M) in THF/H_2O mixtures with different ratio.

Figure S8. UV-Vis (top) and emission (bottom) spectra of **2** (*ca.* 1×10^{-4} M) in THF/H_2O mixtures with different ratio.

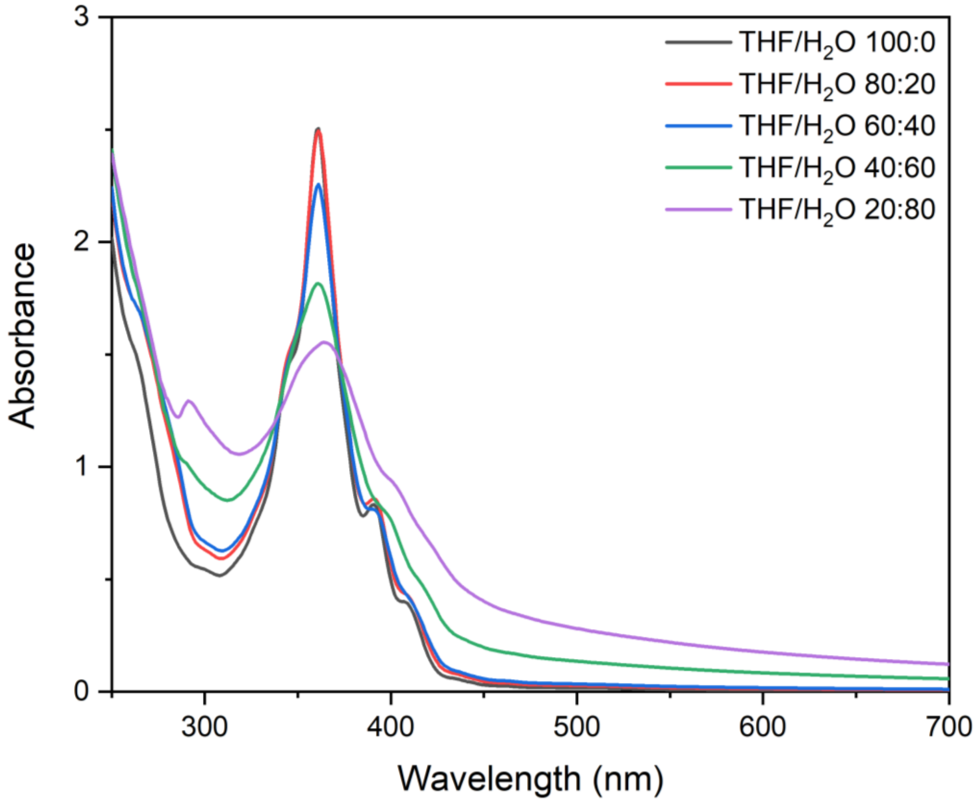

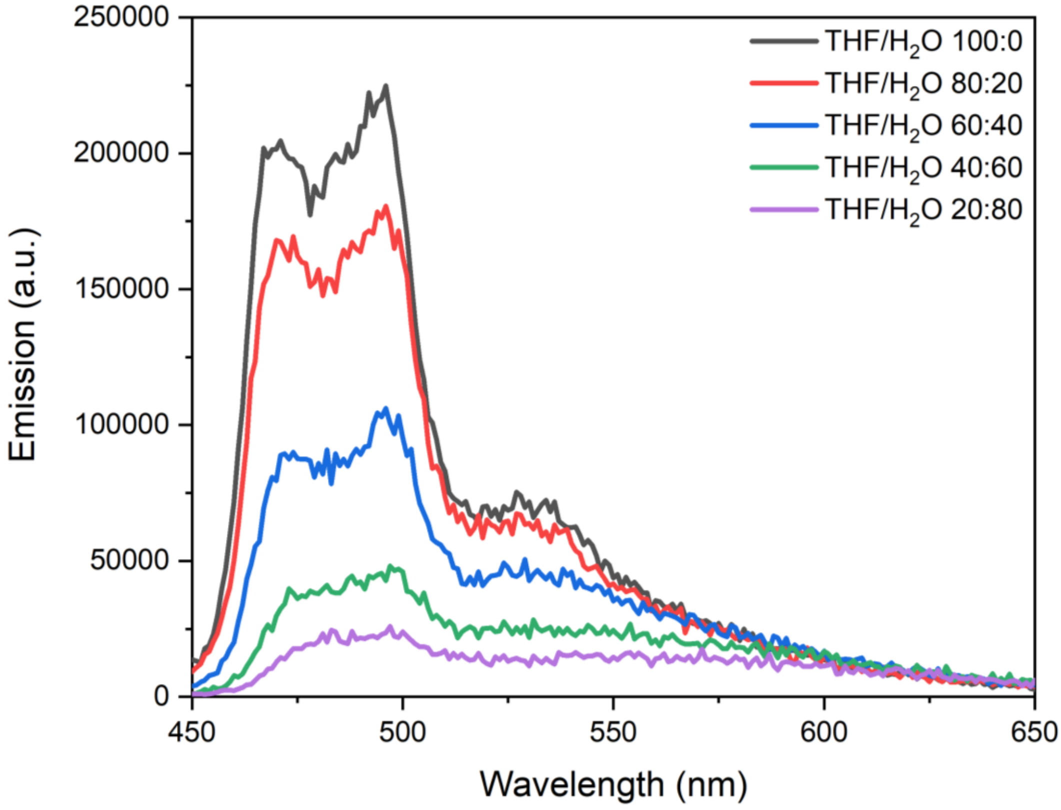

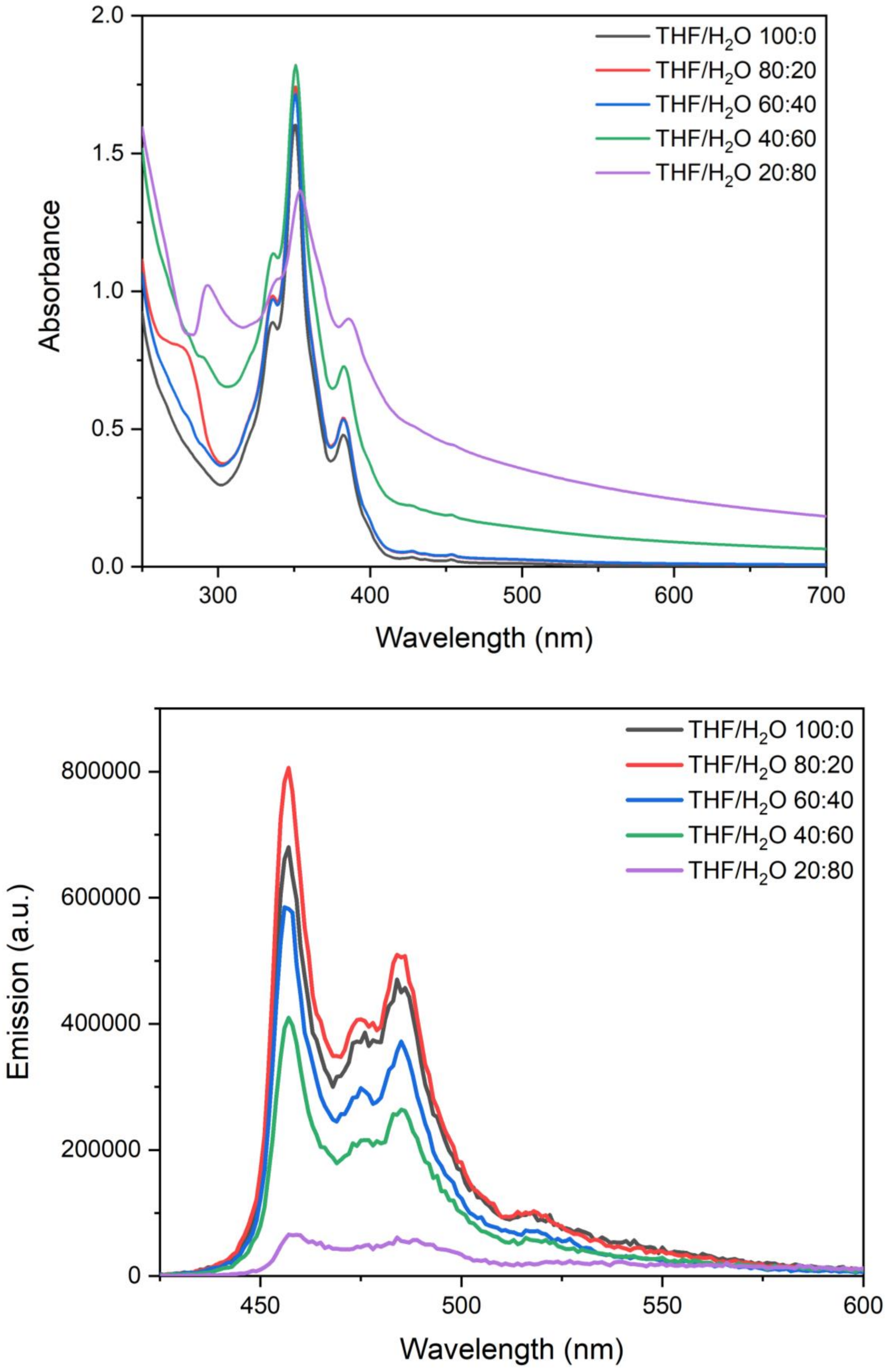

Figure S9. UV-Vis (top) and emission (bottom) spectra of **3** (*ca.* 1×10^{-4} M) in THF/H_2O mixtures with different ratio.

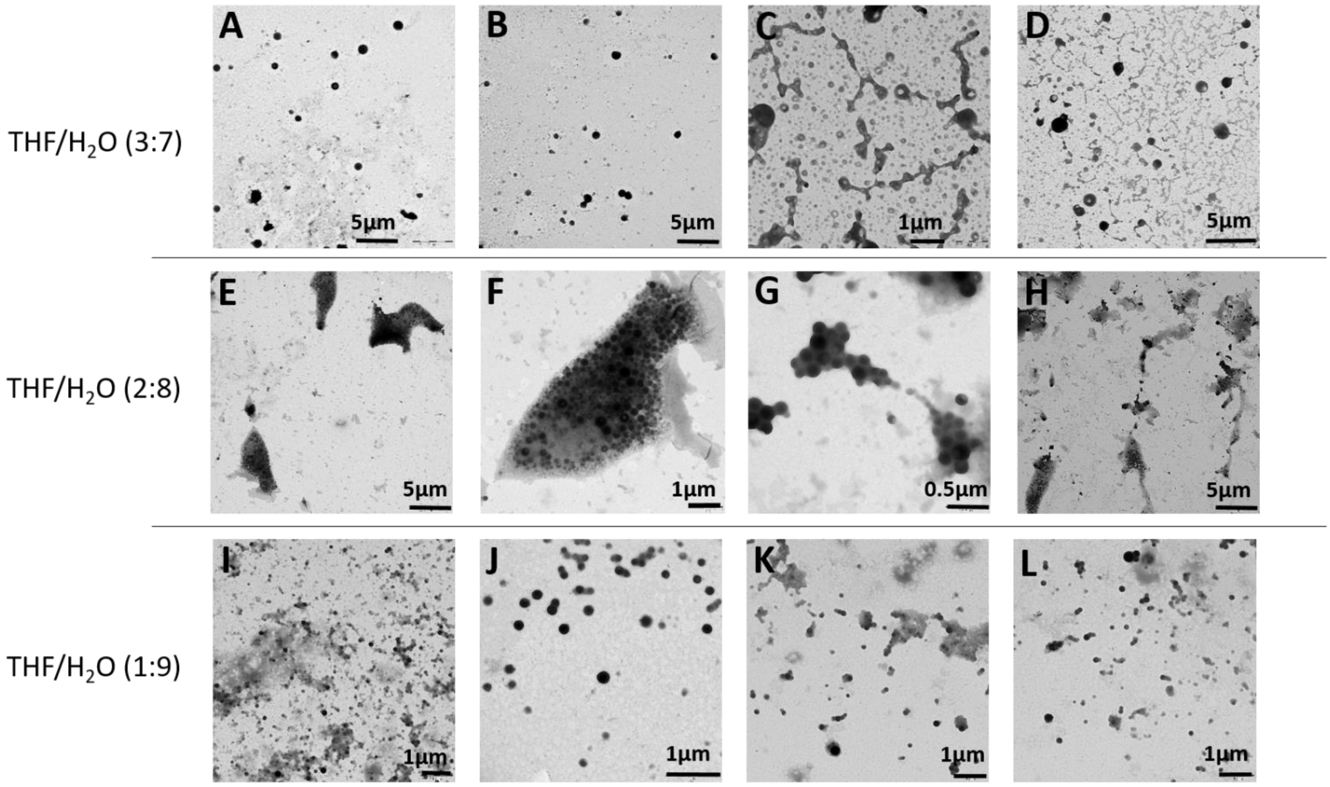

Figure S10. TEM pictures taken after incubation of compound **1** for 24 h in THF/H_2O mixtures of different proportions as indicated in the image.

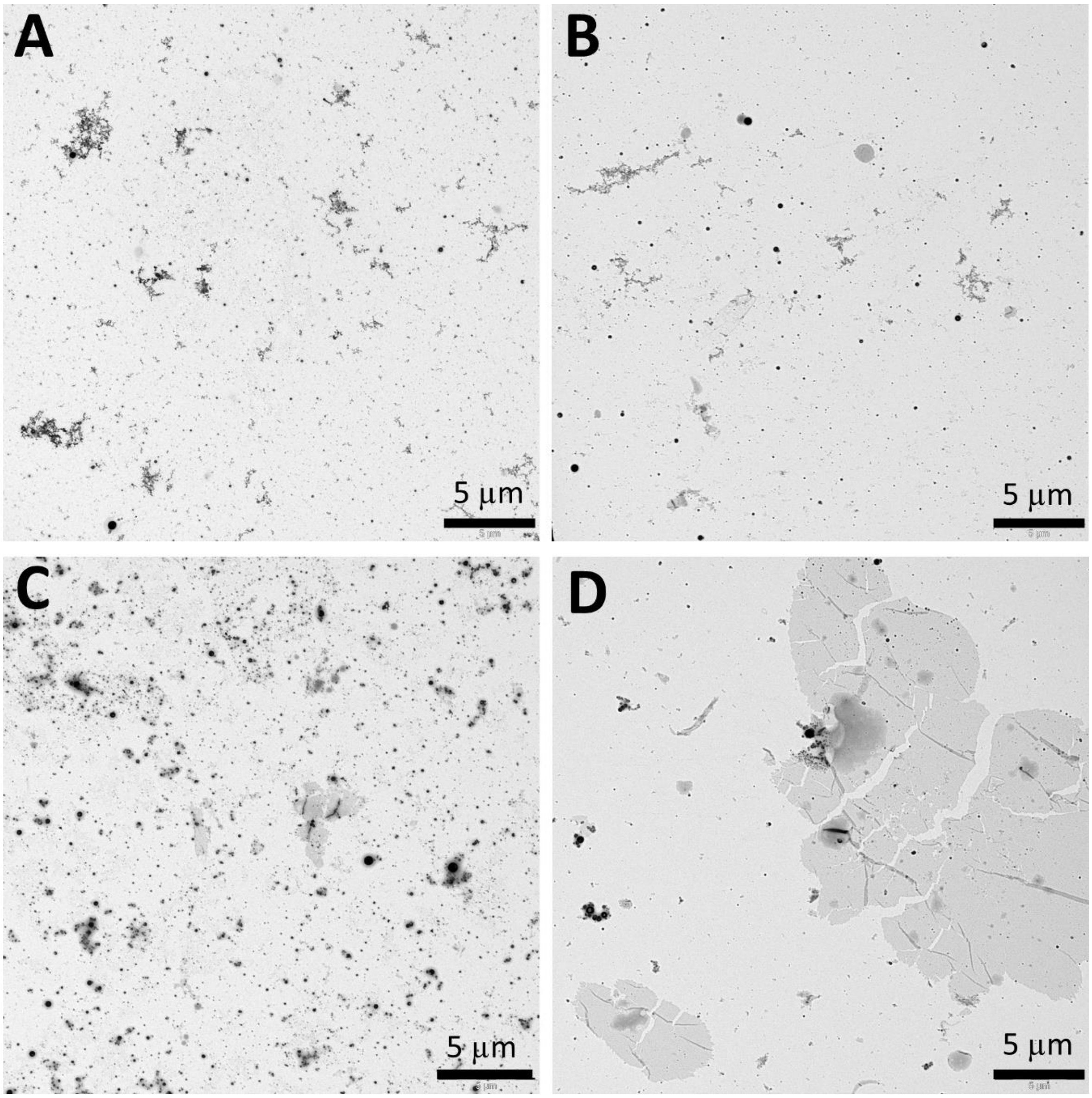

Figure S11. TEM pictures taken after incubation of compound **1** in THF/H_2O 20:80 for: A) 24 h; B) 4 d; C) 6 d; D) 11 d.

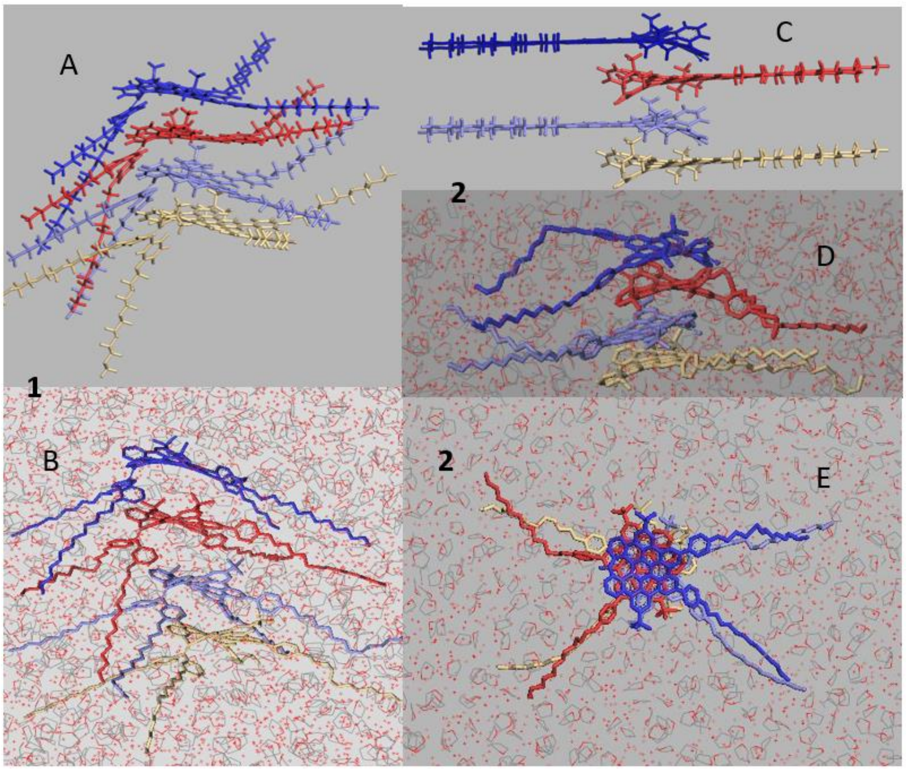

Figure S12. Representative images from the MD simulation for compounds **1** (A and B) and **2** (C-E). For a better visualization, the solvent molecules are not included in A and C.

3. NMR spectra of new compounds

Compound 6b:

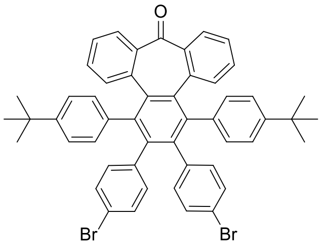

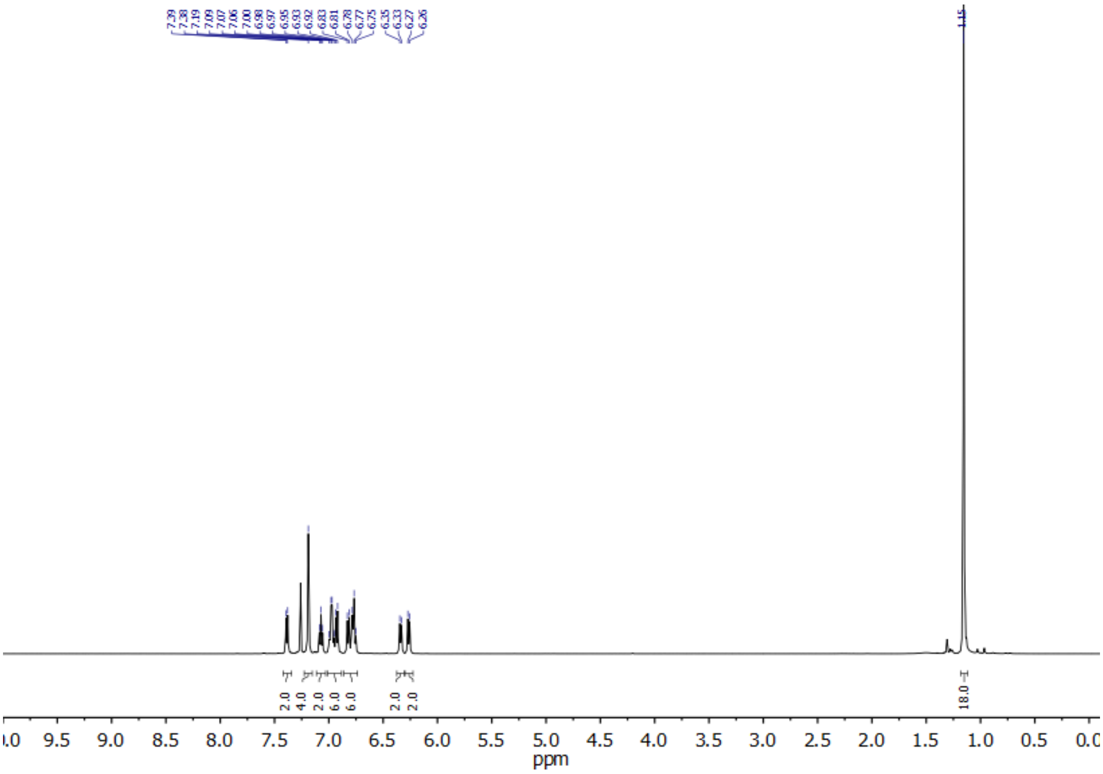

Figure S13. ^{1}H NMR (500 MHz, $CDCl_3$) spectrum of **6b**.

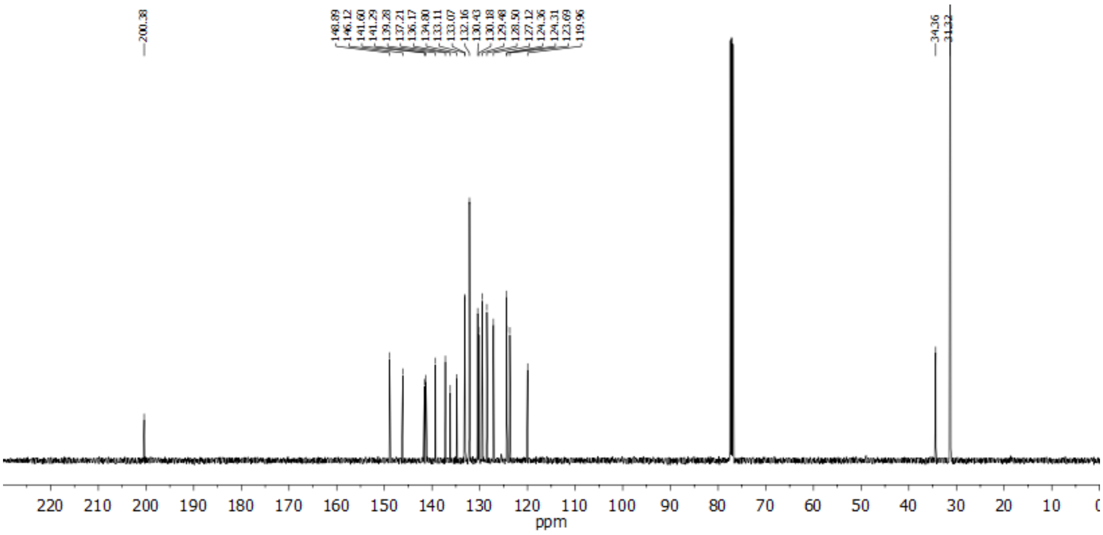

Figure S14. $^{13}C\{^{1}H\}$ NMR (126 MHz, $CDCl_3$) spectrum of **6b**.

Compound 7b:

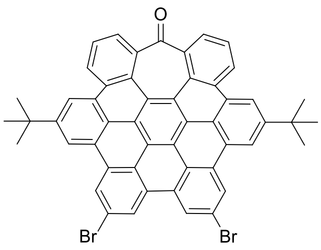

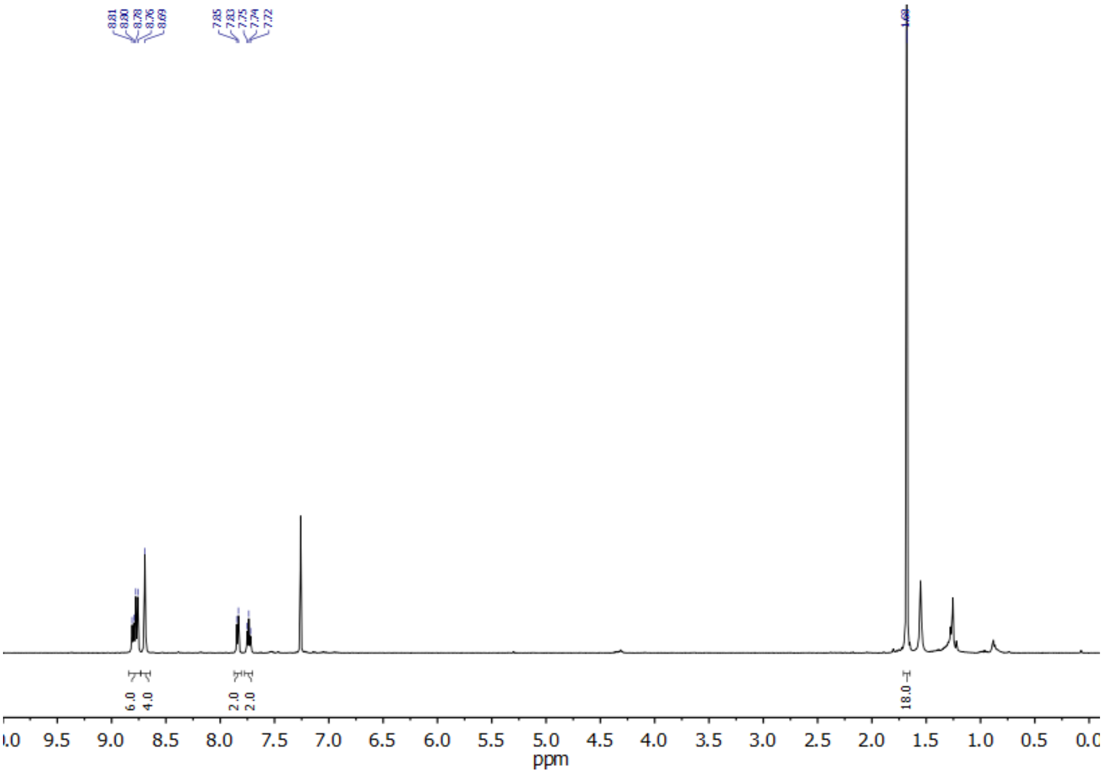

Figure S15. ^{1}H NMR (500 MHz, $CDCl_3$) spectrum of **7b**.

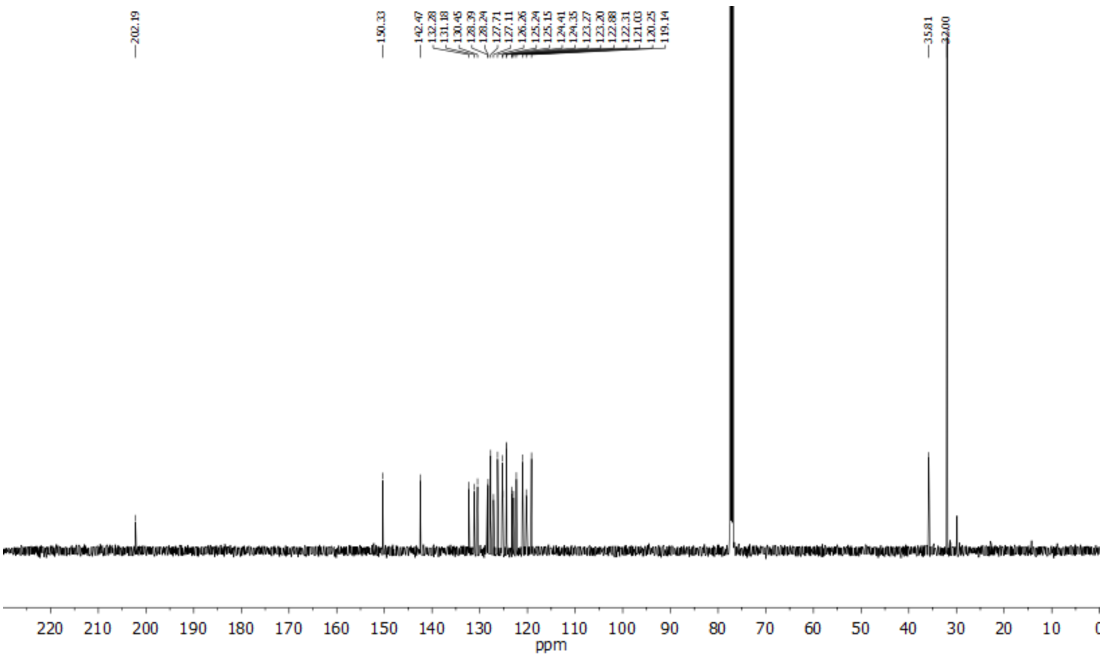

Figure S16. ^{13}C{^{1}H} NMR (126 MHz, $CDCl_3$) spectrum of **7b**.

Compound 8b:

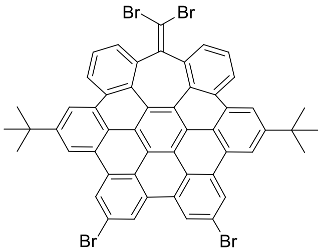

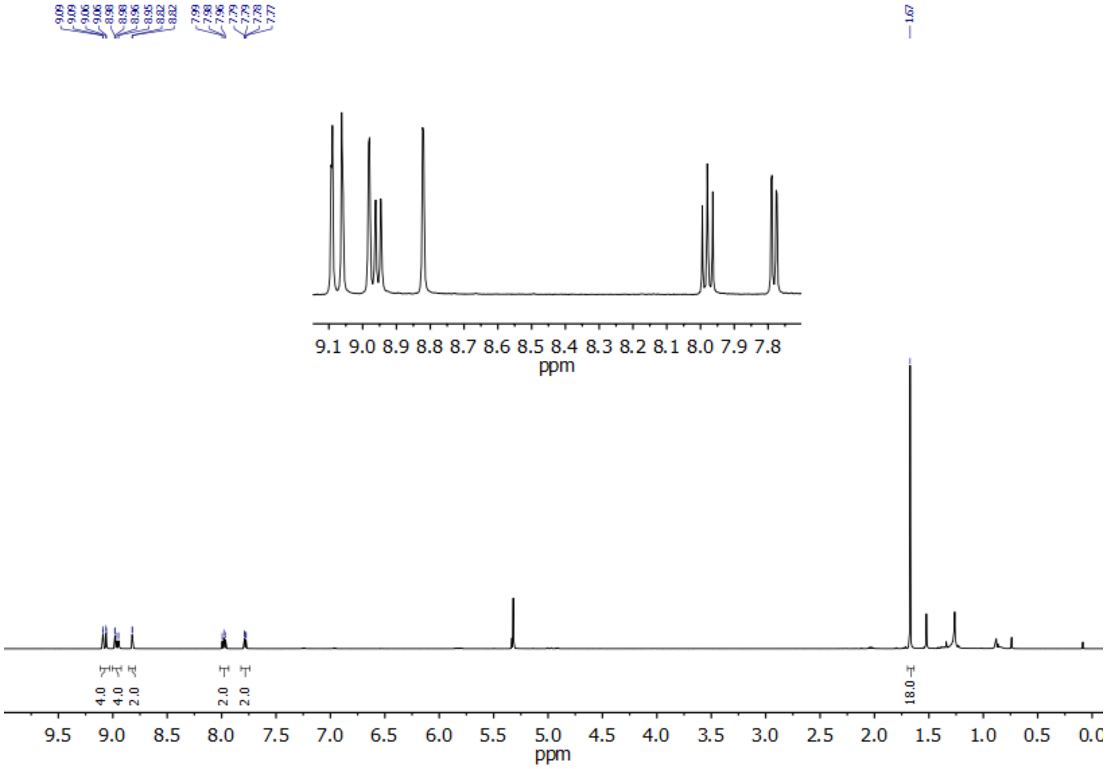

Figure S17. ^{1}H NMR (500 MHz, CD_2Cl_2) spectrum of **8b**.

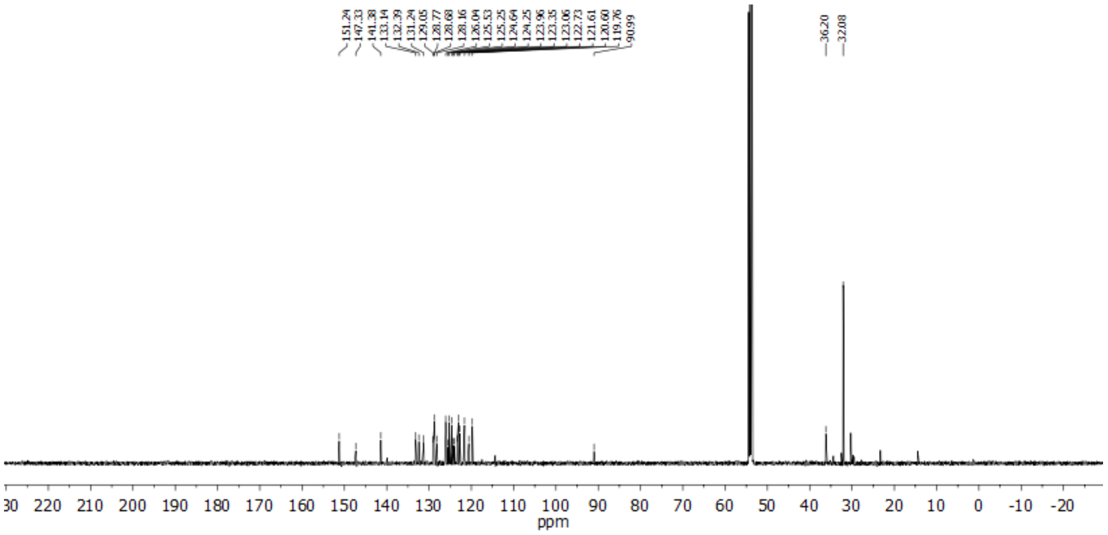

Figure S18. $^{13}C\{^{1}H\}$ NMR (126 MHz, CD_2Cl_2) spectrum of **8b**.

Compound 1:

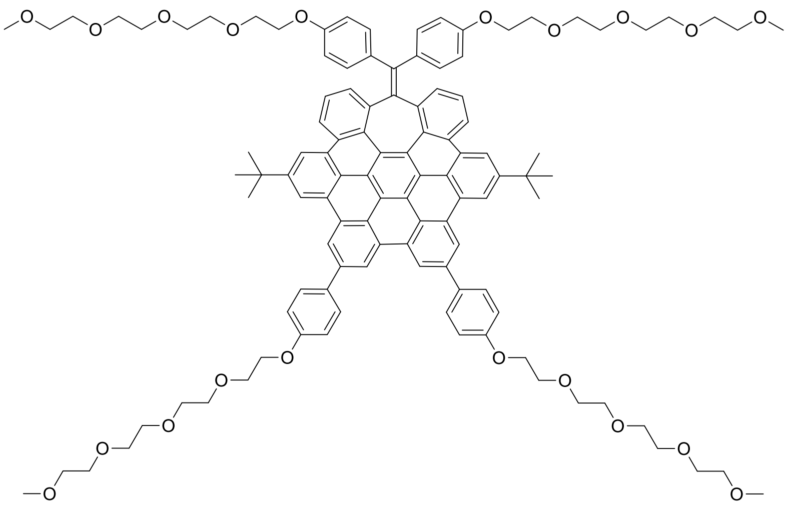

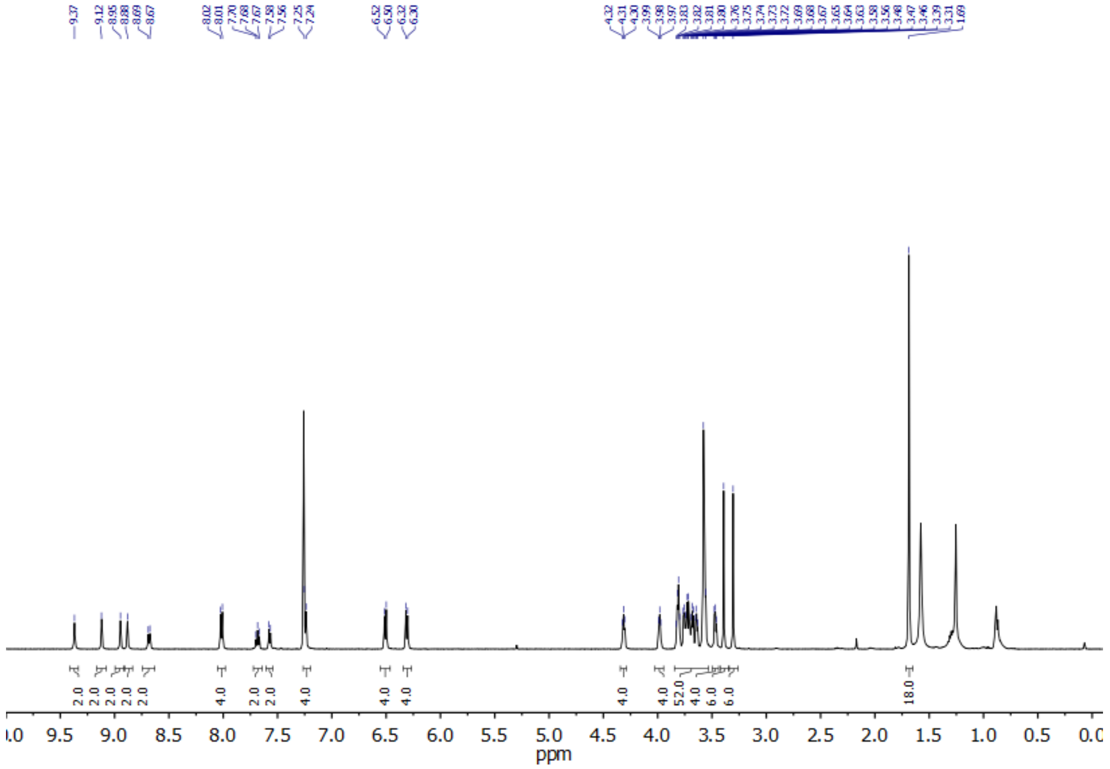

Figure S19. ^{1}H NMR (500 MHz, $CDCl_3$) spectrum of **1**.

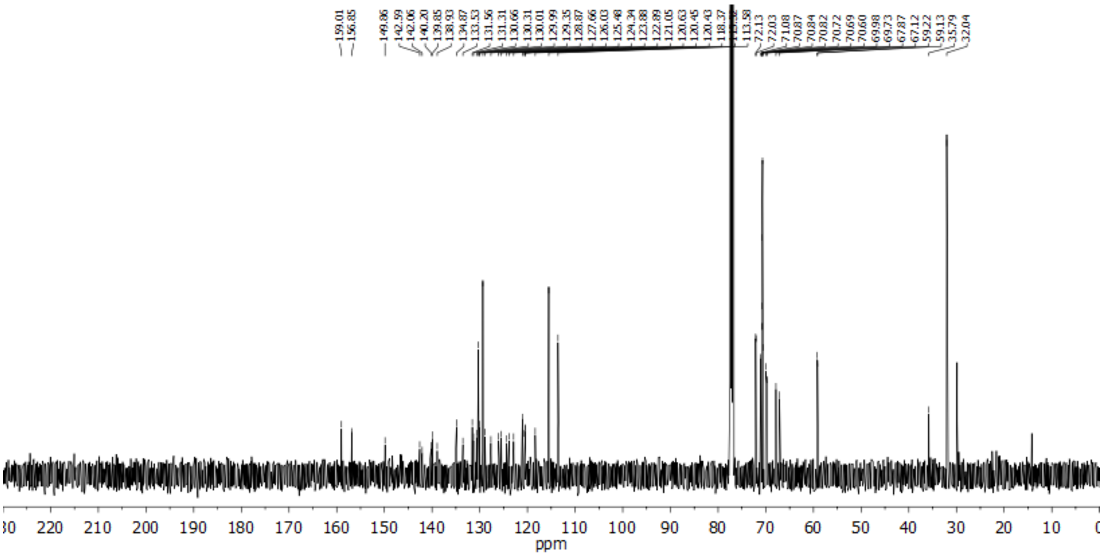

Figure S20. $^{13}C\{^{1}H\}$ NMR (126 MHz, $CDCl_3$) spectrum of **1**.

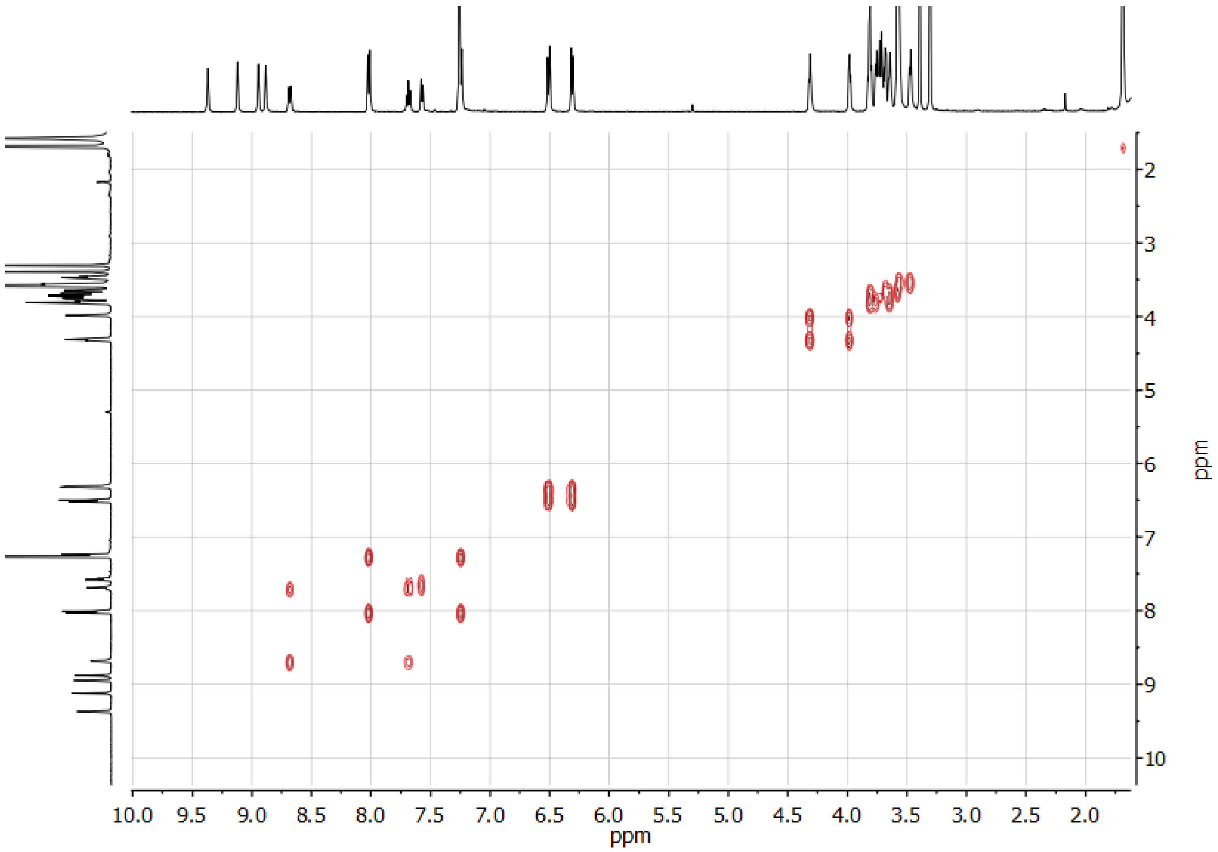

Figure S21. COSY NMR (500 MHz, $CDCl_3$) spectrum of **1**.

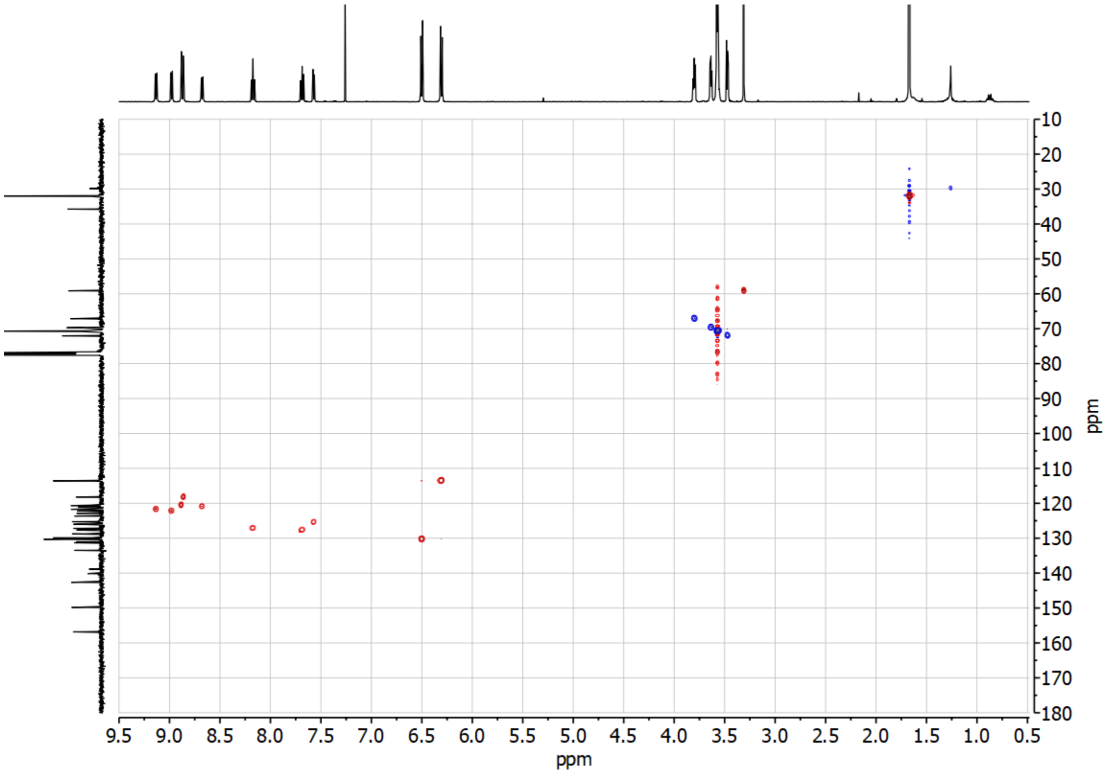

Figure S22. HSQC NMR (500 MHz and 126 MHz, $CDCl_3$) spectrum of **1**.

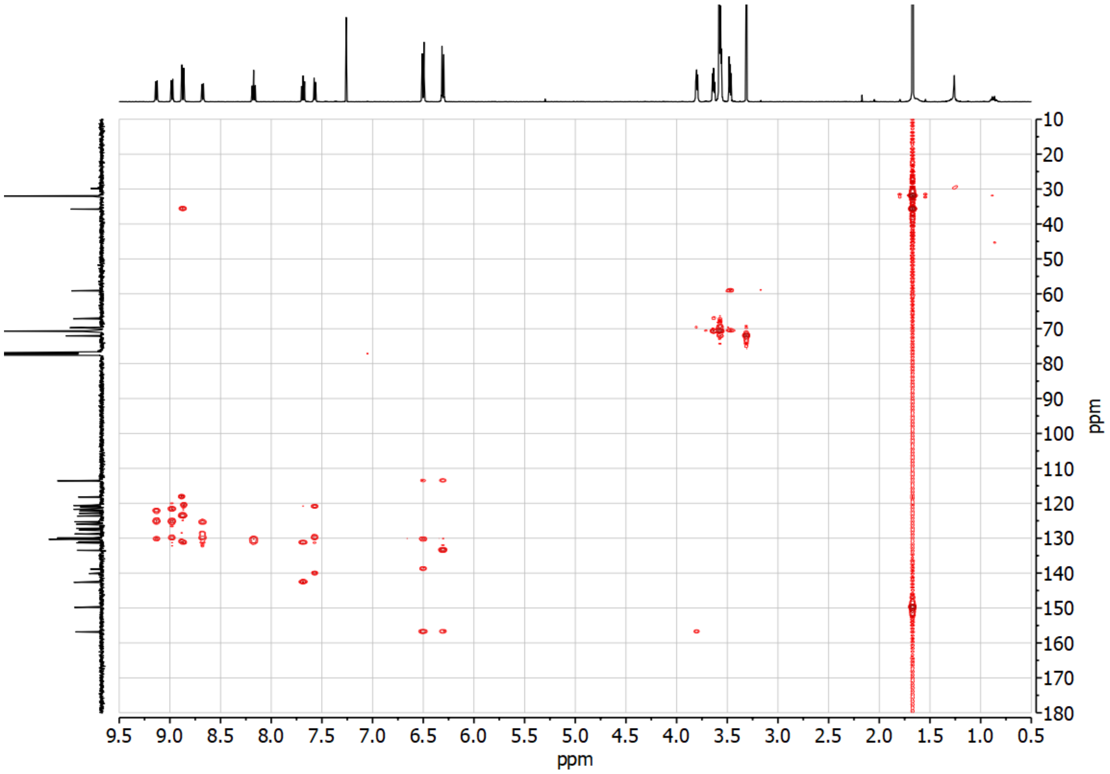

Figure S23. HMBC NMR (500 MHz and 126 MHz, $CDCl_3$) spectrum of **1**.

Compound 2:

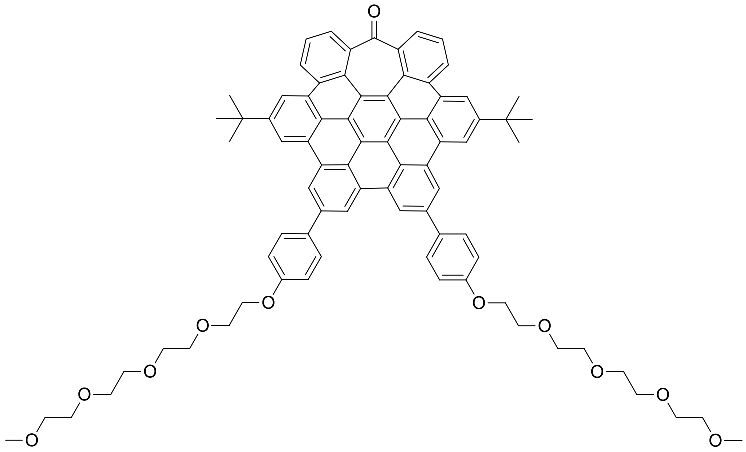

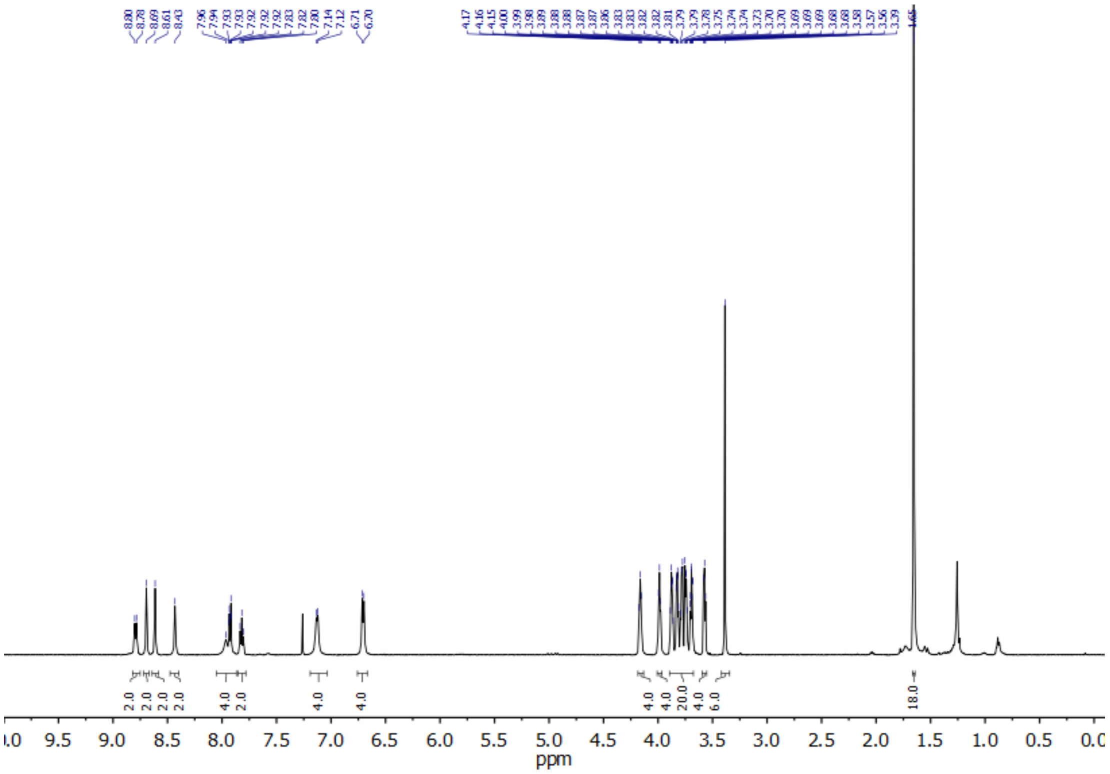

Figure S24. ^{1}H NMR (500 MHz, $CDCl_3$) spectrum of **2**.

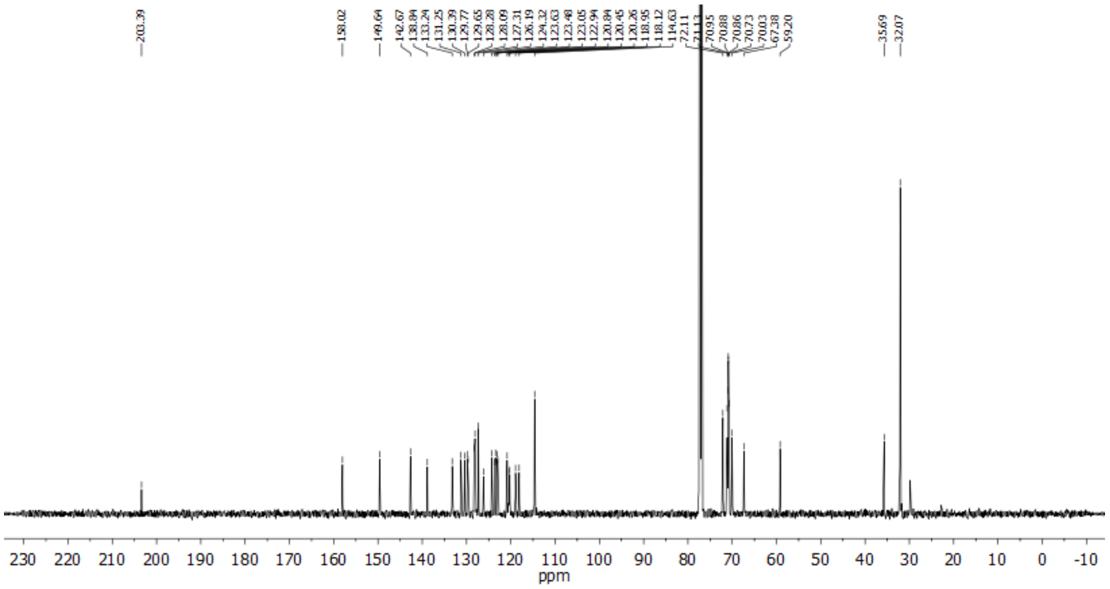

Figure S25. $^{13}C\{^{1}H\}$ NMR (126 MHz, $CDCl_3$) spectrum of **2**.

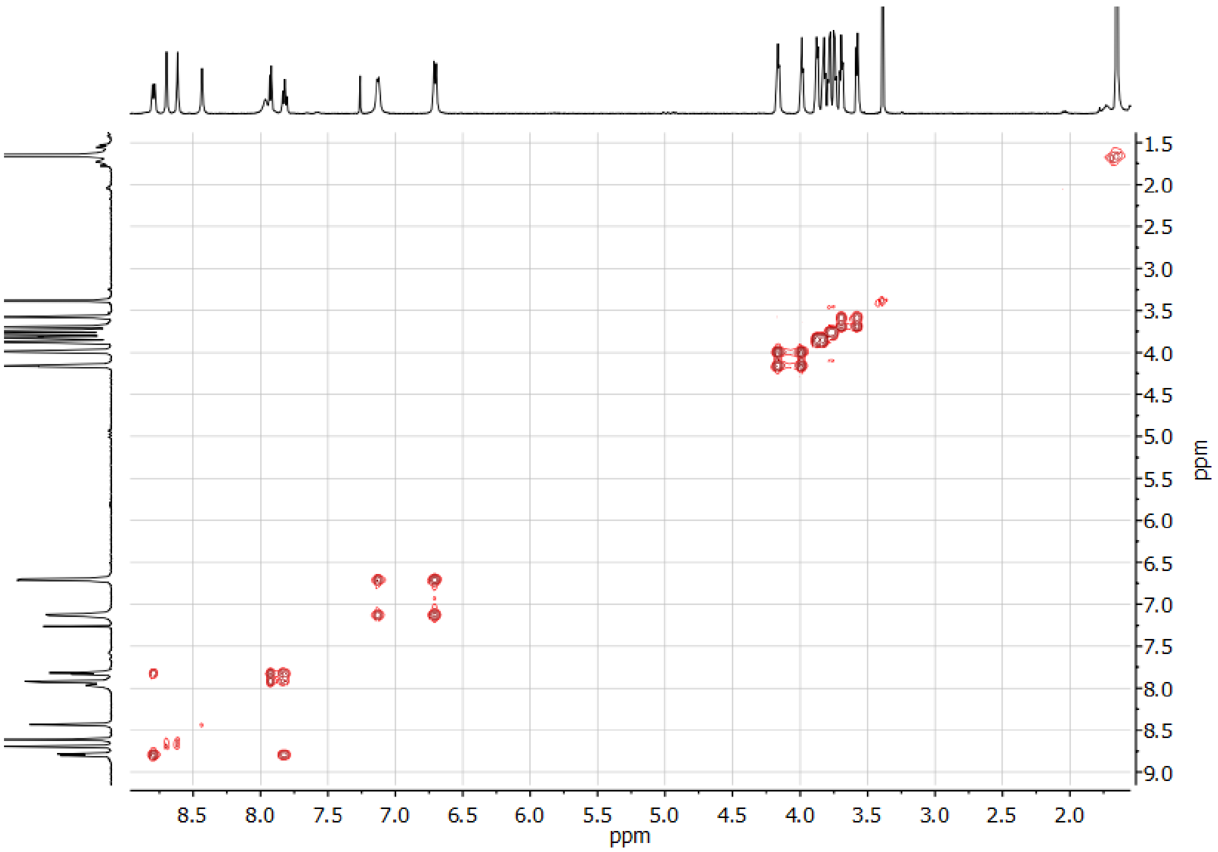

Figure S26. COSY NMR (500 MHz, $CDCl_3$) spectrum of **2**.

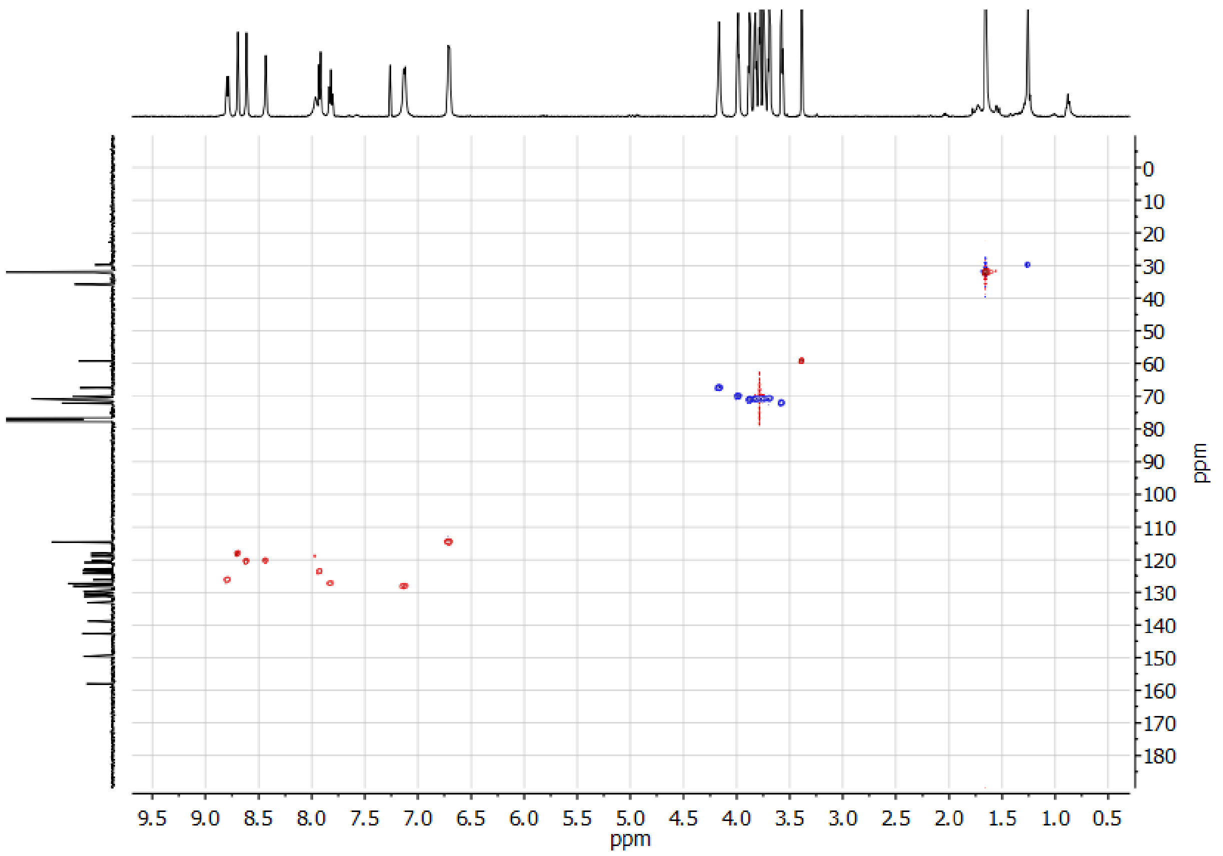

Figure S27. HSQC NMR (500 MHz and 126 MHz, $CDCl_3$) spectrum of **2**.

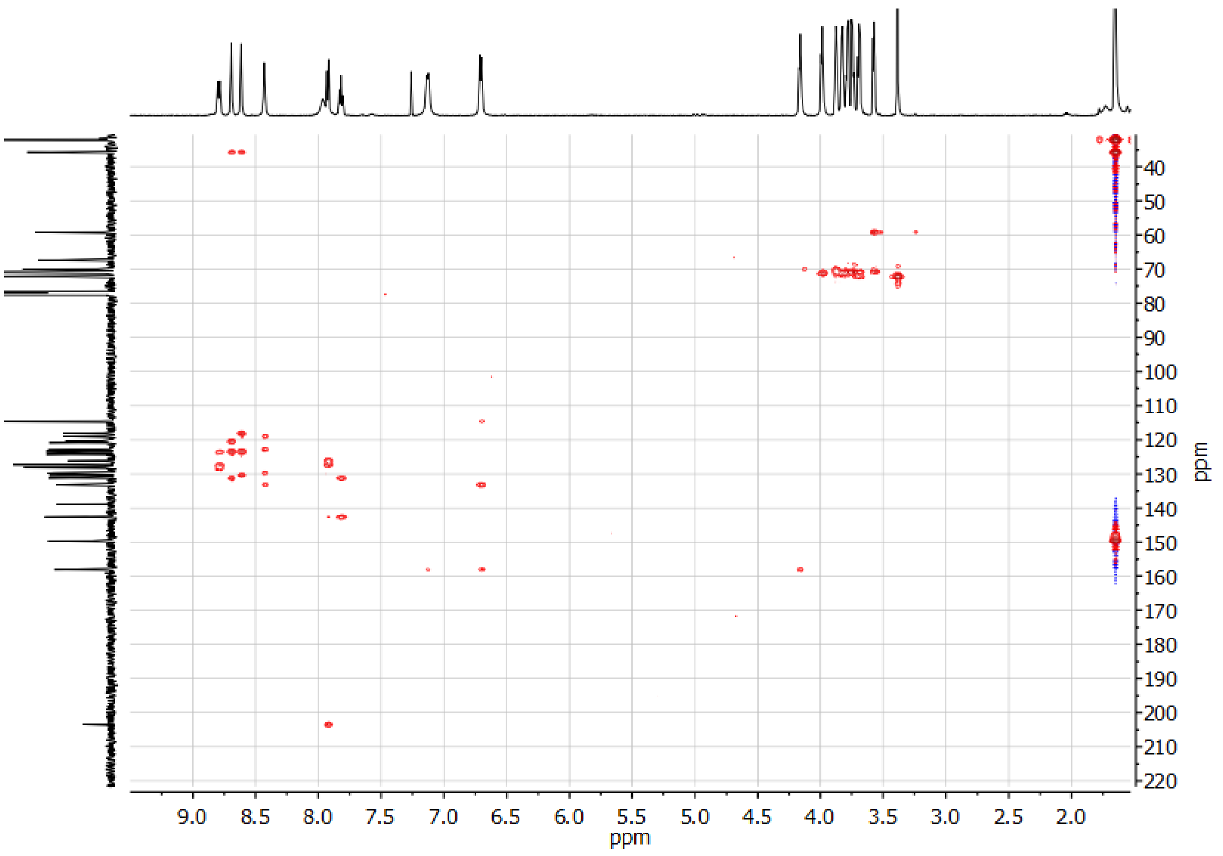

Figure S28. HMBC NMR (500 MHz and 126 MHz, $CDCl_3$) spectrum of **2**.

Compound 3:

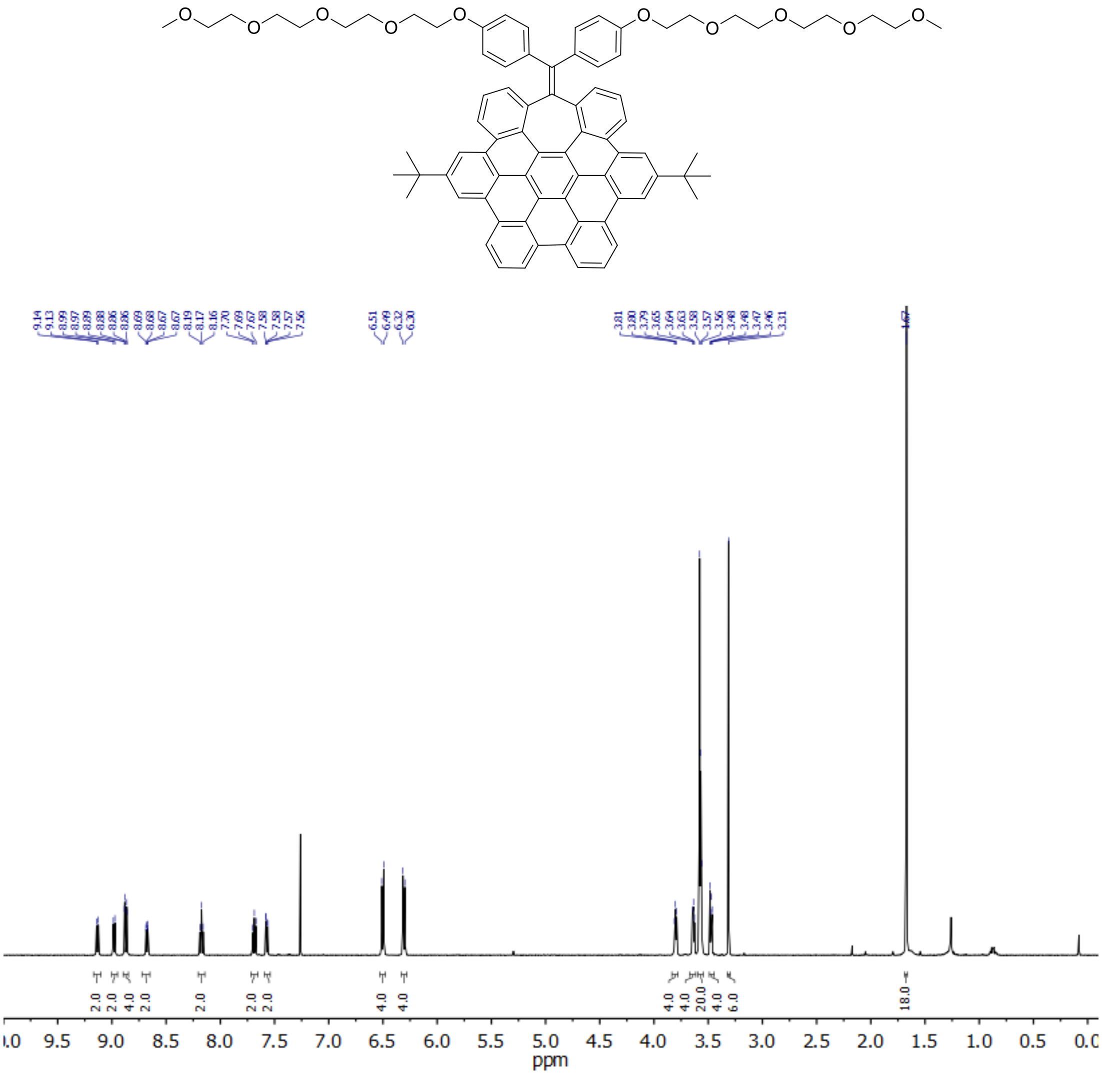

Figure S29. ^{1}H NMR (500 MHz, $CDCl_3$) spectrum of **3**.

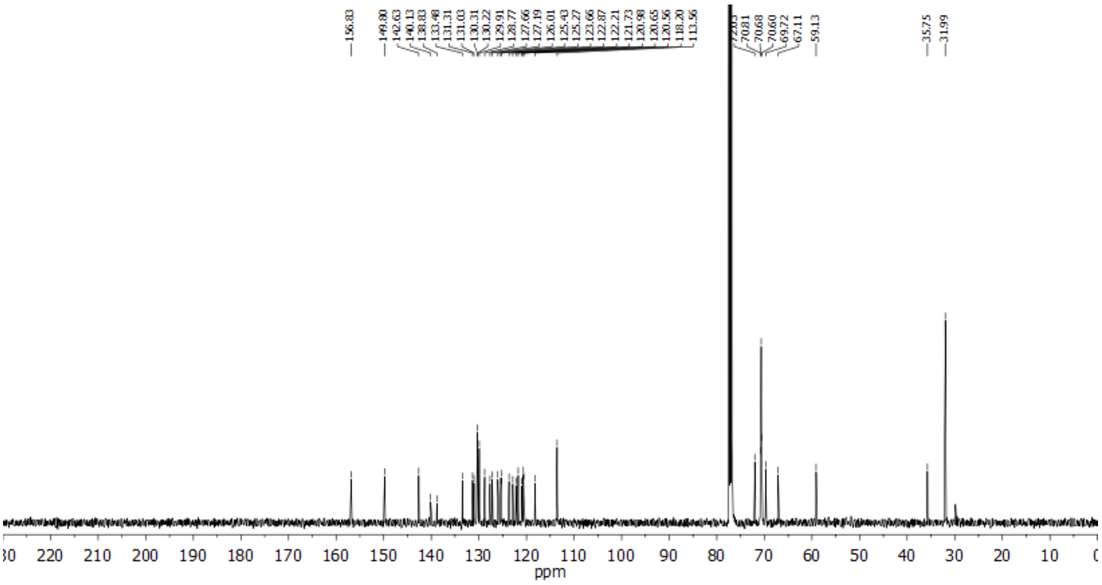

Figure S30. $^{13}C\{^1H\}$ NMR (126 MHz, $CDCl_3$) spectrum of **3**.

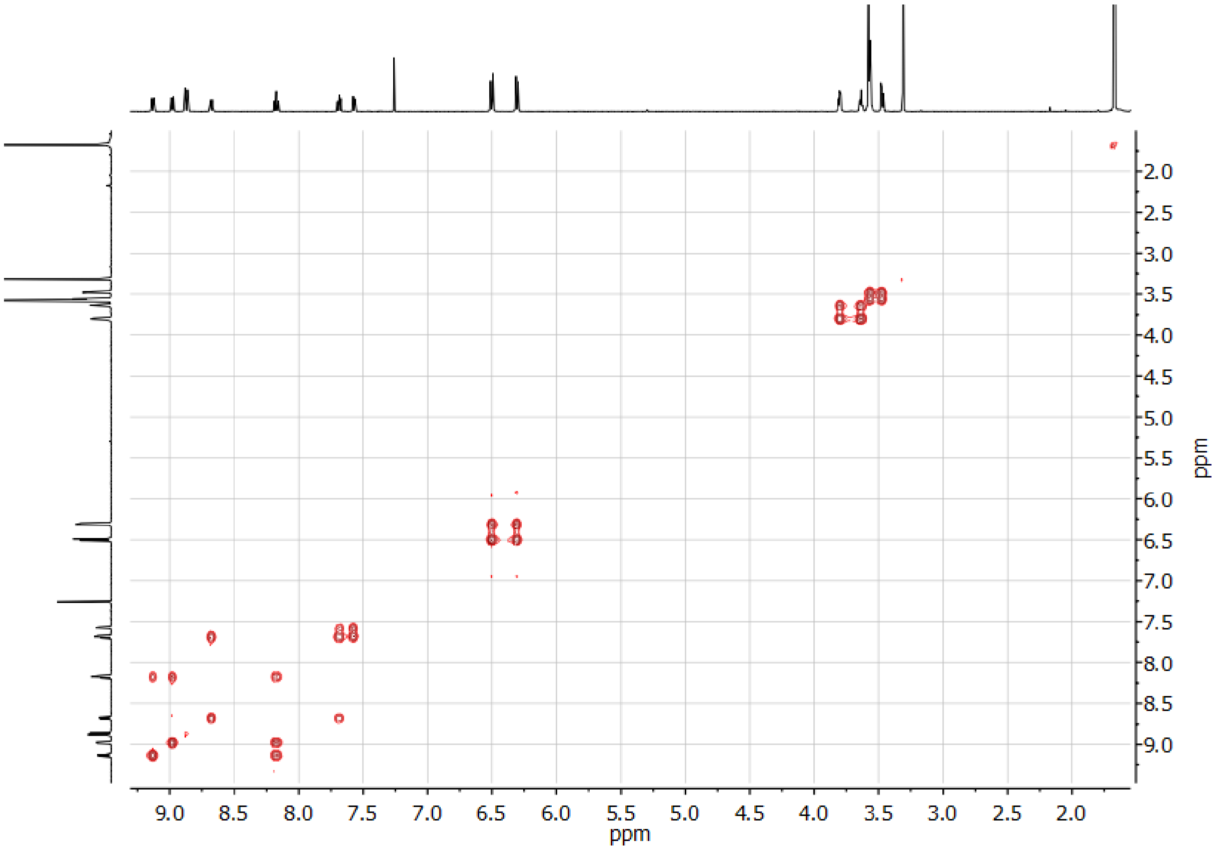

Figure S31. COSY NMR (500 MHz, $CDCl_3$) spectrum of **3**.

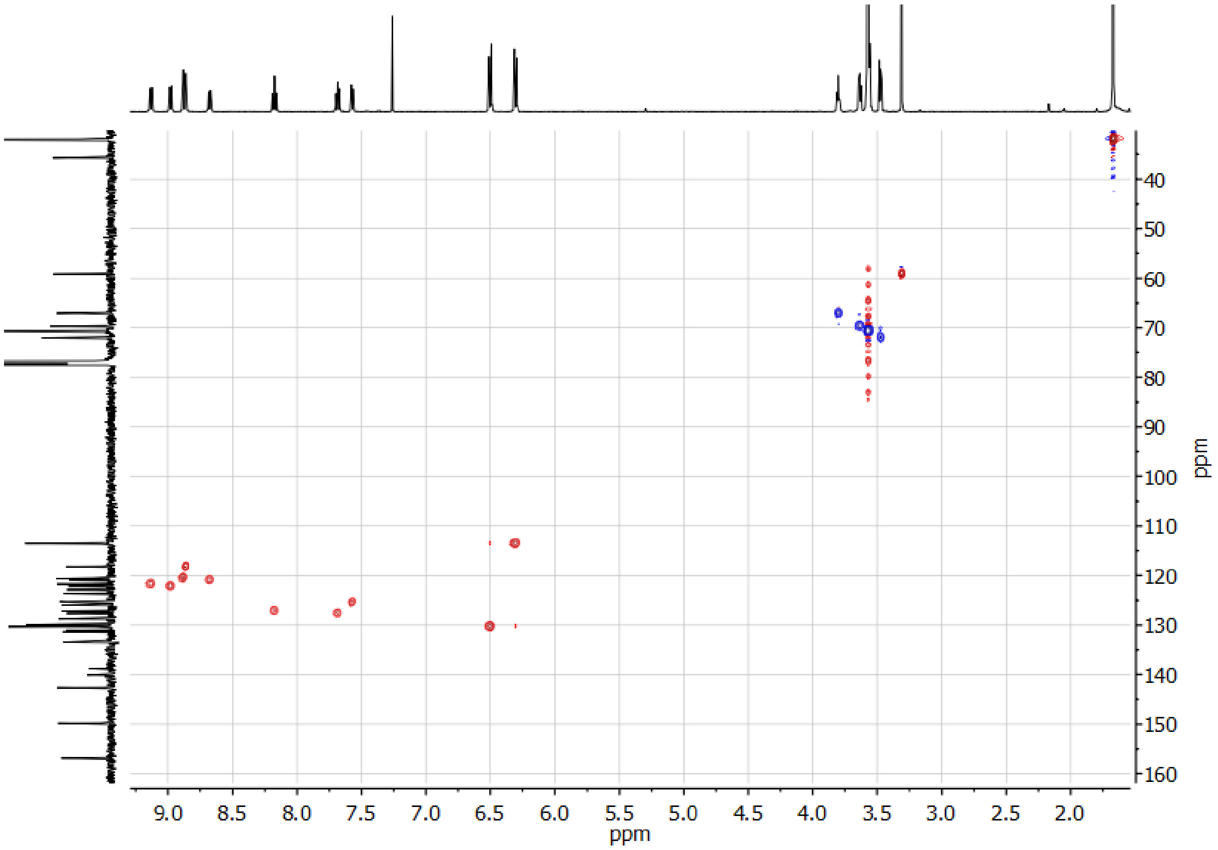

Figure S32. HSQC NMR (500 MHz and 126 MHz, $CDCl_3$) spectrum of **3**.

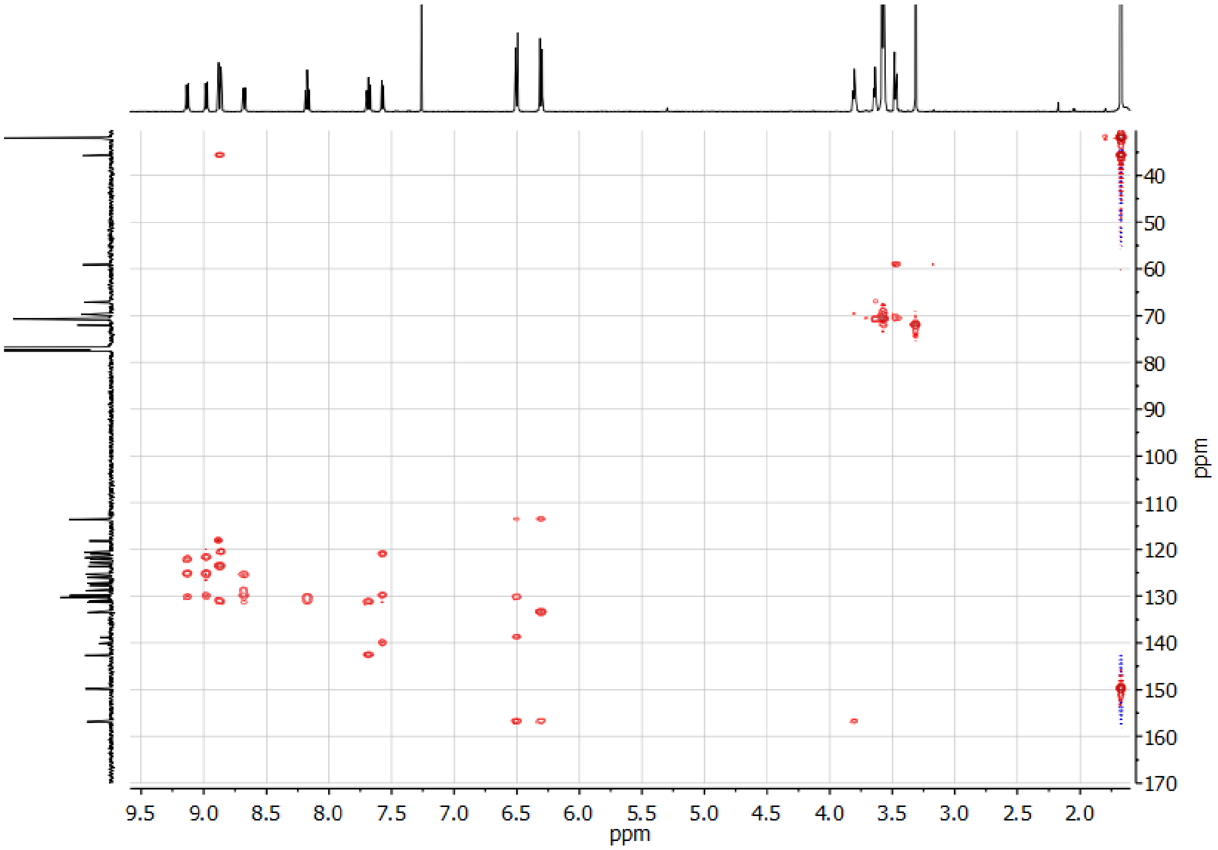

Figure S33. HMBC NMR (500 MHz and 126 MHz, $CDCl_3$) spectrum of **3**

3. ^{1}H NMR spectra of known compounds

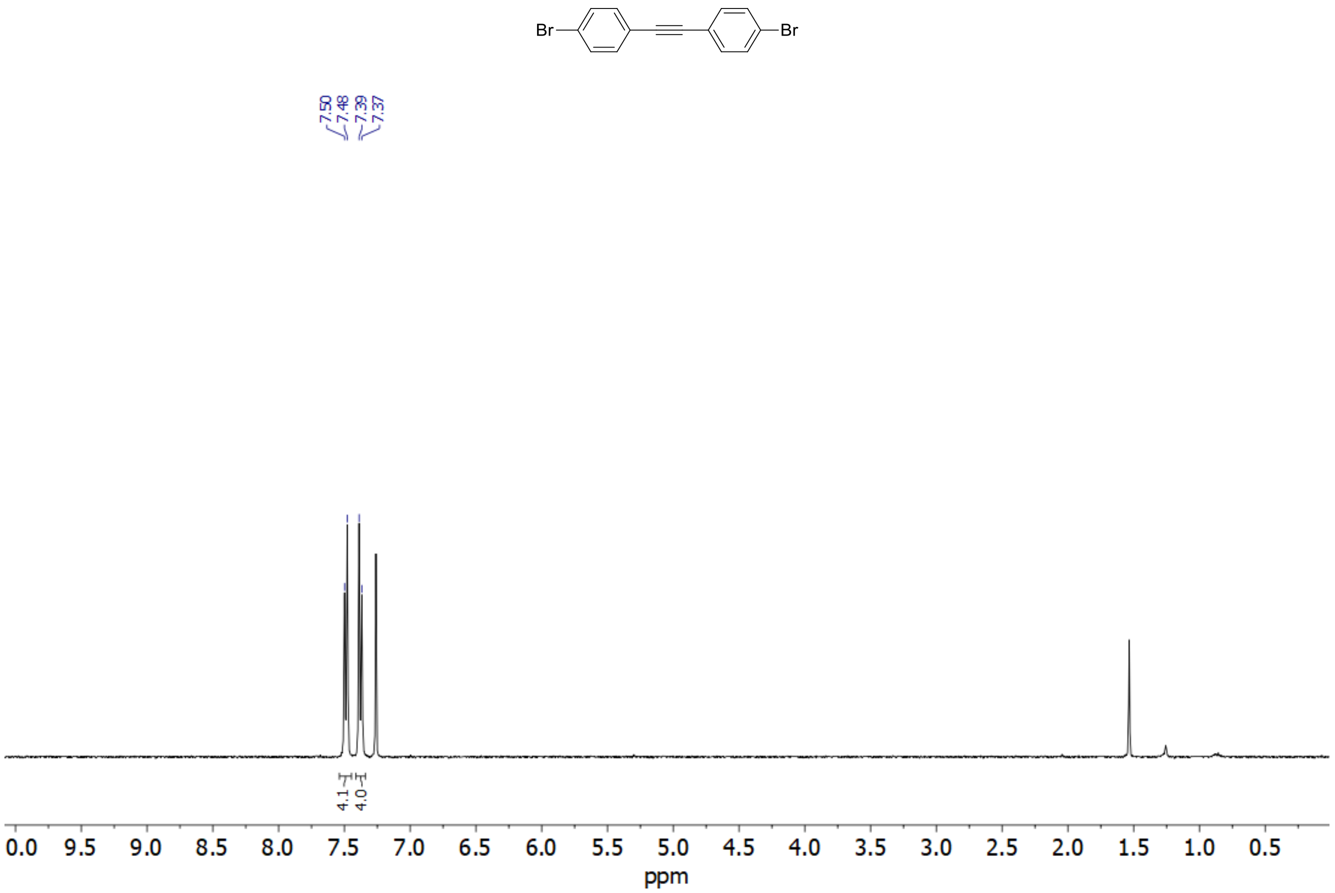

Figure S34. ^{1}H NMR (400 MHz, $CDCl_3$) spectrum of **5b**.

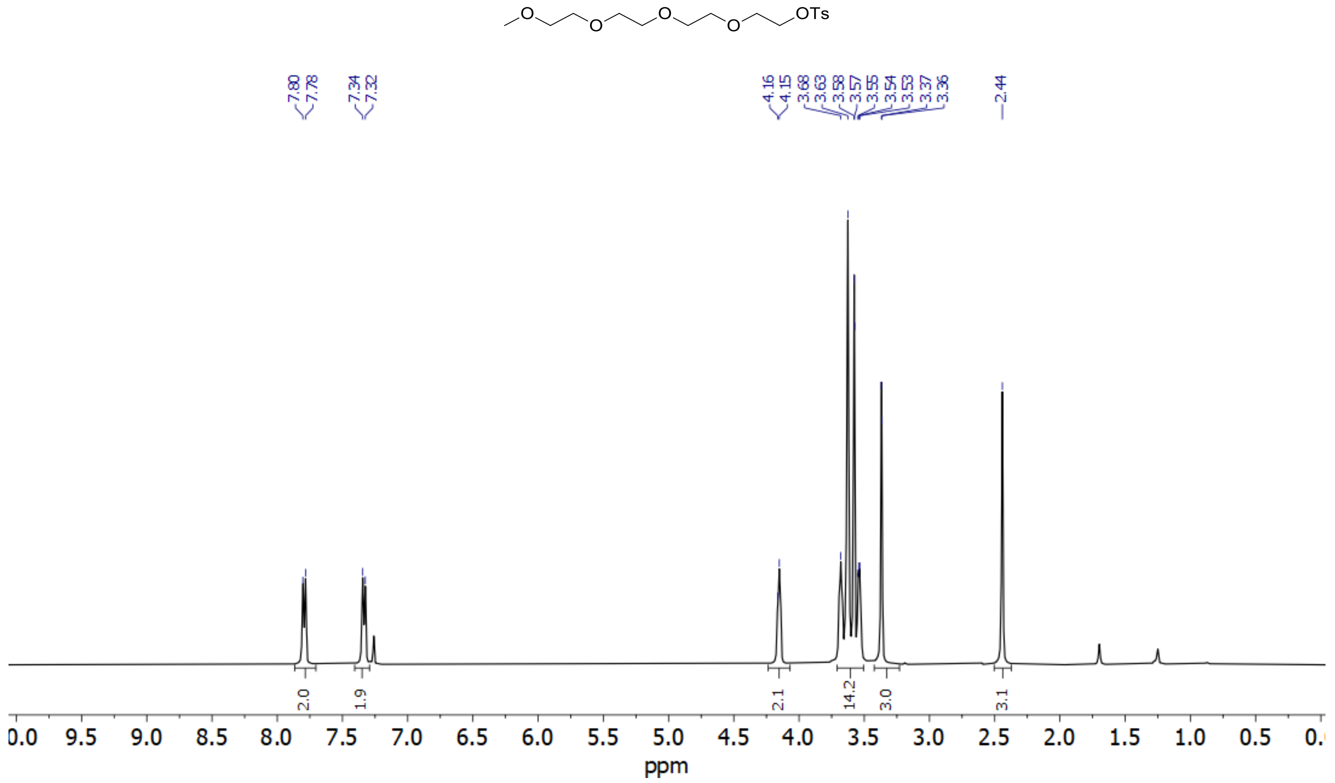

Figure S35. ^{1}H NMR (400 MHz, $CDCl_3$) spectrum of **10**.

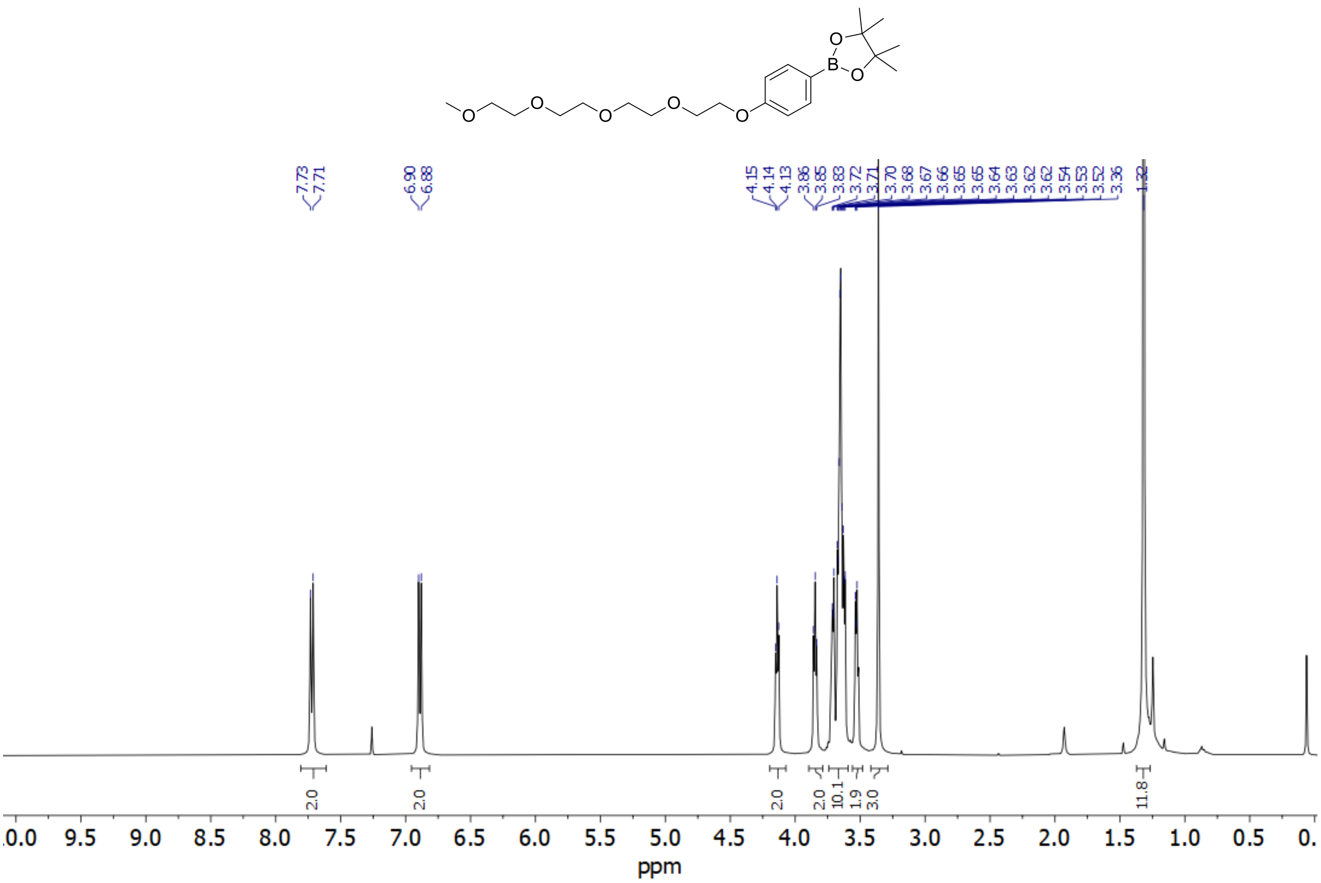

Figure S36. ^{1}H NMR (400 MHz, $CDCl_3$) spectrum of **11**.

5. HRMS spectra of final compounds

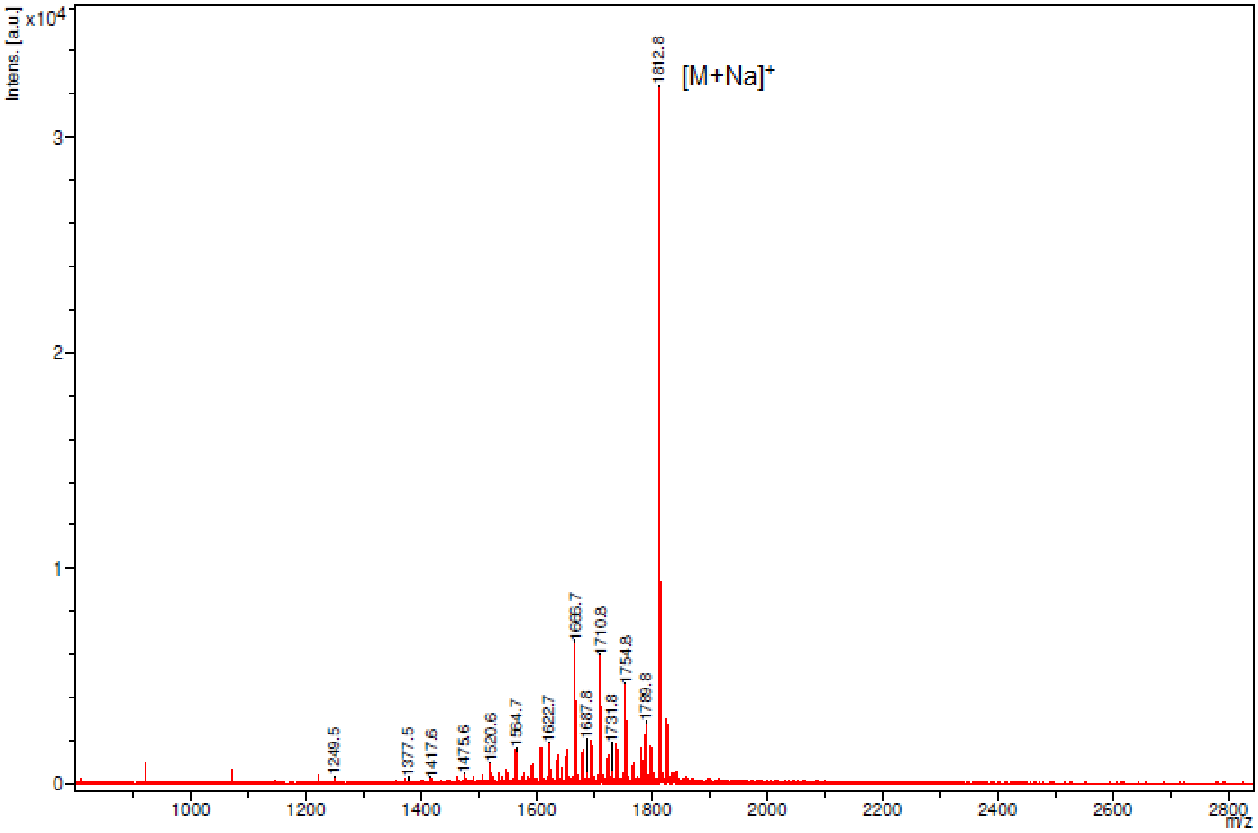

Figure S37. MS (MALDI$^+$) spectrum of **1**

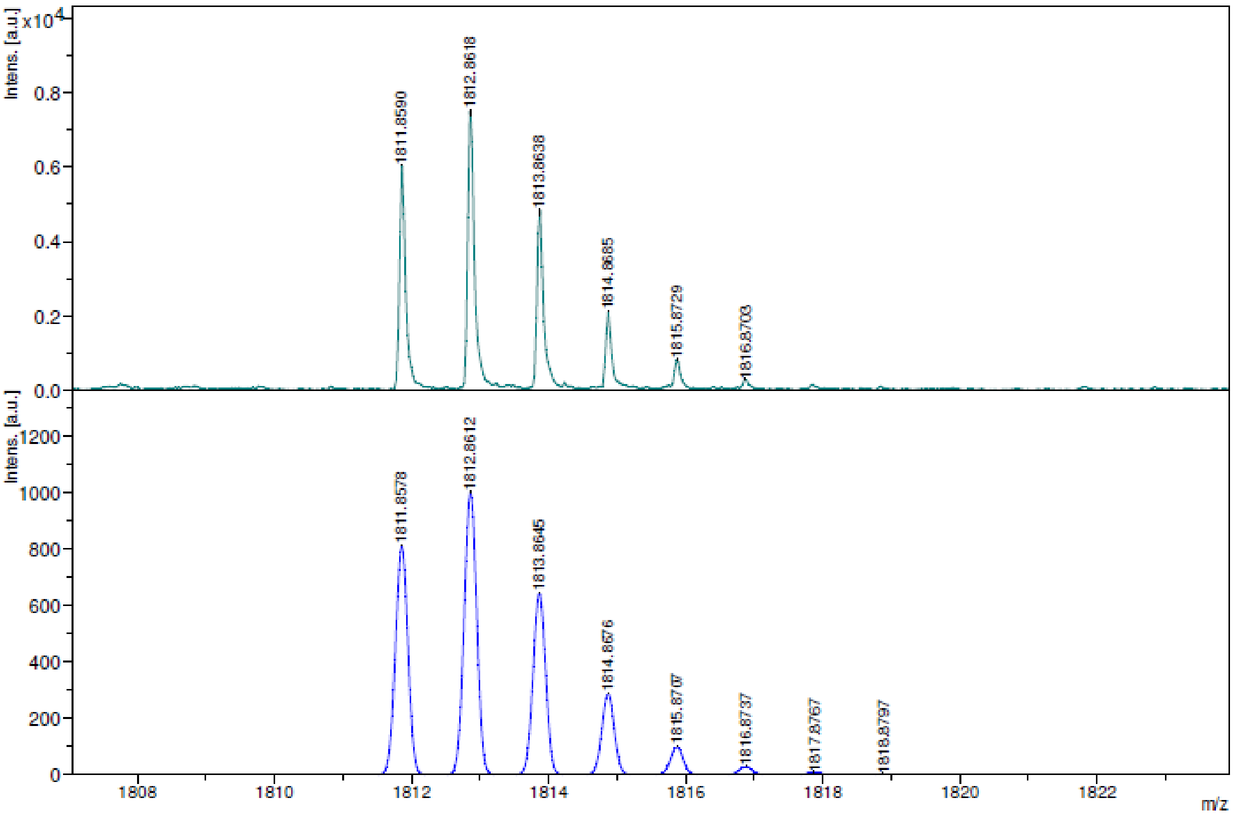

Figure S38. Observed (top) and calculated (bottom) isotopic distribution for [**1**+Na]$^+$.

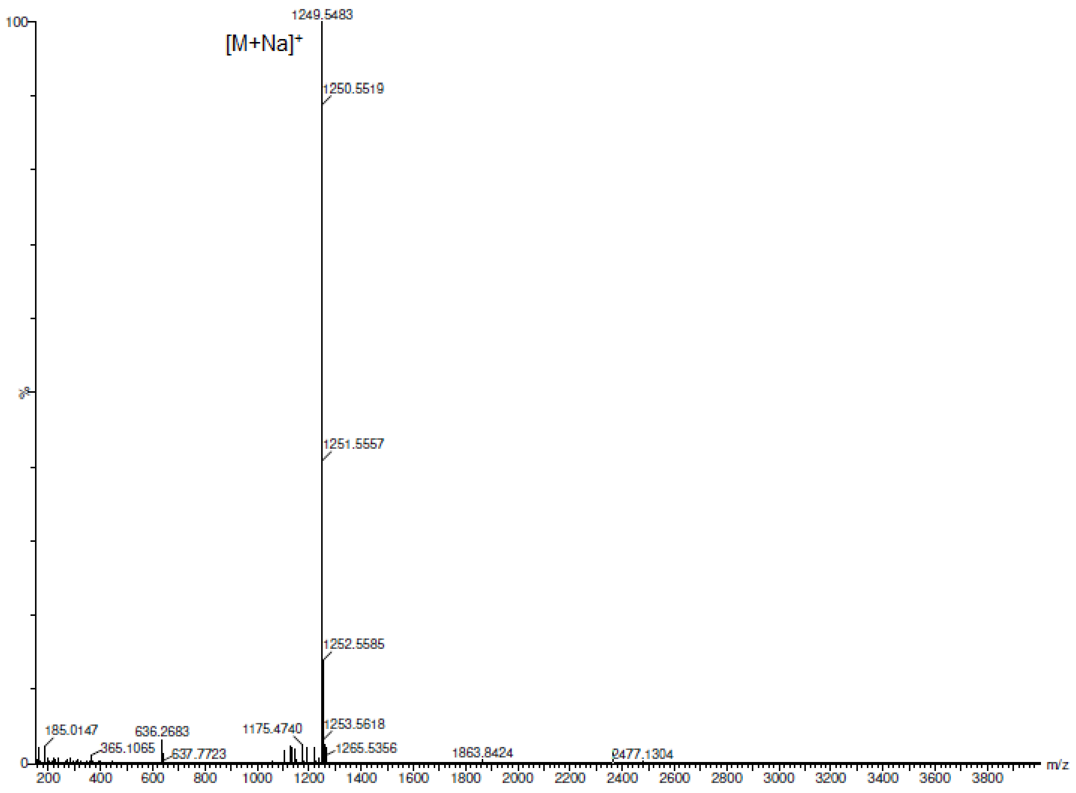

Figure S39. HRMS (ESI^+) spectrum of **2**.

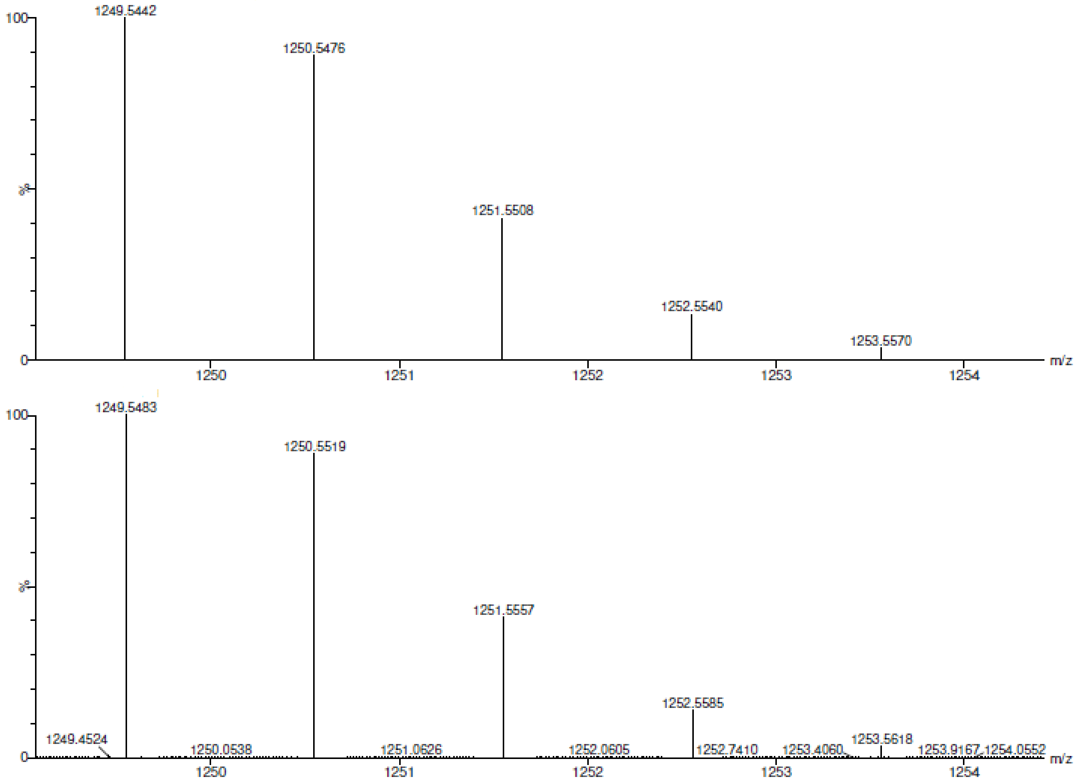

Figure S40. Observed (bottom) and calculated (top) isotopic distribution for $[\mathbf{2}+Na]^+$.

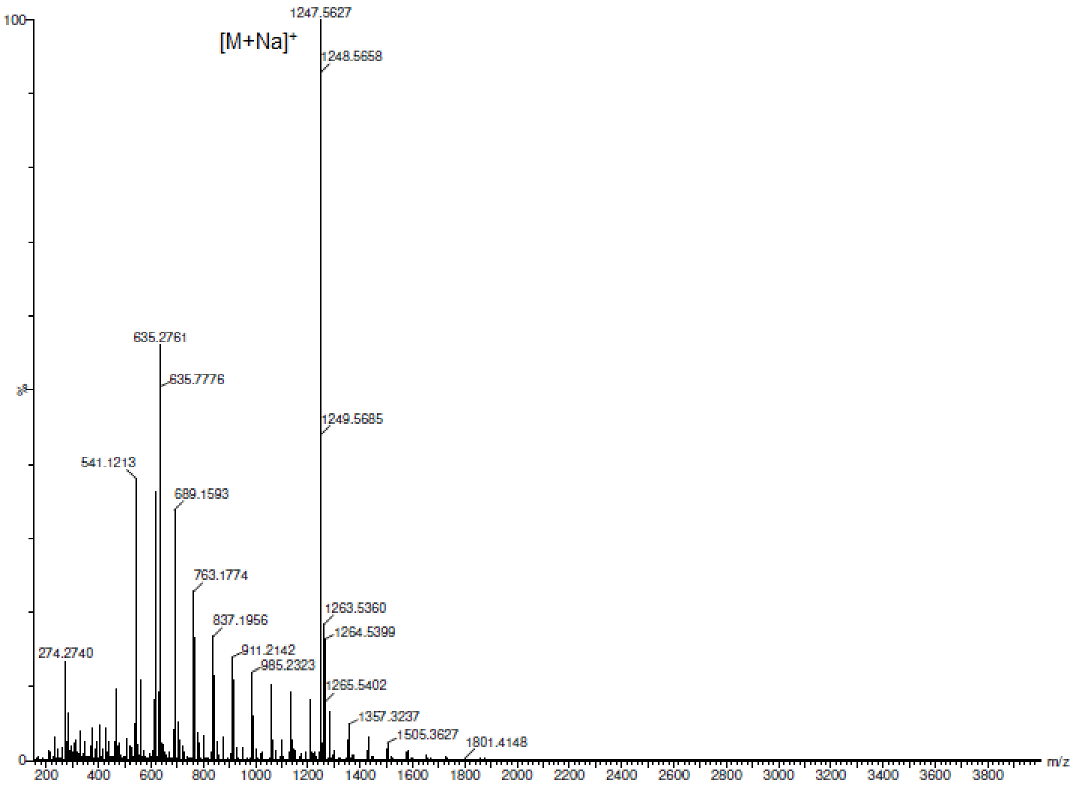

Figure S41. HRMS (ESI$^+$) spectrum of **3**.

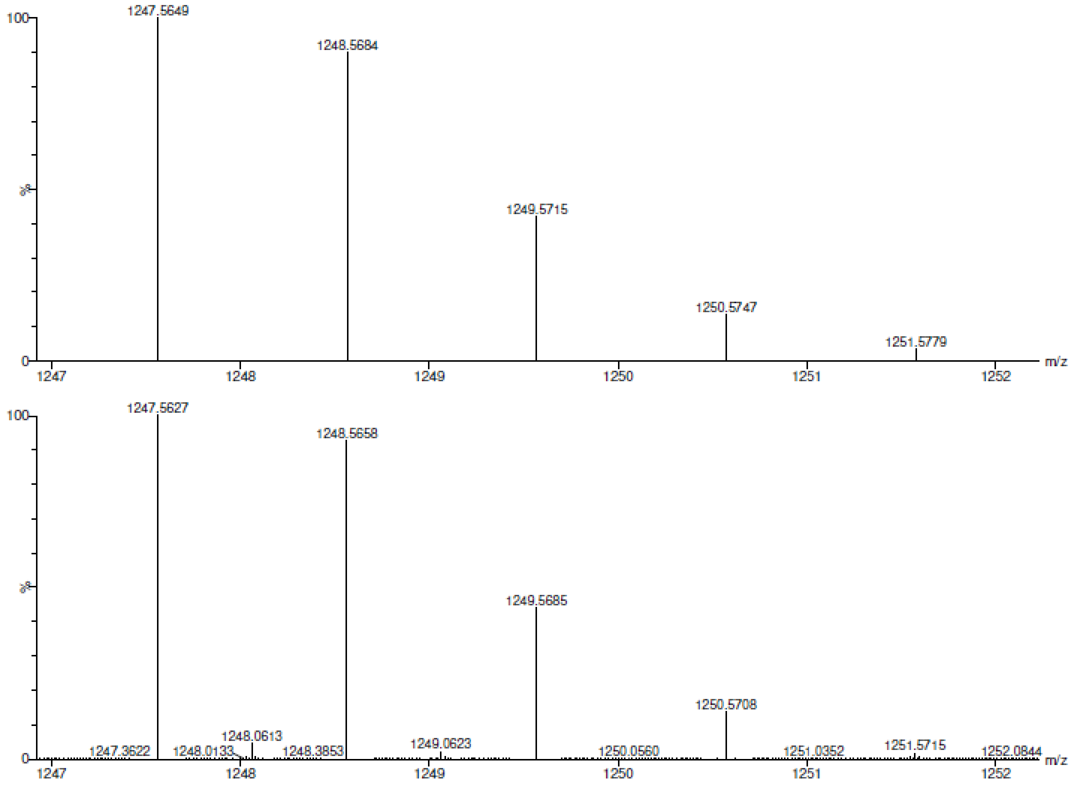

Figure S42. Observed (bottom) and calculated (top) isotopic distribution for [**3**+Na]$^+$.

6. IR spectra of final compounds

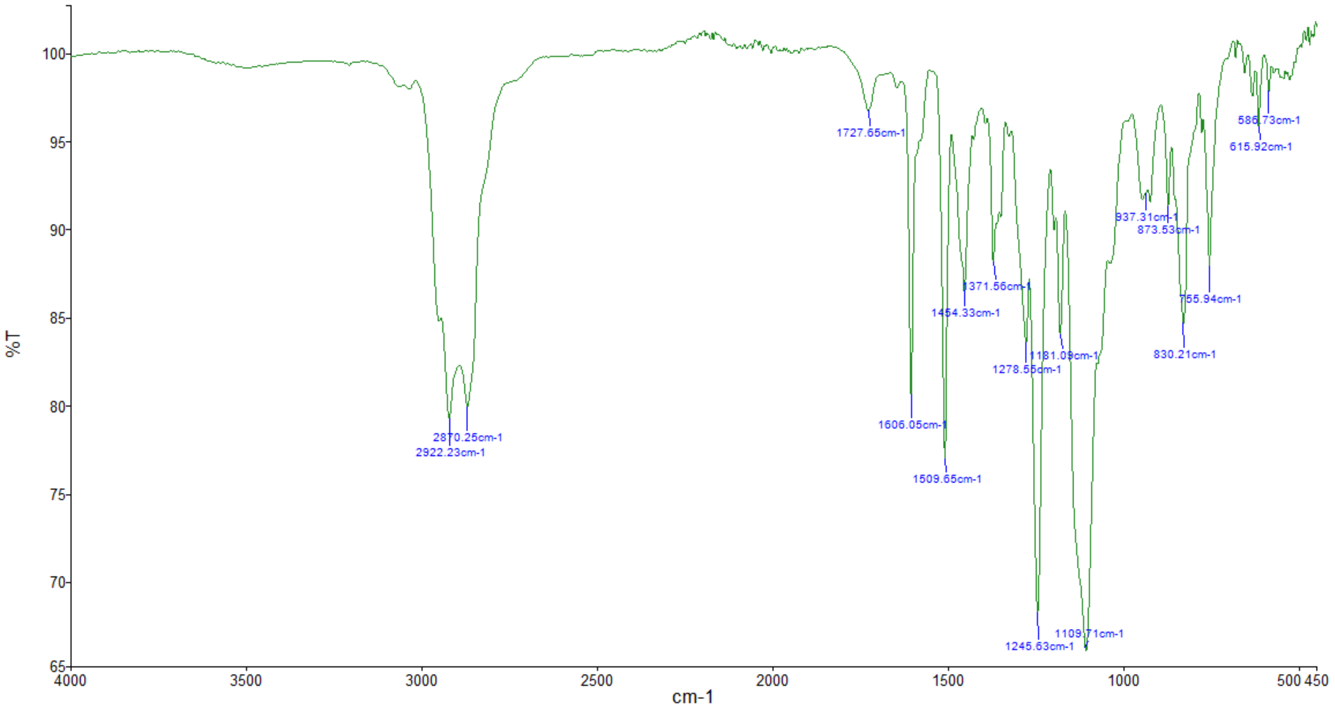

Figure S43. IR (neat) spectrum of **1**.

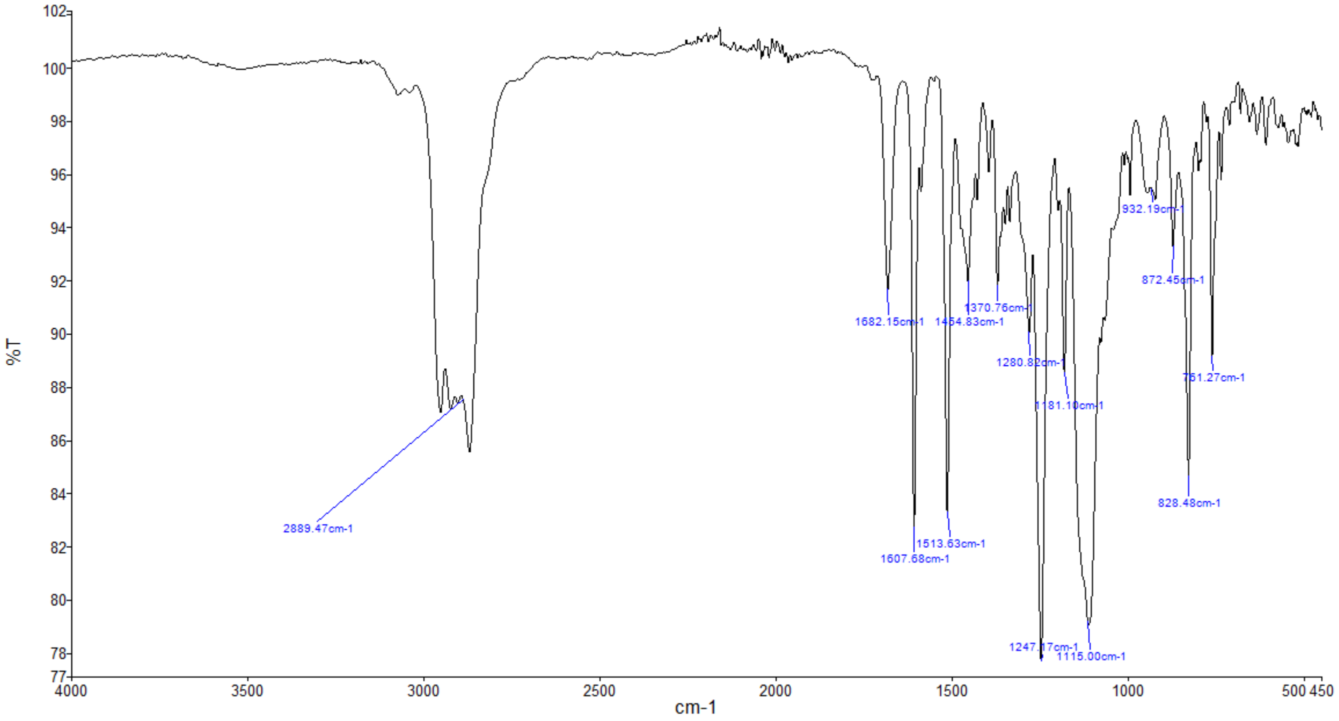

Figure S44. IR (neat) spectrum of **2**.

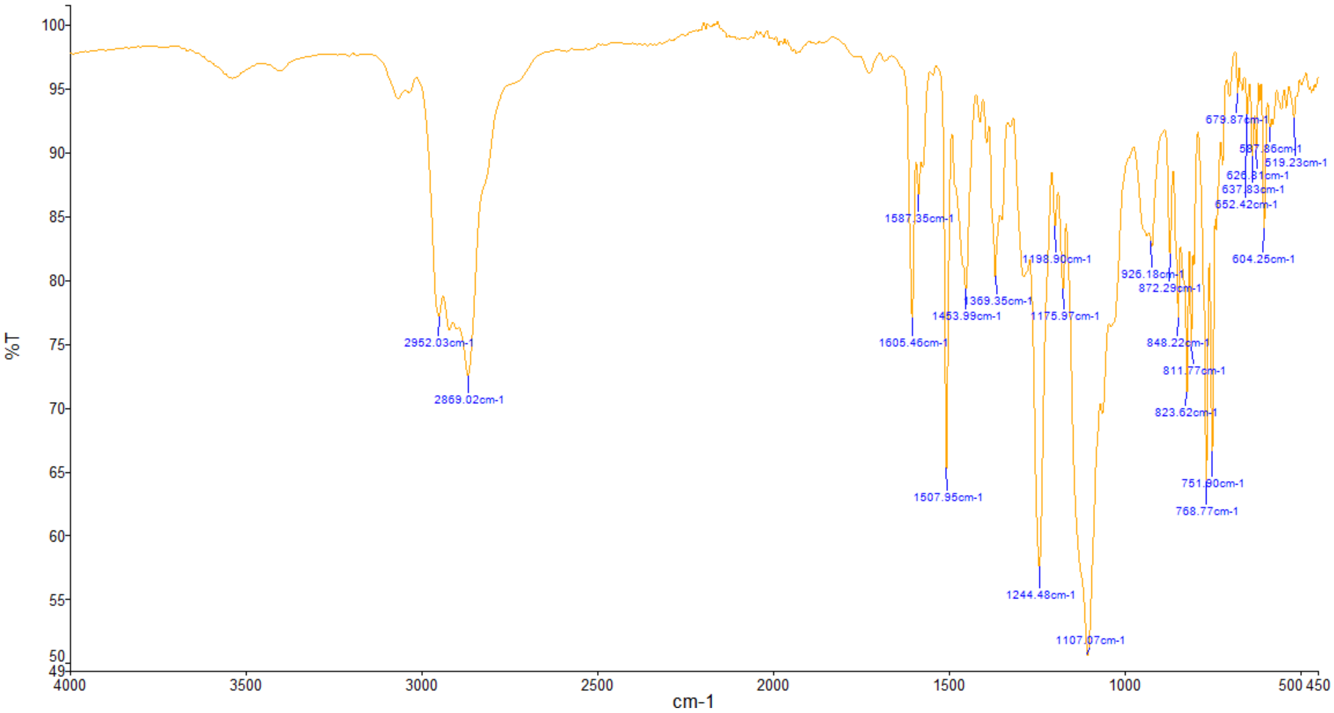

Figure S45. IR (neat) spectrum of **3**.

7. UV-Vis and fluorescence spectra of final compounds

UV-Vis spectra were carried out at room temperature in an Analitik Jena SPECORD® 200 Plus double beam spectrophotometer equipped with a UV-Vis lamp. The spectra of compounds **1-3** were recorded in freshly distilled THF at *ca.* 4×10^{-6} M.

Fluorescence spectra were recorded on an OLIS DSM172 spectrophotometer equipped with a 150 W Xenon lamp. Spectra were recorded at *ca.* 1.5×10^{-5} M in freshly distilled THF at 20 ºC. For fluorescence measurements, a fixed slit-width of 1 mm and 0.5 s of integration time were selected.

For the calculation of the molar extinction coefficient, solutions of each compound in the concentration range $1.0 \times 10^{-6} - 5.0 \times 10^{-5}$ M (depending on the nanographene) in freshly distilled THF were prepared using volumetric flasks and pipettes and were measured in cuvettes with different path lengths (1 cm or 0.2 cm for concentrated solutions). The absorbance spectra were recorded in the 200-600 nm range. The molar extinction coefficient at each wavelength was obtained from the slope of the least-squares fitting of the Absorbance vs. concentration data. Absorbance data measured in the 0.2 cm cuvette was corrected. Besides, we evaluated the optical properties of **1-3** in THF. UV-vis spectra of **1-3** exhibit an absorption band between 300 and 450 nm (λ_{max} = 358, 361 and 350 nm for **1-3**, respectively). Upon irradiation at the absorption maxima, **1-3** display luminescence, with an emission band in the 400-600 nm region, with maxima at 485, 486 and 453 nm, respectively.

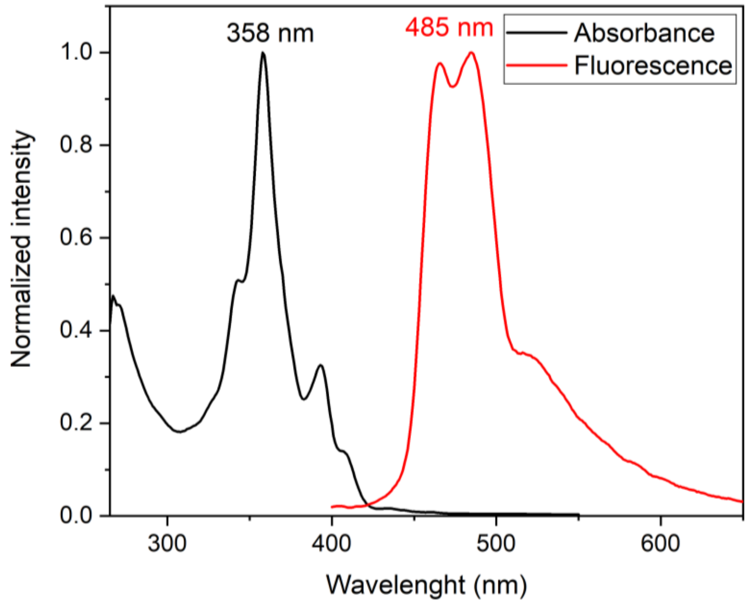

Figure S46. Absorption (black line) and fluorescence emission (λ_{exc} = 360 nm) (red line) normalized spectra (THF) of nanographene **1**.

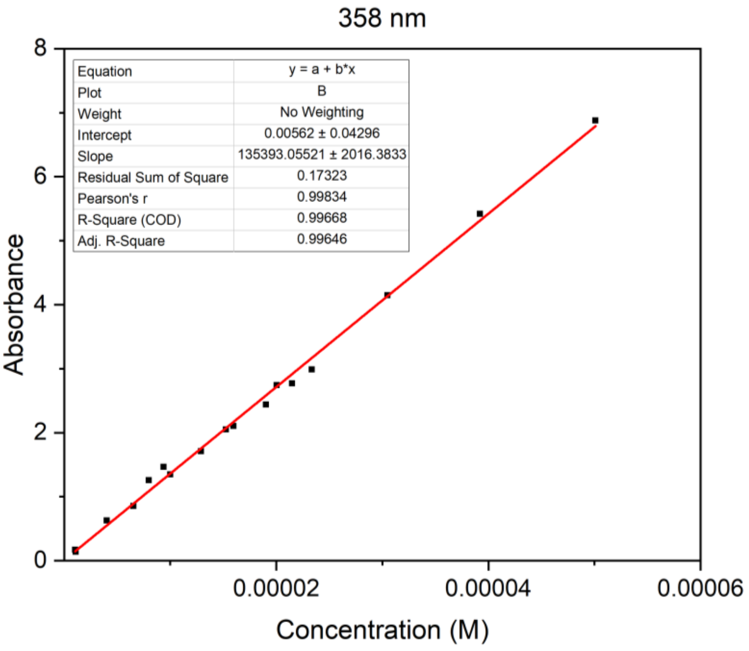

Figure S47. Plot of the absorbance at 358 nm of nanographene **1** vs. concentration. The molar extinction coefficient (ε) is 1.4×10^5 L mol^{-1} cm^{-1} at 358 nm in THF.

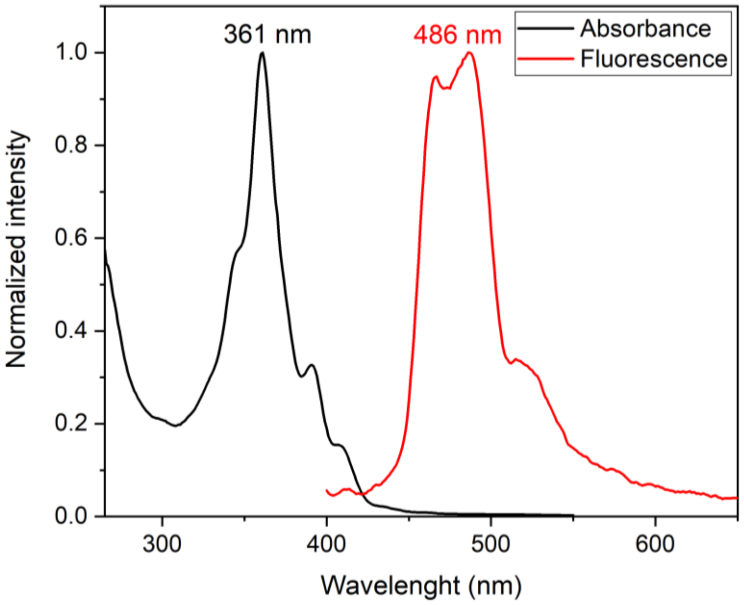

Figure S48. Absorption (black line) and fluorescence emission (λ_{exc} = 362 nm) (red line) normalized spectra (THF) of nanographene **2**.

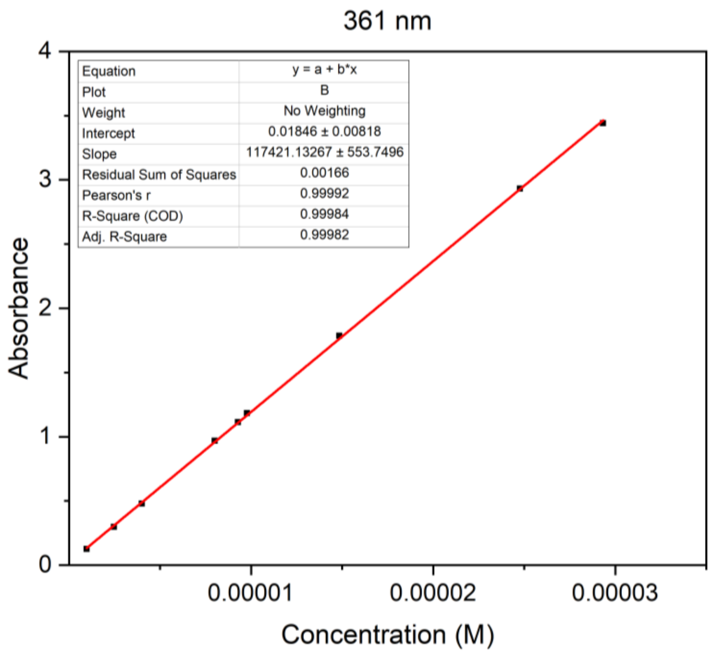

Figure S49. Plot of the absorbance at 361 nm of nanographene **2** vs. concentration. The molar extinction coefficient (ε) is 1.2×10^5 L mol^{-1} cm^{-1} at 361 nm in THF.

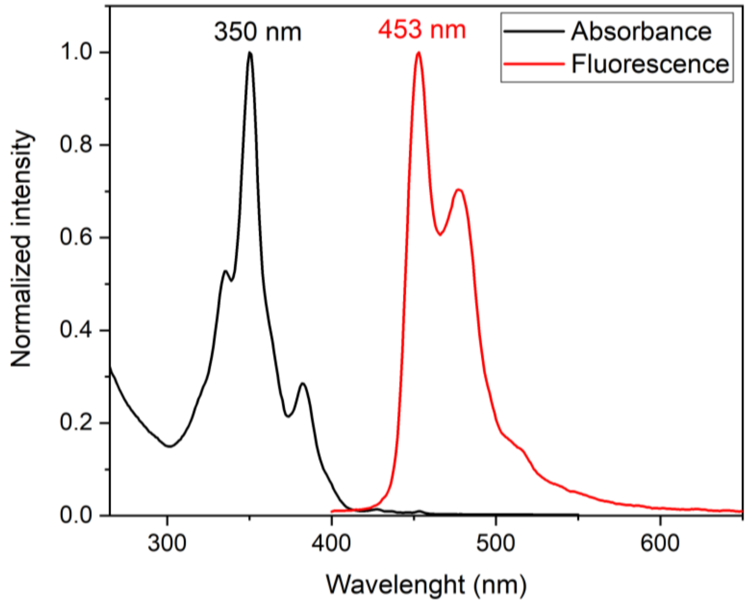

Figure S50. Absorption (black line) and fluorescence emission (λ_{exc} = 352 nm) (red line) normalized spectra (THF) of nanographene **3**.

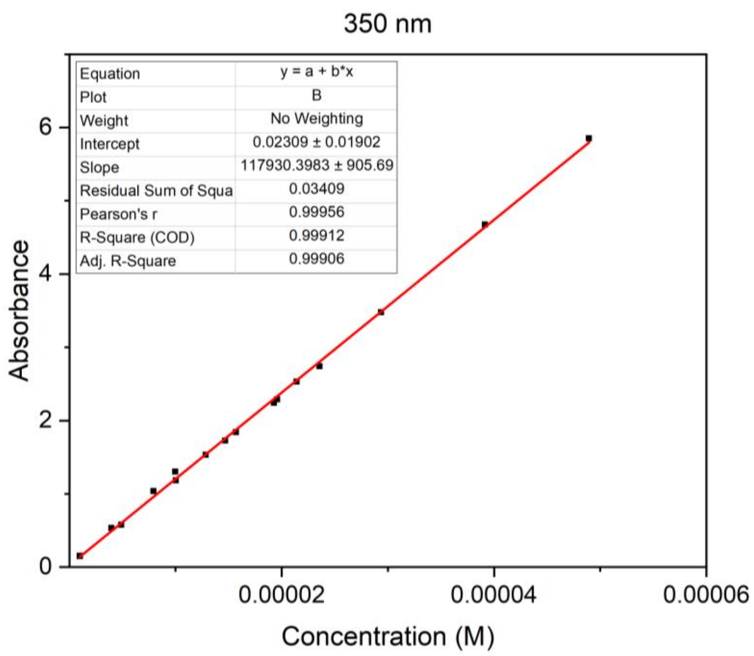

Figure S51. Plot of the absorbance at 350 nm of nanographene **3** vs. concentration. The molar extinction coefficient (ε) is 1.2×10^5 L mol^{-1} cm^{-1} at 350 nm in THF.

Quantum yields were determined by measuring both absorbance and fluorescence of compounds **1-3** in freshly distilled THF, using quinine sulfate in 0.1 M H_2SO_4 as standard ($\Phi_r = 0.54$).[S10] For the relative determination of the fluorescence quantum yield Φ in a series of solvents, Eq. 1 was used.[S11, S12]

$$\Phi_x = \Phi_r \times \frac{F_x}{F_r} \times \frac{1-10^{-A_r(\lambda_{ex})}}{1-10^{-A_x(\lambda_{ex})}} \times \frac{n_x^2}{n_r^2} \qquad \text{(Eq. 1)}$$

The subscripts *x* and *r* refer respectively to the sample and a reference (standard) fluorophore with known quantum yield Φ_r in a specific solvent; *F* stands for the spectrally corrected, integrated fluorescence spectra; $A(\lambda_{ex})$ denotes the absorbance at the used excitation wavelength λ_{ex}; and *n* represents the refractive index of the solvent (in principle at the average emission wavelength). To minimize inner filter effects, the absorbance at the excitation wavelength λ_{ex} was kept under 0.1. The measurements were performed using 10×10 mm cuvettes on non-degassed samples.

Table S1. Quantum yields of nanographene **1-3**.

Nanographene	Φ
**1**	4.6 %
**2**	2.0 %
**3**	5.6 %

8. Computational methods

Molecular dynamics (MD) calculations were performed by using Forcite with Dreiding force field implemented in Biovia Materials Studio 2020. The charge was incorporated by using Charge equilibration (QEq). A cutoff of 1.85 nm was used to calculate the LJ interactions and short-range electrostatic interactions. For each MD simulation, the procedure was started with an optimized geometry followed by 20 ps of NVT dynamics to heat the system to 298 K. Finally, after each MD simulation, the geometry was further optimized. A total of 25 annealing cycles were performed with structure minimization after each cycle. Three simulation runs were performed for each system. For monolayers, the XYZ dimensions values of each computational box were adjusted. Thus, eight molecules of **1**, **2** or **3** were introduced into each box, so XY/8 represents the area per molecule at which the simulation run was performed, and Z = 120 Å. Next the water molecules were introduced, adjusting the density to 1 g/cm^3.

9. References

(S1) Oskooie, H. A.; Heravi, M. M.; Behbahani, F. K. A Facile, Mild And Efficient One-Pot Synthesis of 2-Substituted Indole Derivatives Catalyzed By $Pd(PPh_3)_2Cl_2$. *Molecules* **2007**, *12*, 1438–1446. DOI: https://doi.org/10.3390/12071438

(S2) Coulson, D. R.; Satek, L. C.; Grim, S. O. Tetrakis(triphenylphosphine)palladium(0). *Inorg. Synth.* **1972**, *13*, 121–124. DOI: https://doi.org/10.1002/9780470132449.ch23

(S3) Márquez, I. R.; Fuentes, N.; Cruz, C. M.; Puente-Muñoz, V.; Sotorrios, L.; Marcos, M. L.; Choquesillo-Lazarte, D.; Biel, B.; Crovetto, L.; Gómez-Bengoa, E.; González, M. T.; Martin, R.; Cuerva, J. M.; Campaña, A. G. Versatile Synthesis and Enlargement of Functionalized Distorted Heptagon-Containing Nanographenes. *Chem. Sci.* **2017**, *8*, 1068–1074. DOI: https://doi.org/10.1039/C6SC02895K

(S4) David, A. H. G.; Míguez-Lago, S.; Cruz, C. M.; Cuerva, J. M.; Blanco, V.; Campaña, A. G. Self-Association and Complexation Studies with Polycyclic Aromatic Hydrocarbons and Fullerenes. *Org. Mater.* **2021**, *3*, 51–59. DOI: https://doi.org/10.1055/s-0041-1722848

(S5) Wang, J.; Zha, S.; Chen, K.; Zhu, J. Cp*Co(III)-Catalyzed, N–N Bond-Based Redox-Neutral Synthesis of Isoquinolines. *Org. Chem. Front.* **2016**, *3*, 1281–1285. DOI: https://doi.org/10.1039/C6QO00367B

(S6) Cheng, S.; Zong, L.; Yuan, K.; Han, J.; Jian, X.; Wang, J. Synthesis and Thermal Properties of an Acetylenic Monomer Containing Boron and Silicon. *RSC Adv.* **2016**, *6*, 88403–88410. DOI: https://doi.org/10.1039/C6RA19410A

(S7) Kishimoto, K.; Suzawa, T.; Yokota, T.; Mukai, T.; Ohno, H.; Kato, T. Nano-Segregated Polymeric Film Exhibiting High Ionic Conductivities. *J. Am. Chem. Soc.* **2005**, *127*, 15618–15623. DOI: https://doi.org/10.1021/ja0549594

(S8) Korich, A. L.; Clarke, K. M.; Wallace, D.; Iovine, P. M. Chemical Modification of a Lignin Model Polymer via Arylboronate Ester Formation under Mild Reaction Conditions. *Macromolecules* **2009**, *42*, 5906–5908. DOI: https://doi.org/10.1021/ma901146b

(S9) Fulmer, G. R.; Miller, A. J. M.; Sherden, N. H.; Gottlieb, H. E.; Nudelman, A.; Stoltz, B. M.; Bercaw, J. E.; Goldberg, K. I. NMR Chemical Shifts of Trace Impurities: Common Laboratory Solvents, Organics, and Gases in Deuterated Solvents Relevant to the Organometallic Chemist. *Organometallics* **2010**, *29*, 2176–2179.

(S10) Melhuish, W. H. Quantum Efficiencies of Fluorescence of Organic Substances: Effect of Solvent and Concentration of the Fluorescent Solute. *J. Phys. Chem.* **1961**, *65*, 229–235. DOI: https://doi.org/10.1021/j100820a009

(S11) Valeur, B.; Berberan-Santos, N. M. *Molecular Fluorescence. Principles and Applications*, 2nd Ed.; Wiley-VCH, Weinheim, 2012.

(S12) Lakowicz, J. *Principles of Fluorescence Spectroscopy*, 3rd Ed.; Springer-Verlag, New York, **2006**.
